# Angiotensin‐(1–7) Alleviates Isoproterenol‐Induced Cardiac Hypertrophy by Suppressing Autophagy and Apoptosis Through the Synergistic Action of Mas Receptor and Angiotensin II Type 2 Receptor

**DOI:** 10.1111/apha.70200

**Published:** 2026-03-26

**Authors:** Xiaomei Wang, Fei Guo, Xiaoqian Wang, Yu Guo, Siyao Fan, Lan Hong, Honghua Jin

**Affiliations:** ^1^ College of Pharmacy Yanbian University Yanji China; ^2^ Department of Physiology and Pathophysiology, College of Medicine Yanbian University Yanji China; ^3^ Department of Pharmacy, Yanbian University Hospital Yanbian University Yanji China

**Keywords:** angiotensin II type‐2 receptor, angiotensin‐(1–7), apoptosis, autophagy, cardiac hypertrophy, mas receptor

## Abstract

**Aim:**

The aim of this study is to determine whether Angiotensin‐(1–7) [Ang‐(1–7)] alleviates isoproterenol (ISO)–induced cardiac hypertrophy by suppressing excessive autophagy and apoptosis through coordinated Mas receptor (MasR) and angiotensin II type‐2 receptor (AT_2_R) signaling, and to elucidate the underlying mechanisms.

**Methods:**

ISO‐induced hypertrophy was established in mice and assessed by echocardiography, histology, and hypertrophic markers. H9c2 cardiomyocytes were exposed to ISO and treated separately with A‐779 (MasR antagonist), PD123319 (AT_2_R antagonist), and a combination of both receptor antagonists. Receptor interplay was examined using pharmacological blockade and co‐immunoprecipitation. Autophagy and apoptosis were evaluated by transmission electron microscopy and TUNEL.

**Results:**

Ang‐(1–7) attenuated ventricular dysfunction, myocardial enlargement, and upregulation of hypertrophic markers in mice with ISO‐induced hypertrophy. Pharmacological inhibition with A‐779 and PD123319 revealed that Ang‐(1–7) actions require reciprocal regulation between MasR and AT_2_R. Both receptors synergistically contributed to the anti‐apoptotic effect, while the anti‐autophagic response was mediated predominantly by MasR. Transmission electron microscopy and TUNEL staining confirmed that Ang‐(1–7) treatment alleviated excessive autophagy and apoptosis in cardiomyocytes. Furthermore, experiments with dual receptor antagonists and co‐immunoprecipitation showed an interaction between MasR and AT_2_R, supporting their coordinated signaling role in cardiac protection.

**Conclusion:**

Ang‐(1–7) ameliorates ISO‐induced cardiac hypertrophy by suppressing excessive autophagy and apoptosis via synergistic MasR–AT_2_R signaling. Receptor crosstalk may represent a therapeutic entry point for pathological hypertrophy.

## 1 Introduction

Cardiac hypertrophy is a frequent pathological adaptation of the heart to adverse stimuli such as hypertension, valvular disease, and excessive neurohumoral activation. While initially compensatory, persistent hypertrophic remodeling progresses to ventricular dilation, fibrosis, and impaired contractility, thereby markedly elevating the risk of heart failure and sudden cardiac death [1]. Clinical pharmacological strategies—including angiotensin‐converting enzyme inhibitors, angiotensin receptor blockers, and β‐blockers—offer partial symptomatic relief but fail to completely arrest pathological progression [2]. Therefore, a deeper understanding of the molecular mechanisms underlying cardiac hypertrophy and the identification of novel therapeutic targets are of critical importance for the development of more effective preventive and therapeutic strategies, ultimately improving clinical outcomes for affected patients.

Ang‐(1–7) is a bioactive heptapeptide generated from angiotensin II (Ang II) through hydrolysis by angiotensin‐converting enzyme 2 (ACE2), as a critical component of the renin–angiotensin–aldosterone system (RAAS). Unlike the vasoconstrictive, pro‐fibrotic, and pro‐hypertrophic effects mediated by the classical Ang II/AT_1_R axis, Ang‐(1–7) predominantly exerts vasodilatory, anti‐inflammatory, anti‐fibrotic, anti‐proliferative, anti‐thrombotic actions through its specific G protein‐coupled receptor (GPCR), MasR [3, 4]. Experimental studies have demonstrated that Ang‐(1–7), its analogues, ACE2 activators, and MasR agonists provide cardiovascular protection in diverse models of hypertension [5, 6, 7], myocardial infarction, ischemia–reperfusion injury, heart failure, and cardiac hypertrophy [8, 9, 10]. The underlying mechanisms include enhancement of immune cell phagocytic capacity, promotion of anti‐inflammatory and reparative processes, and inhibition of pathological hypertrophy and fibrosis through MasR/Sirt3‐mediated FoxO3a deacetylation, which upregulates SOD2 and alleviates oxidative stress [7, 11]. These findings establish the Ang‐(1–7)/MasR axis as a pivotal counter‐regulatory pathway against RAAS overactivation. Moreover, both MasR and AT_2_R belong to the GPCR family, binding Ang‐(1–7) and Ang II, respectively, to elicit distinct biological effects [12]. GPCRs can undergo conformational changes by forming homo‐ or heterodimers, thereby modulating ligand binding, signal transduction, and downstream functional responses—an attribute of considerable pathophysiological significance in disease progression [13]. Of note, AT_2_R has been recognized for its cardiovascular protective role, largely by antagonizing AT_1_R signaling [14]. Importantly, existing studies have confirmed functional interactions among MasR, AT_1_R, and AT_2_R in the heart, indicating complex receptor cross‐regulation within the RAAS [15]. Therefore, the protective effects of Ang‐(1–7) may involve synergistic signaling with AT_2_R rather than acting solely through MasR. Although both MasR and AT_2_R have been implicated in treating hypertension, evidence for their synergistic antihypertensive effects in the cardiac context remains scarce.

Pathological cardiac hypertrophy is driven by multiple mechanisms, among which dysregulation of autophagy and apoptosis plays a pivotal role [16, 17]. Autophagy, a fundamental process of cellular self‐renewal and quality control, is essential for maintaining cardiomyocyte homeostasis [18]. However, excessive activation of autophagy or impaired autophagic flux is closely associated with cardiomyocyte death and functional deterioration [19]. Apoptosis, as the primary form of programmed cell death, directly contributes to myocardial dysfunction through the progressive loss of cardiomyocytes. Increasing evidence suggests that autophagy and apoptosis engage in intricate crosstalk, sharing regulatory mediators such as Bcl‐2 family proteins, and that the status of autophagy critically shapes cellular susceptibility to apoptotic stimuli [20, 21]. In β‐adrenergic agonist–induced cardiac hypertrophy models, excessive sympathetic stimulation has been shown to simultaneously trigger aberrant autophagy and apoptosis, collectively exacerbating myocardial injury and remodeling [22]. It is worth noting that, although existing studies have shown that Ang‐(1–7) can regulate autophagy and exert an anti‐apoptotic effect in kidney diseases [23, 24], it is still unclear whether it provides cardioprotective effects through the synergistic action of MasR and AT_2_R.

On this basis, we hypothesized that the protective effect of Ang‐(1–7) against isoproterenol‐induced cardiac hypertrophy is not mediated solely by MasR, but rather relies on the synergistic interplay between MasR and AT_2_R, which together orchestrate the regulation of autophagy and apoptosis. To our knowledge, this evidence suggests that receptor crosstalk contributes to the cardioprotective actions of Ang‐(1–7), thereby providing a novel conceptual framework for understanding its mechanisms and pointing to new therapeutic opportunities for targeting pathological hypertrophy.

## 2 Results

### 2.1 Ang‐(1–7) Exhibits Globular Solubility and Low Cytotoxicity

**FIGURE 1:**
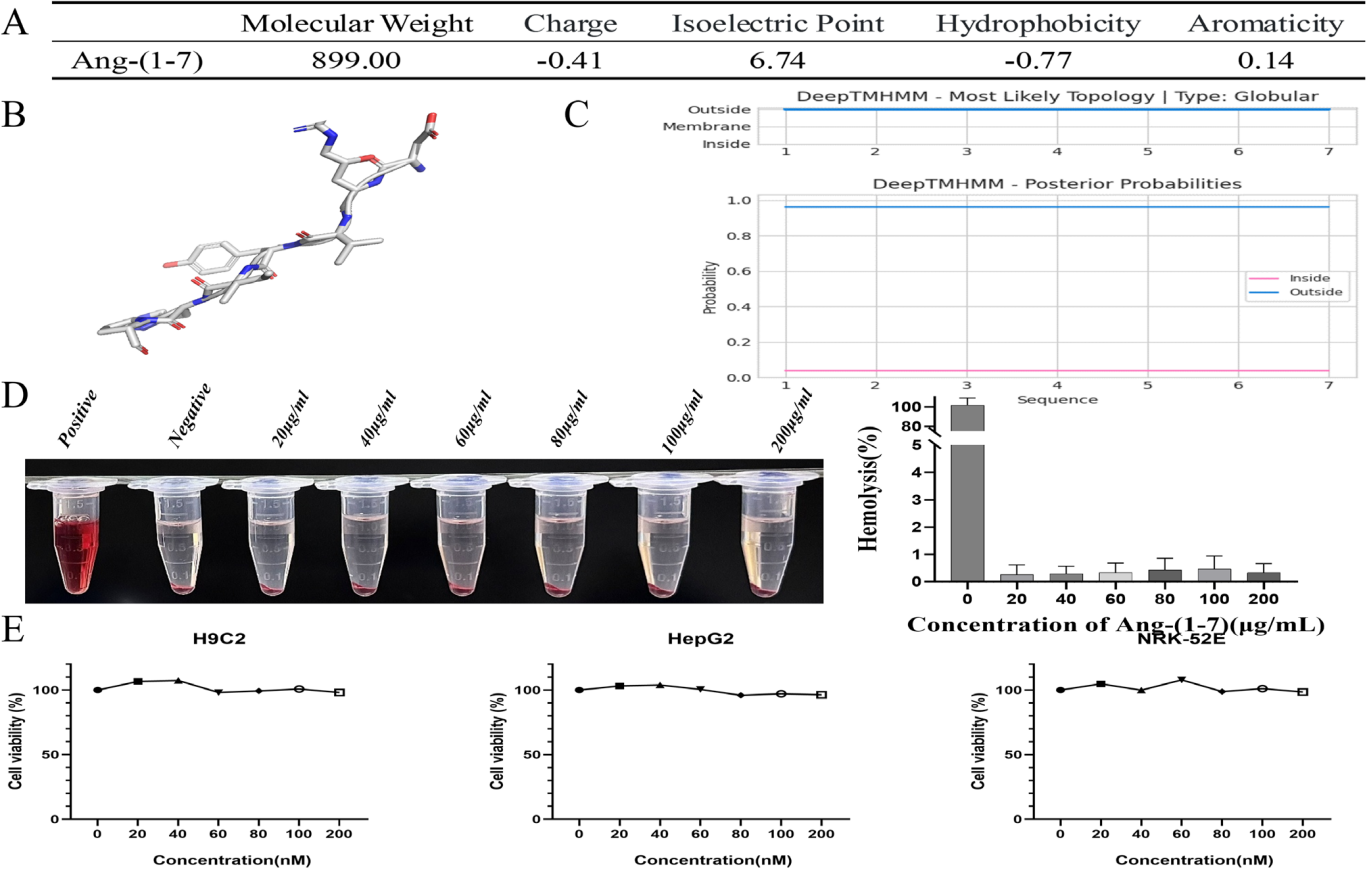
Physical and chemical properties and biosafety assessment of Ang‐(1–7). (A) Key molecular properties: molecular weight (MW), net charge, isoelectric point (pI), hydrophobicity (GRAVY index), and aromaticity. (B, C) Topological structure predicted by Deep TMHMM, showing a spherical soluble conformation (no transmembrane helices) and predicted functional sites (red‐intracellular; blue‐extracellular). (D) Hemolytic activity (*n* = 6). (E) Cytotoxicity assessment in H9c2, HepG2, and NRK‐52E cell lines (*n* = 6). Cell viability was determined using the Cell Counting Kit‐8 (CCK‐8) assay. The data is expressed as an mean ± standard deviation (SD).

To assess the biosafety of Ang‐(1–7), both bioinformatic and experimental analyses were performed. Physicochemical profiling identified Ang‐(1–7) as a small, hydrophilic peptide (MW: 899.00 Da, pI: 6.74, charge: −0.41, hydrophobicity: −0.77, aromaticity: 0.14) (Figure 1A). DeepTMHMM analysis confirmed its globular soluble structure without transmembrane domains (Figure 1B,C). Hemolysis assays revealed negligible activity (< 5% at 200 μg/mL) (Figure 1D). Similarly, CCK‐8 assays demonstrated minimal cytotoxicity, with > 80% viability in H9c2 and HepG2 cells and no significant toxicity in NRK‐52E cells up to 200 μg/mL (Figure 1E). Only supraphysiological concentrations (> 100 μg/mL) induced mild dose‐dependent effects. These findings indicate that Ang‐(1–7) possesses favorable biosafety with low cytotoxicity.

### 2.2 Ang‐(1–7) Attenuates ISO‐Induced Cardiac Hypertrophy and Fibrosis

**FIGURE 2:**
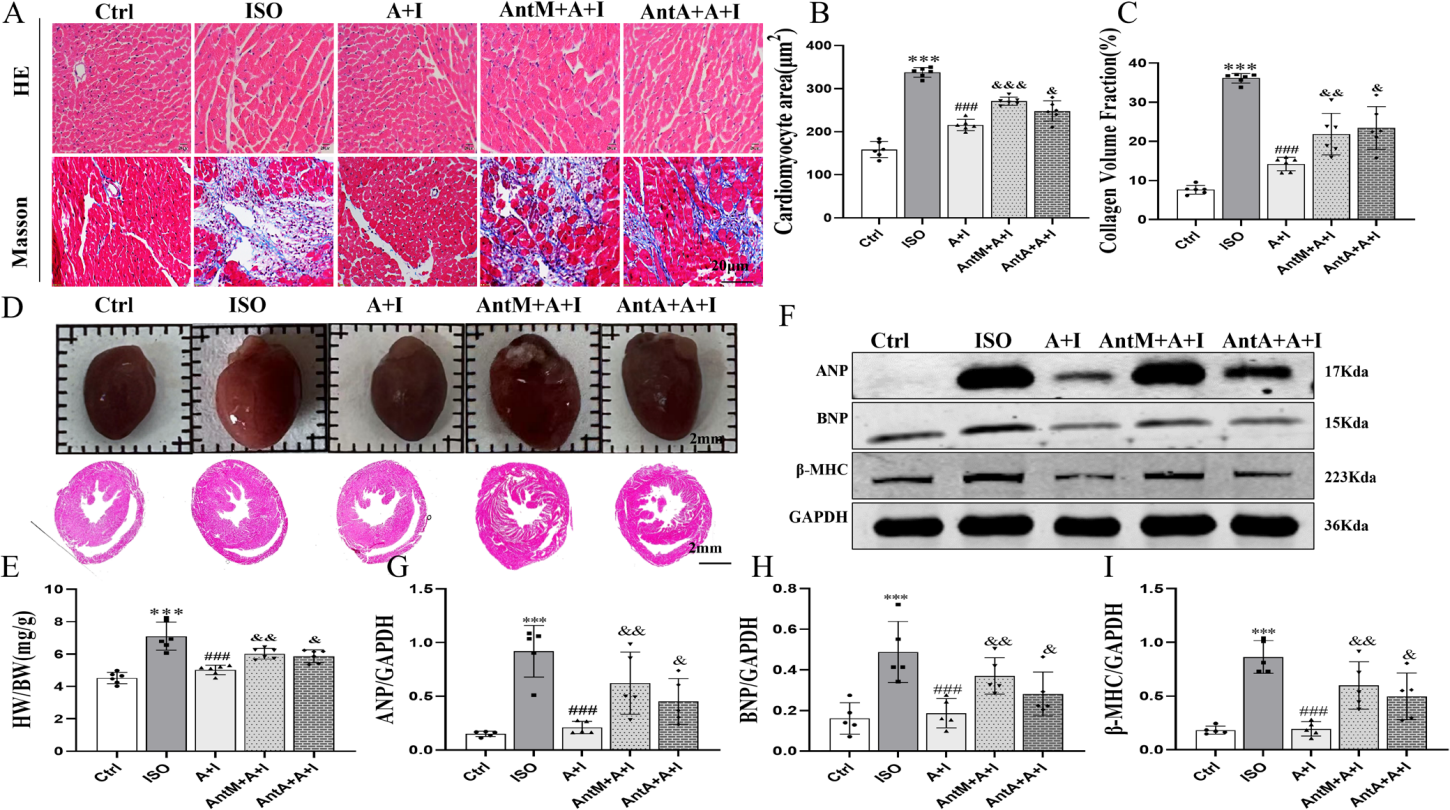
Effects of Ang‐(1–7) on isoproterenol (ISO)‐induced cardiac hypertrophy and fibrosis. (A) HE staining and Masson staining of myocardial sections (scale bar = 20 μm). (B, C) Quantitative analysis of myocardial cell cross‐sectional area and collagen volume fraction. (D, E) Macroscopic morphology and cross‐sectional observation of the heart, with measurement of heart weight/body weight ratio (HW/BW) (scale bar = 2 mm). (F) Western blot analysis of ANP, BNP, and β‐MHC expression. (G–I) Quantitative analysis of band intensity normalized to GAPDH. The data is expressed as an mean ± standard deviation (SD) (*n* = 5). A + I, Ang‐(1–7) + ISO; AntM+A + I, A‐779 + Ang‐(1–7) + ISO; AntA+A + I, PD123319 + Ang‐(1–7) + ISO. ***p < 0.001 versus Ctrl; ^###^ *p* < 0.001 versus ISO; ^&&&^ *p* < 0.001, ^&&^ *p* < 0.01, ^&^ *p* < 0.05 versus Ang‐(1–7) + ISO.

Histological and molecular analyses demonstrated that ISO markedly increased cardiomyocyte cross‐sectional area (Figure S1A) and collagen deposition, indicative of hypertrophy and fibrosis (Figure 2A,C). Panoramic HE scans further confirmed cardiac enlargement with elevated HW/BW ratios (Figure 2D,E). Protein levels of hypertrophy markers ANP, BNP, and β‐MHC were also strongly upregulated (Figure 2F–I). Ang‐(1–7) administration significantly reduced these pathological changes, decreasing cell area, collagen fraction, HW/BW ratio, and hypertrophic marker expression. Importantly, A‐779 or PD123319 partially reversed these benefits, suggesting that both MasR and AT_2_R contribute to the cardioprotective actions of Ang‐(1–7).

### 2.3 Ang‐(1–7) Improves ISO‐Induced Ventricular Remodeling and Functional Impairment via MasR and AT_2_R

**FIGURE 3:**
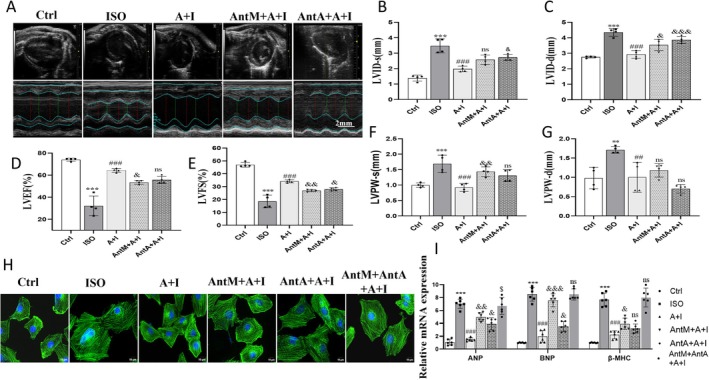
Ang‐(1–7) improves ISO‐induced ventricular remodeling and dysfunction via MasR and AT_2_R. (A) Representative M‐mode echocardiograms from each group (scale bar = 2 mm). (B, C) Quantification of left ventricular internal diameters at systole (LVIDs) and diastole (LVIDd). (D, E) Left ventricular ejection fraction (LVEF%) and fractional shortening (LVFS%). (F, G) Left ventricular posterior wall thickness at systole (LVPWs) and diastole (LVPWd). (H) Representative immunofluorescence images of cardiomyocytes (green, α‐actinin; blue, nuclei; scale bar = 10 μm). (I) Relative mRNA expression of ANP, BNP, and β‐MHC (*n* = 6). The data is expressed as an mean ± standard deviation (SD) (*n* = 5). A + I, Ang‐(1–7) + ISO; AntM + A + I, A‐779 + Ang‐(1–7) + ISO; AntA + A + I, PD123319 + Ang‐(1–7) + ISO; AntM + AntA + A + I, A‐779 + PD123319 + Ang‐(1–7) + ISO. ***p < 0.001, **p < 0.01 versus Ctrl; ^###^ *p* < 0.001, ^##^ *p* < 0.01 versus ISO; ^&&&^ *p* < 0.001, ^&&^ *p* < 0.01, ^&^ *p* < 0.05 versus Ang‐(1–7) + ISO, ^&^ *p* < 0.05 versus ISO.

Echocardiography revealed that ISO caused ventricular dilation (increased LVID,s and LVID,d), posterior wall thickening, and reduced LVEF and LVFS (Figure 3A–G). Ang‐(1–7) significantly improved these parameters, restoring cardiac geometry and systolic performance. These protective effects were blunted by either A‐779 or PD123319. Immunofluorescence staining corroborated these findings, showing reduced cardiomyocyte size following Ang‐(1–7), which was partially reversed by receptor blockade. In the dual receptor antagonist group, the therapeutic effect of Ang‐(1–7) was completely abolished (Figure 3H, Figure S1). Additionally, we verified that neither A‐779 nor PD123319 alone exhibited anti‐hypertrophic physiological effects, confirming that the antagonists themselves did not interfere with the experimental results (Figure S9). Molecular and protein results showed that Ang‐(1–7) downregulated isoproterenol‐induced ANP, BNP, and β‐MHC expression, while single receptor antagonists attenuated this effect. Following dual receptor antagonism, expression levels showed no significant difference compared to the isoproterenol group (Figure 3I, Figure S2A,B). These results indicate that Ang‐(1–7) can treat ISO‐induced myocardial dysfunction in mice. While single receptor antagonists showed limited efficacy, combined administration restored expression to baseline levels, suggesting MasR and AT_2_R play critical roles in ISO‐induced cardiac hypertrophy.

### 2.4 Ang‐(1–7) Restores and Stabilizes MasR and AT_2_R Expression in ISO‐Treated Cardiomyocytes

**FIGURE 4:**
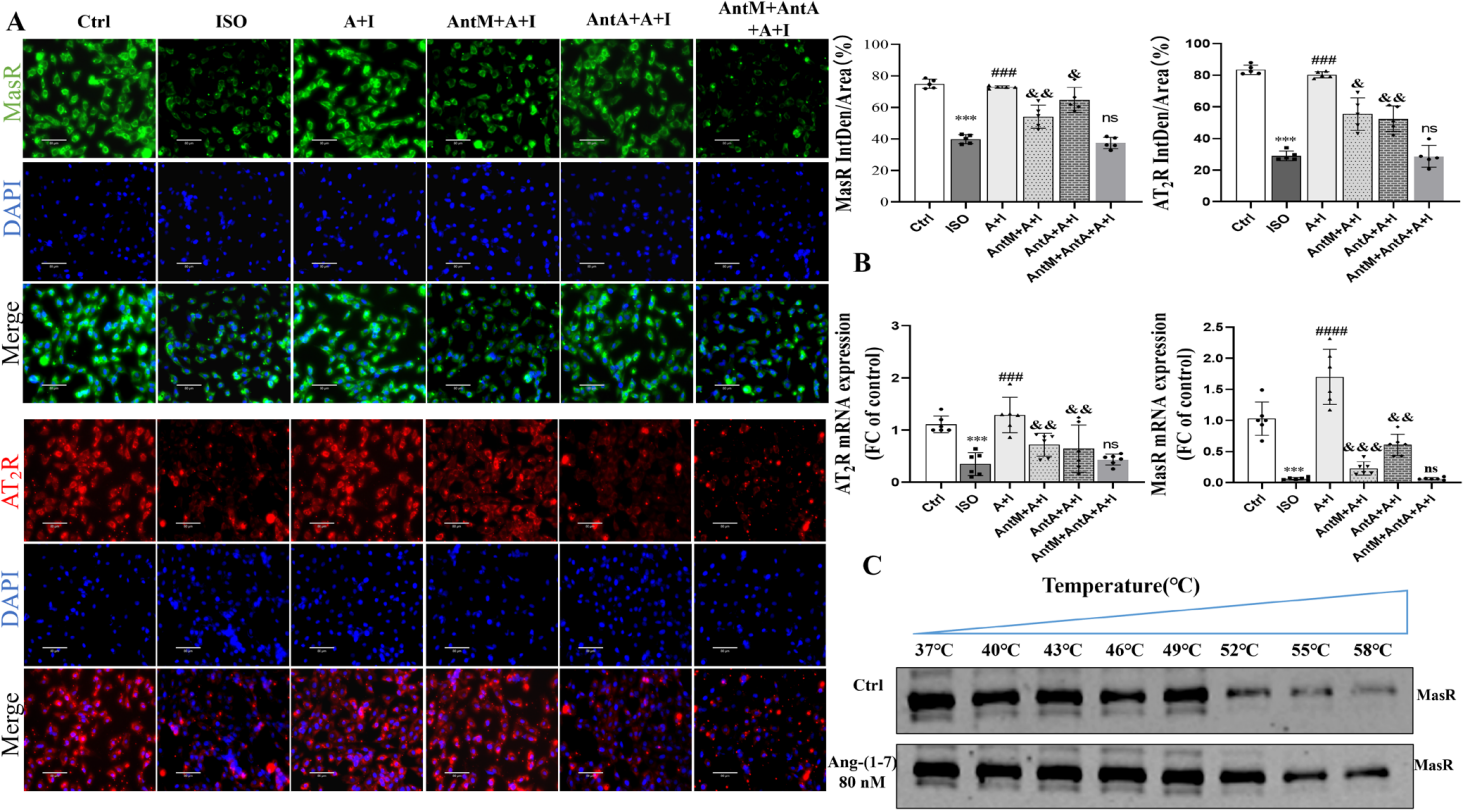
Ang‐(1–7) regulates the expression of MasR and AT_2_R in H9c2 cardiomyocytes and exhibits cross‐inhibition with receptor antagonists. (A) Immunofluorescence images and immunofluorescence intensity of MasR (green), AT_2_R (red), and DAPI (blue) in H9c2 cells from different treatment groups, reflecting receptor expression levels (scale bar = 80 μm) (*n* = 5). (B) Shows the expression of AT_2_R and MasR mRNA in H9c2 cells (*n* = 6). (C) Analyzes MasR expression and relative thermal stability in cardiomyocytes at different temperatures (37°C–58°C). The data is expressed as an mean ± standard deviation (SD) (*n* = 5). A + I, Ang‐(1–7) + ISO; AntM + A + I, A‐779 + Ang‐(1–7) + ISO; AntA + A + I, PD123319 + Ang‐(1–7) + ISO; AntM + AntA + A + I, A‐779 + PD123319 + Ang‐(1–7) + ISO. ***p < 0.001, *p < 0.05 versus Ctrl; ^####^ *p* < 0.0001, ^###^ *p* < 0.001 versus ISO; ^&&&^ *p* < 0.001, ^&&^ *p* < 0.01, ^&^ *p* < 0.05 versus Ang‐(1–7) + ISO.

Immunofluorescence demonstrated that ISO reduced MasR and AT_2_R expression, whereas Ang‐(1–7) restored their levels (Figure 4A). Real time PCR confirmed upregulation of both receptors after Ang‐(1–7) treatment (Figure 4B). Western blotting revealed that co‐treatment with dual receptor antagonists resulted in a more pronounced reduction in MasR and AT_2_R expression compared to single receptor antagonists, suggesting both receptors are modulated by Ang‐(1–7) and interact with each other (Figure S4). Interestingly, A‐779 suppressed MasR expression and concomitantly reduced AT_2_R levels, while PD123319 inhibited AT_2_R and partially reduced MasR, indicating reciprocal regulation. Thermal shift assays revealed that Ang‐(1–7) enhanced MasR stability under stress, with marked reduction occurring only beyond 58°C (Figure 4C, Figure S3). These results suggest that Ang‐(1–7) not only upregulates MasR and AT_2_R but also stabilizes MasR under stress, reflecting a potential ligand–receptor physical association.

### 2.5 Ang‐(1–7) Enhances MasR–AT_2_R Crosstalk In Vivo

**FIGURE 5:**
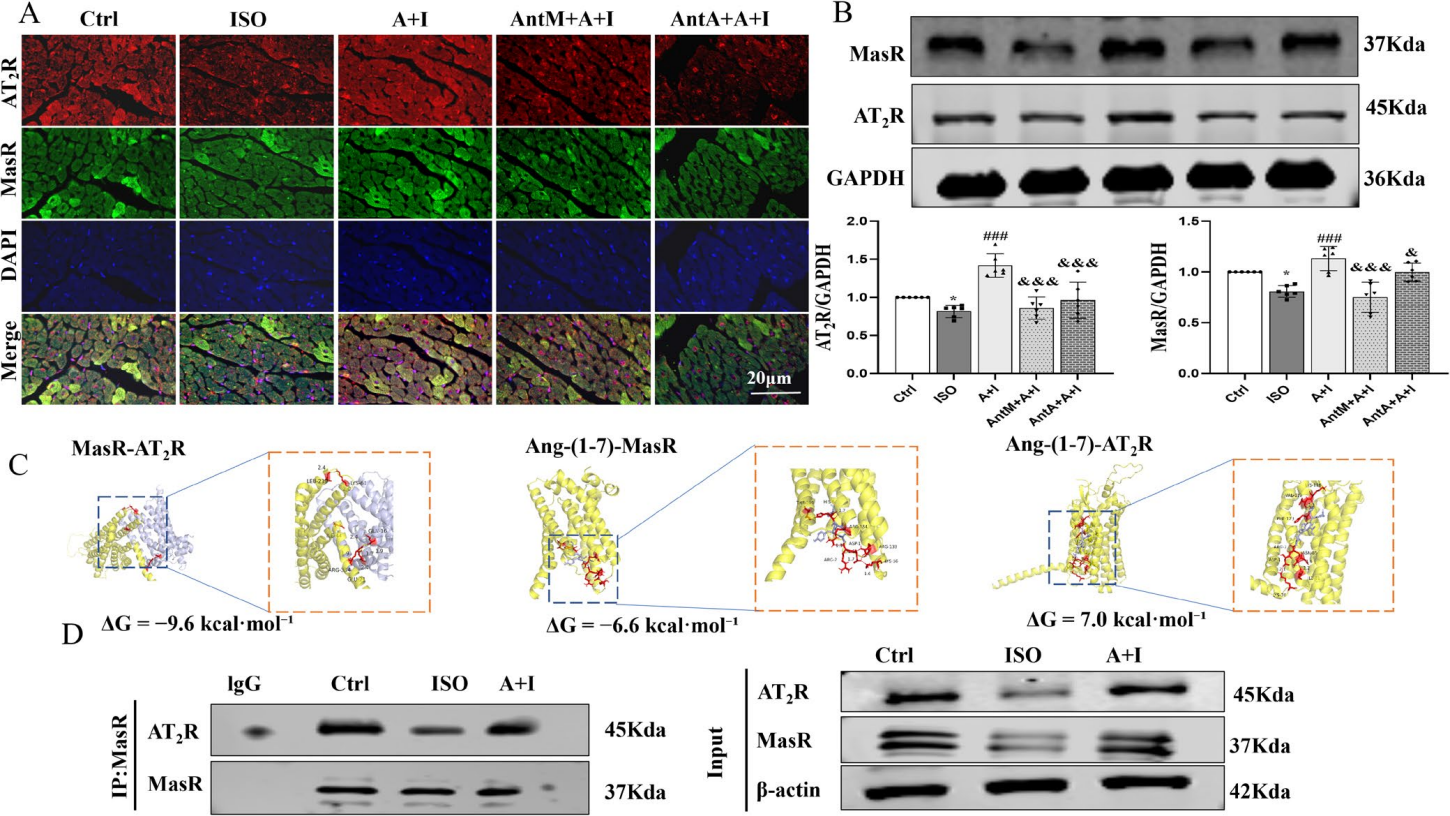
Ang‐(1–7) regulates the expression of MasR and AT_2_R and their heterodimer formation. (A) Immunofluorescence staining images of MasR (green), AT_2_R (red), and DAPI (blue) in myocardial tissue from mice in each treatment group. Arrows indicate the localization of receptors in the myocardium (scale bar = 20 μm) (*n* = 5). (B) Protein expression and quantitative analysis of MasR and AT_2_R in myocardial tissue (*n* = 6). (C) Molecular docking simulations showing the interactions between MasR and AT_2_R, as well as between Ang‐(1–7) and the receptors, along with the binding energies (ΔG) for each interaction. (D) Immunoprecipitation (Co‐IP): Interaction between MasR and AT_2_R in cardiac muscle tissue. The data is expressed as an mean ± standard deviation (SD). A + I, Ang‐(1–7) + ISO; AntM + A + I, A‐779 + Ang‐(1–7) + ISO; Ant A + A + I, PD123319 + Ang‐(1–7) + ISO. *p < 0.05 versus Ctrl; ^###^ *p* < 0.001 versus ISO; ^&&&^ *p* < 0.001, ^&^ *p* < 0.05 versus Ang‐(1–7) + ISO.

In vivo studies showed that ISO markedly decreased MasR and AT_2_R expression in mouse myocardium, while Ang‐(1–7) restored their levels and promoted colocalization (Figure 5A). Western blotting confirmed upregulation of both receptors after Ang‐(1–7) treatment, which was partially reversed by A‐779 or PD123319 (Figure 5B). Molecular docking indicated that MasR and AT_2_R could form a heterodimer with strong binding affinity (ΔG = −9.6 kcal/mol). Ang‐(1–7) exhibited strong binding to MasR (ΔG = −6.6 kcal/mol) but not to AT_2_R (ΔG = 7.0 kcal/mol) (Figure 5C). Co‐immunoprecipitation further demonstrated that ISO impaired MasR–AT_2_R interaction, whereas Ang‐(1–7) enhanced it significantly (Figure 5D). Together, these findings support that Ang‐(1–7) regulates MasR and AT_2_R expression and reinforces their interaction in vivo.

### 2.6 Ang‐(1–7) Inhibits ISO‐Induced Excessive Autophagy and Apoptosis via MasR and AT_2_R

**FIGURE 6:**
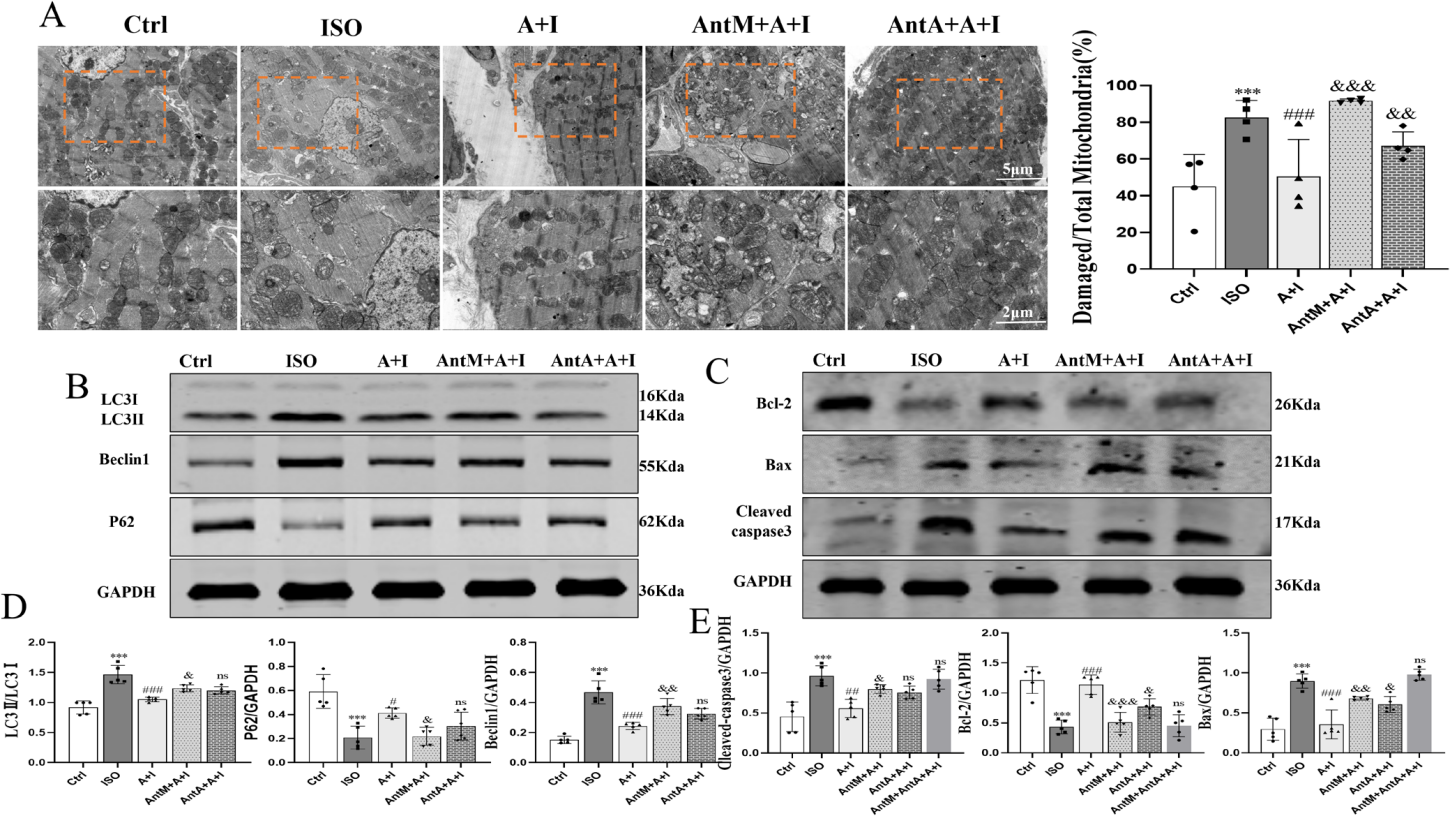
Ang‐(1–7) attenuates ISO‐induced excessive autophagy and apoptosis in vivo by regulating MasR and AT_2_R. (A) Transmission electron microscopy (TEM) images showing autophagosomes, lysosomes, and damaged mitochondria in cardiomyocytes; quantification of damaged mitochondria is shown (scale bar = 5 μm, 2 μm *n* = 4). (B) Western blot analysis of autophagy‐related proteins LC3‐II/I, Beclin1, and P62 (*n* = 5). (C) Western blot analysis of apoptosis‐related proteins Bcl‐2, Bax, and cleaved caspase‐3 (*n* = 5). (D, E) Quantitative densitometry analysis of autophagy‐ and apoptosis‐related proteins normalized to GAPDH. The data is expressed as an mean ± standard deviation (SD). A + I, Ang‐(1–7) + ISO; AntM + A + I, A‐779 + Ang‐(1–7) + ISO; Ant A + A + I, PD123319 + Ang‐(1–7) + ISO. ***p < 0.001 versus Ctrl; ^###^ *p* < 0.001, ^#^ *p* < 0.05 versus ISO; ^&&&^ *p* < 0.001, ^&&^ *p* < 0.01, ^&^ *p* < 0.05 versus Ang‐(1–7) + ISO.

Transmission electron microscopy showed that ISO induced abundant autophagosomes and mitochondrial swelling, while Ang‐(1–7) reduced autophagosome formation and preserved mitochondrial integrity. This protective effect was abolished by A‐779 but only mildly affected by PD123319 (Figure 6A). Immunoblotting revealed that ISO increased the LC3‐II/LC3‐I ratio and Beclin1 while reducing p62, changes reversed by Ang‐(1–7). A‐779 blocked these effects, whereas PD123319 had limited impact, indicating that MasR primarily mediates autophagy regulation (Figure 6B). In contrast, both antagonists abolished the anti‐apoptotic effects of Ang‐(1–7), which reduced Bax and cleaved caspase‐3 while increasing Bcl‐2 (Figure 6C–E). These results demonstrate that Ang‐(1–7) suppresses excessive autophagy mainly through MasR, whereas both MasR and AT_2_R cooperate to inhibit apoptosis.

### 2.7 Ang‐(1–7) Regulates Autophagic Flux and Apoptosis in H9c2 Cells Through MasR and AT_2_R

**FIGURE 7:**
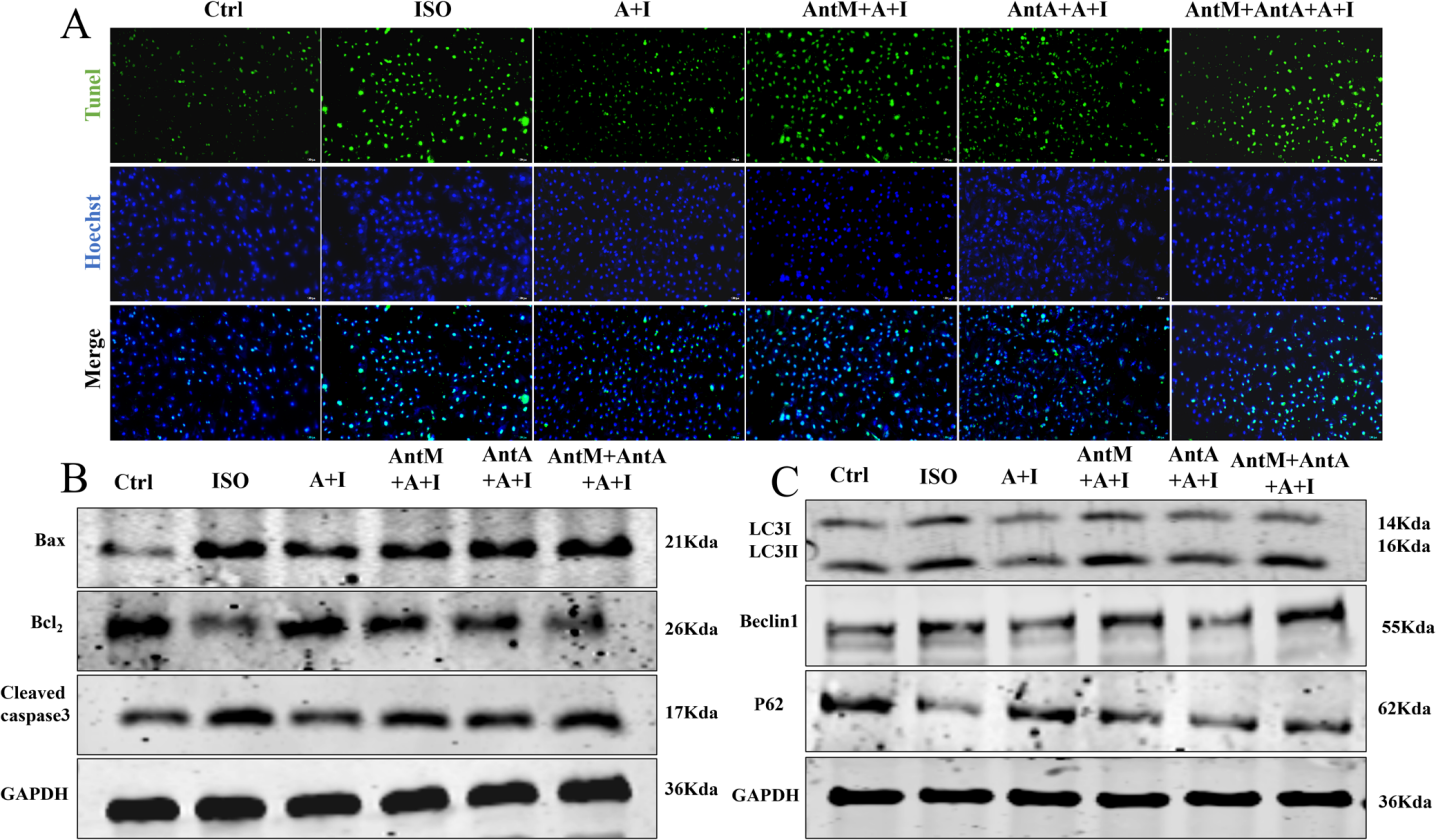
Ang‐(1–7) regulates autophagic flux and apoptosis in H9c2 cardiomyocytes through MasR and AT_2_R. (A) Representative images of TUNEL immunofluorescence staining (green fluorescence labels apoptotic cells) and Hoechst nuclear staining (blue) (*n* = 6), with the Merge panel showing their overlay. (B) Western blot analysis of apoptosis related proteins (Bax, Bcl_2_, Cleaved caspase‐3) expression levels, with GAPDH as the internal control protein (*n* = 3). (C) Western blot analysis of autophagy‐related proteins (LC3‐II, Beclin1, P62) expression levels, with GAPDH as the internal control protein (*n* = 3). The data is expressed as an mean ± standard deviation (SD). A + I, Ang‐(1–7) + ISO; AntM + A + I, A‐779 + Ang‐(1–7) + ISO; Ant A + A + I, PD123319 + Ang‐(1–7) + ISO; AntM + AntA + A + I, A‐779 + PD123319 + Ang‐(1–7) + ISO.

In H9c2 cardiomyocytes, ISO reduced p62 fluorescence, indicating enhanced autophagic flux (Figure S5A). Ang‐(1–7) restored p62 aggregation and reduced LC3‐II/LC3‐I and Beclin1 while upregulating p62, confirming its role in restraining autophagy (Figure S5B). These effects were partially reversed by A‐779 or PD123319, with MasR blockade showing stronger antagonism (Figure 7C). TUNEL assays demonstrated increased apoptosis following ISO, which was significantly reduced by Ang‐(1–7); receptor antagonists attenuated this effect (Figure 7A). Western blotting corroborated these findings, showing that Ang‐(1–7) reversed ISO‐induced Bax and cleaved caspase‐3 elevation while restoring Bcl‐2, with both antagonists partially blocking these effects. Both antagonists partially blocked these effects, and the dual receptor blocker group completely reversed the anti‐apoptotic action of Ang‐(1–7) (Figure 7B, Figure S5C). In summary, Ang‐(1–7) constrains excessive autophagy primarily via MasR, while both MasR and AT_2_R synergistically mediate its anti‐apoptotic action, collectively contributing to cardiomyocyte homeostasis.

## 3 Discussion

In this study, we demonstrate that the cardioprotective effect of Ang‐(1–7) against pathological hypertrophy is not mediated exclusively by MasR. Rather, it requires the synergistic interaction between MasR and AT_2_R, which cooperatively suppress excessive autophagy and apoptosis. Specifically, MasR acts as the predominant regulator of autophagy, whereas AT_2_R contributes alongside MasR to apoptosis regulation, highlighting their cooperative yet distinct roles in cardioprotection. As a pathological driver, cardiac hypertrophy contributes to the progression of major cardiovascular disorders, including hypertension and myocardial infarction. If left uncontrolled, it can culminate in heart failure with irreversible consequences. Thus, the prevention and treatment of cardiac hypertrophy hold significant clinical value for cardiovascular health. Within the endogenous regulatory network, neurohormonal signaling plays a central role, particularly the protective arm of the RAAS. In this context, the Ang‐(1–7)/MasR and Ang II/AT_2_R axes have been reported to exert anti‐atherosclerotic, anti‐arrhythmic, and anti‐remodeling effects [25, 26, 27].

We validated the ISO‐induced cardiac hypertrophy model using histological and molecular markers. Hypertrophic markers ANP, BNP, and β‐MHC were robustly upregulated, consistent with previous reports [28]. Echocardiography confirmed that ISO‐treated mice exhibited increased ventricular diameters and impaired systolic function. Importantly, the MasR antagonist A‐779 did not completely eliminate the antihypertrophic effect of Ang‐(1–7), while the AT_2_R antagonist PD123319 only partially attenuated this effect. Co‐administration of MasR and AT_2_R antagonists achieved complete blockade, indicating that both MasR and AT_2_R participate in the protective process. This aligns with prior evidence supporting functional interactions between MasR and AT_2_R. Indeed, knockout studies suggest that Ang‐(1–7) regulates inflammatory and metabolic disorders via MasR activation and AT_1_R inhibition, whereas AT_2_R can act independently in renal protection [29, 30, 31]. Unlike studies in the kidney or liver, where receptor‐specific roles have been delineated, cardiac studies remain limited and have largely focused on MasR alone [25, 32]. To address this gap, we employed dual receptor antagonists and demonstrated that both MasR and AT_2_R are expressed in the myocardium and participate in Ang‐(1–7)–mediated cardioprotection.

Mechanistically, we provide novel evidence that Ang‐(1–7) enhances MasR–AT_2_R crosstalk. Molecular docking suggests that Ang‐(1–7) directly binds to the Mas receptor, whereas the AT_2_R does not interact with Ang‐(1–7). Therefore, the antihypertrophic effect of AT_2_R may arise from its formation of a heterodimer with MasR. Previous studies have also reported that in the kidney, the Mas receptor and AT_2_R can form a high‐affinity, stable heterodimer through disulfide bonds. Co‐immunoprecipitation confirmed their physical interaction in myocardial tissue, which was impaired by ISO but restored by Ang‐(1–7). This finding is consistent with previous work demonstrating that MasR–AT_2_R heterodimerization generates distinct signaling profiles compared with single receptor activation [33]. Moreover, reciprocal regulation between receptors was observed at both the protein and transcript levels, A‐779 reduced AT_2_R expression, while PD123319 partially inhibited MasR, a hallmark of heterodimer formation [34, 35]. Unlike studies utilizing AT_2_R agonists, we employed dual antagonists to validate the functional interdependence of these receptors in vivo [36].

Cardiac injury is often accompanied by processes of autophagy and apoptosis, which is consistent with existing evidence [37]. Excessive autophagy exacerbates cardiomyocyte injury and triggers apoptosis, thereby further increasing cardiac burden. ISO activates β‐adrenergic receptors, elevates intracellular cAMP levels, and subsequently inhibits mTOR through PKA, initiating autophagic processes [38]. Therefore, inhibition of autophagy is considered beneficial for the prevention and treatment of cardiovascular injury. In agreement with previous reports [39, 40, 41]. Our findings partially overlap with the latter, but we found that Ang‐(1–7) does not exert its effects by directly inhibiting autophagy, but rather by activating MasR, thereby preventing the pathological damage caused by ISO. Electron microscopy results also corroborate this. Furthermore, after administering two antagonists, it was found that MasR is the key receptor regulating autophagy, while AT_2_R cannot inhibit the anti‐hypertrophic effects of overly activated autophagy, This aligns with the finding that the anti‐autophagy effect mediated by AT_2_R is not blocked by its own antagonist PD123319 [42]. However, in a hypertension model, the anti‐autophagy effect of Ang‐(1–7) can be blocked by the MasR antagonist A‐779 and the AT_2_R antagonist PD123319, which may be due to the fact that we pretreated with the antagonist, preventing ISO from activating autophagy [43]. Additionally, we found that Bcl‐2, which directly interacts with Beclin1, was activated after Ang‐(1–7) administration [44]. This activation occurs through direct binding and inhibition of the activation and mitochondrial localization of the pro‐apoptotic protein Bax, thereby maintaining the integrity of the mitochondrial outer membrane, reducing the release of cytochrome C, and inhibiting the initiation of the caspase cascade, ultimately suppressing apoptosis. To address the demands of drug clinical translation, this study systematically evaluated the toxicity and safety of Ang‐(1–7), with extended validation across multiple cell lines and hemolysis assays, demonstrating the comprehensiveness and innovation of the experimental design [45]. In this study, utilizing a highly human‐heart‐physiology‐mimicking cellular model—cardiomyocytes differentiated from human induced pluripotent stem cells (hiPSC)—we clarifying the expression localization and spatial distribution characteristics of MasR and AT_2_R (Figure S6). This provides a morphological foundation for elucidating how Ang‐(1–7) inhibits processes like myocardial hypertrophy by synergistically regulating these two receptors. Furthermore, it confirms the critical role of MasR–AT_2_R interaction in Ang‐(1–7)‐mediated cardioprotection, thereby offering robust support for advancing this synergistic mechanism into translational medical research.

Furthermore, current evidence indicates that PD123319 exhibits significant receptor‐binding pleiotropy: for instance, Lautner et al. demonstrated in aortic ring experiments with AT_2_R knockout mice that PD123319 still blocks Alamandine‐mediated vasodilation, directly confirming its ability to bind and inhibit Mas‐related G protein‐coupled receptor D (MrgD) [46]. Therefore, in experimental systems co‐expressing AT_2_R and MrgD, relying solely on PD123319's blocking effect cannot accurately distinguish the contributions of these two receptors. To eliminate this confounding factor and ensure the reliability and specificity of conclusions drawn from PD123319‐mediated AT_2_R blockade in this study, we conducted targeted validation in an ISO‐induced hypertrophy model of H9c2 cardiomyocytes. Results showed that the MrgD‐specific agonist alamandine exhibited only a weak antihypertrophic effect, significantly weaker than Ang‐(1–7), and this minimal effect was not blocked by PD123319 (Figures S7A–C and S8A,B). This indicates that under the pathological model conditions and experimental concentrations used in this study, PD123319 does not mediate its effects through MrgD, with its primary target being the AT_2_R.

In conclusion, our findings identify a cardioprotective mechanism whereby Ang‐(1–7) counters ISO‐induced cardiac hypertrophy through concerted signaling between MasR and AT_2_R. Ang‐(1–7) promotes colocalization and reciprocal regulation of these receptors, restores downstream pathways, and ultimately suppresses excessive autophagy and apoptosis, thereby limiting structural remodeling and functional decline. Mechanistically, MasR predominates in autophagy control, whereas both MasR and AT_2_R cooperate to restrain apoptosis, revealing complementary yet specialized roles in cardioprotection. These results refine the counter‐regulatory RAAS paradigm and highlight MasR–AT_2_R receptor crosstalk—rather than single‐receptor activation—as a promising therapeutic entry point for intervention in pathological cardiac hypertrophy.

## 4 Materials and Methods

### 4.1 Mouse Models

Male C57BL/6 mice (6–8 weeks; Yanbian University Animal Research Center) were housed under SPF conditions (12 h light/dark, 22°C–24°C). After 7 days' acclimatization, animals were randomized (blinded assessment) into five groups (*n* = 6 per group) receiving daily Subcutaneous injection for 7 days [28, 47, 48]:Ctrl: saline (Sanlian, Harbin, China); ISO: isoproterenol 5 mg/kg/day (Solarbio, II0200, Beijing, China; dissolved in DMSO); ISO + Ang‐(1–7): Ang‐(1–7) 576 μg/kg/day (MCE, HY‐12403, USA; in distilled water); ISO + Ang‐(1–7) + A‐779: A‐7791148 μg/kg/day (MCE, HY‐P0216, USA); ISO + Ang‐(1–7) + PD123319: PD123319 5 mg/kg/day (MCE, HY‐10259A, USA); ISO + Ang‐(1–7) + PD123319 + A779. Hearts were weighed after euthanasia. All procedures were IACUC‐approved (Yanbian University, YD20250627018).

### 4.2 Hemolysis Assay

Fresh anticoagulated mouse blood was washed (PBS) to clarity, then adjusted to a 2% (v/v) RBC suspension. Aliquots (20 μL) were mixed with Ang‐(1–7) serial dilutions (0–200 μg/mL, PBS). Positive/negative controls: 0.1% Triton X‐100 and PBS, respectively. After 37°C incubation for 2 h and centrifugation, supernatants were read at 540 nm to compute hemolysis (%). Hemolysis (%) = (OD positive − OD negative)/(OD sample − OD negative) × 100%.

### 4.3 Echocardiography

Echocardiography was conducted by an operator masked to group allocation using a Vevo 2100 system (VisualSonics, Toronto, Canada). After treatment, mice were anesthetized with isoflurane (2% induction; 1.0%–1.5% maintenance). M‐mode images were acquired, and left‐ventricular indices—LVEF, LVFS, LVIDs/LVIDd, and LVPWs/LVPWd—were calculated as the mean of three consecutive beats at end‐systole and end‐diastole.

### 4.4 Tissue Processing, Histology, and Morphometry

Left ventricles were fixed in 4% paraformaldehyde (Coolaber, SL18301, China), dehydrated in graded ethanol, cleared in xylene, embedded in paraffin, and sectioned at 4 μm (Leica RM2255, Germany). H&E staining (Solarbio, G1120, China) was performed with hematoxylin, water rinse, differentiation, and 0.5% eosin, followed by dehydration and clearing. For Masson's trichrome (Solarbio, G1346, China), sections were mordanted, rinsed, sequentially stained with azur blue and Mayer's hematoxylin with brief differentiation, then with ponceau fuchsin, phosphomolybdic acid, and aniline blue, followed by weak‐acid rinse, dehydration, and clearing. Slides were mounted in neutral resin and examined by bright‐field microscopy; cardiomyocyte cross‐sectional area (CSA) and collagen volume fraction (CVF) were quantified in ImageJ.

### 4.5 Transmission Electron Microscopy

Myocardial blocks (1 mm^3^) were fixed in 2.5% glutaraldehyde (4°C), post‐fixed in 1% osmium tetroxide, dehydrated through graded ethanol, resin‐embedded, ultrathin‐sectioned, and stained (uranyl acetate/lead citrate). Mitochondria, autophagosomes, and autophagolysosomes were evaluated by TEM.

### 4.6 Cell Culture and Treatments

H9c2 cardiomyoblasts (DMEM, Gibco, C11995500BT, USA) were maintained with 10% FBS (Gemini, 900‐108, AUS) and 100 U/mL penicillin/streptomycin (Bdbio, A200‐100, China) at 37°C in 5% CO_2_. Cells were seeded at 1 × 10^5^ cells/mL in six‐well plates. For pharmacological treatment, cells were first pretreated with 1 μM A‐779 (MCE, HY‐P0216, USA), 100 nM PD123319 (MCE, HY‐10259A, USA), or 100 nM Alamandine (MCE, HY‐P3108, USA) for 1 h, then incubated with 80 nM Ang‐(1–7) (MCE, HY‐12403, USA) for 2 h, and finally exposed to isoproterenol (ISO; Solarbio, II0200, China) at 10 μM for 24 h.

### 4.7 Cell Viability

Cells (4000/well, 96‐well) were serum‐starved and treated as above. CCK‐8 (APExBIO, K2268, 10 μL/well, USA) was added for 1 h (37°C, dark), and absorbance was read at 450 nm.

### 4.8 Cytoskeletal Staining

Coverslips were fixed (3.7% paraformaldehyde, 20 min, RT), permeabilized (0.1% Triton X‐100/PBS), and stained with Actin‐Tracker Green (Beyotime, C2201S, 1:100 in 3% BSA/0.1% Triton X‐100/PBS; 200 μL/slide; 45 min, RT, dark). After PBS washes, cell area was quantified (ImageJ 8.0).

### 4.9 Immunofluorescence (Tissue and Cells)

Paraffin heart sections were deparaffinized, antigen‐retrieved, and blocked, then labeled by sequential TSA (YMMED, YM0086Plus‐100 T, China) to detect MasR (FITC) and AT_2_R (Cy3); nuclei were counterstained with DAPI (Coolaber, SL7100, China). H9c2 cells were fixed in 4% paraformaldehyde, permeabilized with 0.2% Triton X‐100 (Coolaber, CT11451, China), and blocked with 3% BSA (Sigma‐Aldrich, B2064) before incubation with primary antibodies (1:500 in 3% BSA/PBS, overnight at 4°C). Fluorescent secondary antibody was added the following day. Images were acquired by fluorescence microscopy, and mean fluorescence intensity was quantified in ImageJ from six random fields per sample across five independent samples per group.

### 4.10 Cellular Thermal Shift Assay (CETSA)

H9c2 cells treated with PBS or Ang‐(1–7) 100 μM were aliquoted and heated for 3 min at 37°C–58°C (eight temperatures), subjected to three freeze–thaw cycles, centrifuged (20 000 g, 4°C), and supernatants immunoblotted to assess MasR thermal stability [49].

### 4.11 Co‐Immunoprecipitation

H9c2 cells were lysed on ice in 1× Lysis/Wash Buffer (ACE Biotechnology Co‐IP kit, BK0004‐02, China) for 35 min; supernatants were clarified (13 000 × g, 15 min) and normalized (700 μg/mL). Lysate (500 μL; 350 μg protein) was incubated at 4°C with rabbit anti‐MasR (Beyotime A7016; IgG as negative control). rProtein A/G magnetic beads (20 μL; pre‐washed) were added for 2 h (RT). Beads were washed (3× Lysis/Wash Buffer), then eluted with 1× SDS sample buffer (100°C, 10 min) for immunoblotting.

### 4.12 TUNEL

H9c2 cells were fixed (4% paraformaldehyde, 30 min), permeabilized (0.3% Triton X‐100/PBS, 5 min), and incubated with TUNEL mix (Beyotime; TdT:fluorescein = 1:9; 60 min, 37°C, dark). Negative controls omitted TdT; positive controls were pretreated with DNase I (1 U/μL). Nuclei were counterstained with DAPI (5 μg/mL, 10 min, 37°C). ≥ 100 cells from five fields/group were quantified by two independent investigators.

### 4.13 Real‐Time PCR

Primers were designed/validated from NCBI sequences (Table S1). H9c2 cells were lysed directly in TRNzol (Tiangen, DP424, China); cardiac tissue was homogenized in TRNzol on ice. Phase separation (chloroform), RNA precipitation, ethanol wash (75%), and resuspension followed manufacturer instructions. RNA quantity/purity was assessed spectrophotometrically (BioTek Synergy MX). cDNA was synthesized from 1 μg RNA (Tiangen, KR118, China). RT‐qPCR was performed with SYBR Green master mix (Tiangen, FP205, China) on a real‐time system; GAPDH served as an internal control. Relative expression was calculated by the 2^−ΔΔCt^ method.

### 4.14 Molecular Docking

Receptor structures (PDB) were curated (waters/ligands removed, energy‐minimized). Ang‐(1–7) was prepared and docked; the best poses and ΔG values were recorded and visualized to infer binding/heterodimer interfaces.

### 4.15 Western Blotting

Proteins were isolated from H9c2 cells and mouse heart tissues, and lysed in ice‐cold RIPA lysis buffer (Solarbio, R0010, China) containing protease inhibitor cocktail (1 mmol/L) and phenylmethylsulfonyl fluoride (PMSF; 1 mmol/L). Protein content was determined using the BCA protein assay kit (Coolaber, SK1070, China). Proteins were separated by 12.5% SDS‐polyacrylamide gel electrophoresis and transferred onto a 0.2 μm NC membrane (Cytiva, A3085788, USA). The membrane was blocked with 5% skim milk powder (Coolaber, CN7861, China) dissolved in TBST buffer at room temperature for 1 h. Subsequently, the membrane was incubated overnight at 4°C with the following primary antibodies: ANP (Affinity, DF6497, China), BNP (Affinity, DF6902, China), β‐MHC (ABclonal, A22140, China), MasR (ThermoFisher scientific, MA557066, USA), AT_2_R (ABclonal, A3654, China), Beclin1 (proteintech, 11306‐1‐AP, USA), p62 (ABclonal, A19700, China), LC3B (ABclonal, Cat No. A19665), Bax (abcam, ab32503, UK), Cleaved‐caspase 3 (Cell Signaling Technology, 9961, USA), Bcl2 (proteintech, 26593‐1‐AP, USA), and GAPDH (Nature Biosciences, MA0003, China). After washing the membrane three times with TBST on the second day, incubate it with IgG (H + L) fluorescent secondary antibody (Jackson, 711‐655‐152, USA) at room temperature for 2 h. Then, use an imaging system (CLX‐3464, LI‐COR) to capture images, and analyze the grayscale values of the bands using ImageJ software.

### 4.16 Statistical Analysis

Data are presented as mean ± SD. One‐way ANOVA was applied for multi‐group comparisons (version 8.0; GraphPad Software Inc., SanDiego, CA, USA). *p* < 0.05 was considered significant. Biological replicates were ≥ 5 unless indicated.

## Author Contributions

Xiaomei Wang designed the study, performed the experiments, and drafted the initial manuscript. Xiaoqian Wang, Fei Guo, and Yu Guo jointly carried out portions of the experimental work and data analysis. Siyao Fan was responsible for proofreading the manuscript and carrying out portions of the experimental work. Lan Hong contributed to the study conception and design. Honghua Jin was responsible for the study conception and design, manuscript discussion, and funding acquisition. All authors reviewed and approved the final version of the manuscript and affirm the accuracy and reliability of the data.

## Funding

This work was supported by the National Natural Science Foundation of China (NSFC) (grant number 81860077).

## Conflicts of Interest

The authors declare no conflicts of interest.

## Supporting information


**Figure S1:** Immunofluorescence images of H9C2 cells stained with F‐actin (green) and DAPI (blue), showing changes in cell surface area across different treatment groups (scale bar = 10 μm). The data is expressed as an mean ± standard deviation (SD) (*n* = 6). ***p < 0.001 versus Ctrl; ^###^ *p* < 0.001 versus ISO; ^&&&^ *p* < 0.001, ^&^ *p* < 0.05 versus Ang‐(1–7) + ISO; ns versus ISO.
**Figure S2:** Effects of Ang‐(1–7) on the expression of hypertrophy‐related markers in H9c2 cardiomyocytes. (A) Western blot analysis of hypertrophy‐related marker proteins (β‐MHC, BNP, ANP) in H9c2 cardiomyocytes. GAPDH was used as the loading control. (B) Quantitative analysis of the relative protein expression levels of the above hypertrophy‐related markers, normalized to GAPDH. The data is expressed as an mean ± standard deviation (SD) (*n* = 3). *p < 0.05 versus Ctrl; ^###^ *p* < 0.001 versus ISO; ^&&^ *p* < 0.01, ^&^ *p* < 0.05 versus Ang‐(1–7) + ISO; ns versus ISO.
**Figure S3:** Analyzes MasR expression and relative thermal stability in cardiomyocytes at different temperatures (37°C–58°C).
**Figure S4:** Regulatory Effect of Ang‐(1–7) on the Protein Expression of AT_2_R and MasR in H9c2 Cardiomyocytes. (A) Western blot analysis of the protein expression levels of angiotensin II type 2 receptor (AT_2_R) and Mas receptor (MasR) in H9c2 cardiomyocytes. GAPDH was used as the loading to GAPDH. The data is expressed as an mean ± standard deviation (SD) (*n* = 3). ***p < 0.001 versus Ctrl; ^###^ *p* < 0.001 versus ISO; ^&&&^ *p* < 0.001, ^&&^ *p* < 0.01, ^&^ *p* < 0.05 versus Ang‐(1–7) + ISO; ns versus ISO.
**Figure S5:** Effects of Different Treatments on p62 Localization, Autophagy, and Apoptosis‐related Indicators in H9c2 Cardiomyocytes. (A) Immunofluorescence staining shows the subcellular localization of p62 protein (green fluorescence) in H9c2 cardiomyocytes, with DAPI staining marking the nuclei (blue). The Merge image is the overlay of both channels. (B) Autophagy‐related indicators: area of p62‐positive regions; LC3II/LC3I ratio; relative expression level of Beclin1; relative expression level of Bax. (C) Apoptosis‐related indicators: cell apoptosis index; relative expression level of Bcl‐2; Bax/Bcl‐2 ratio; relative expression level of cleaved caspase‐3. The data is expressed as an mean ± standard deviation (SD) (*n* = 3–6). ^##^ *p* < 0.01, ^#^ *p* < 0.01 versus ISO; ^&&&^ *p* < 0.001, ^&&^ *p* < 0.01, &*p* < 0.05 versus Ang‐(1–7) + ISO.
**Figure S6:** Immunofluorescence localization of MasR and AT_2_R in iPSC‐derived cardiomyocytes. Representative immunofluorescence images showing the expression and subcellular localization of the MasR and AT_2_R in induced pluripotent stem cell (iPSC)‐derived cardiomyocytes. Left panel: Green fluorescence signal indicates MasR expression. Middle panel: Red fluorescence signal indicates AT_2_R expression. Right panel: Merged image of MasR and AT_2_Rsignals, with the inset providing a magnified view highlighting the detailed spatial distribution of both receptors. Scale bars: 90 μm (*n* = 3).
**Figure S7:** Regulatory effects of Ang‐(1–7) and Alamandine on ISO‐induced hypertrophy in H9c2 cardiomyocytes. (A) Representative immunofluorescence images of H9c2 cells (red fluorescence outlines cell borders, blue: DAPI nuclear staining). Scale bars: 90 μm. (B) Western blot analysis of hypertrophy markers (β‐MHC, BNP, ANP). GAPDH was used as the loading control. (C) Quantitative analysis of the relative expression levels of hypertrophy markers (ANP, BNP, β‐MHC) normalized to GAPDH. The data is expressed as an mean ± standard deviation (SD) (*n* = 3). ***p < 0.001, **p < 0.01 versus Ctrl; ^###^ *p* < 0.001, ^##^ *p* < 0.01 versus ISO; ^&^ *p* < 0.05 versus ISO; ns versus Alamandine + ISO.
**Figure S8:** Regulation of MasR and AT_2_R expression by Ang‐(1–7) and Alamandine in ISO‐induced H9c2 cardiomyocytes. (A) Western blot analysis of MasR and AT_2_R protein levels in H9c2 cardiomyocytes. GAPDH was used as the loading control. (B) Quantitative results: relative protein expression of AT_2_R and MasR normalized to GAPDH; relative mRNA expression of AT_2_R and MasR compared to the Ctrl group. The data is expressed as an mean ± standard deviation (SD) (*n* = 3). ***p < 0.001, **p < 0.01, *p < 0.05 versus Ctrl; ^###^ *p* < 0.001, ^##^ *p* < 0.01, ^#^ *p* < 0.05 versus ISO; ^&&&^ *p* < 0.001, ^&&^ *p* < 0.01, ^&^ *p* < 0.05 versus ISO; ns versus Alamandine + ISO.
**Figure S9:** Effects of MasR and AT_2_R antagonists on the expression of hypertrophy markers in ISO‐induced H9c2 cardiomyocytes. (A) Western blot analysis of hypertrophy marker proteins (β‐MHC, BNP, ANP). GAPDH was used as the loading control. Treatment groups include: control (Ctrl), isoproterenol‐induced hypertrophy (ISO), MasR antagonist A779 + ISO, and AT_2_R antagonist PD123319 + ISO. Molecular weights (kDa) of the corresponding proteins are indicated on the right. (B) Relative protein expression levels of ANP, BNP, and β‐MHC normalized to GAPDH. (C) Relative mRNA expression levels of ANP, BNP, and β‐MHC compared to the Ctrl group. The data is expressed as an mean ± standard deviation (SD) (*n* = 3). ***p < 0.001 versus Ctrl; ns versus ISO.
**Table S1:** Primer sequences for real‐time PCR.

## Data Availability

The data and materials used to support the findings of this study are available from the corresponding authors upon reasonable request.
