# Supplementary material for: Angiotensin‐(1–7) Alleviates Isoproterenol‐Induced Cardiac Hypertrophy by Suppressing Autophagy and Apoptosis Through the Synergistic Action of Mas Receptor and Angiotensin II Type 2 Receptor
Source: Acta Physiol (Oxf). 2026 Mar 26;242(4):e70200. doi: 10.1111/apha.70200 (PMC13021233; doi:10.1111/apha.70200)
Supplement: Supplementary file 1 — Figure S1: Immunofluorescence images of H9C2 cells stained with F‐actin (green) and DAPI (blue), showing changes in cell surface area across different treatment groups (scale bar = 10 μm). The data is expressed as an mean ± standard deviation (SD) (n = 6). ***p < 0.001 versus Ctrl; ### p < 0.001 versus ISO; &&& p < 0.001, & p < 0.05 versus Ang‐(1–7) + ISO; ns versus ISO. Figure S2: Effects of Ang‐(1–7) on the expression of hypertrophy‐related markers in H9c2 cardiomyocytes. (A) Western blot analysis of hypertrophy‐related marker proteins (β‐MHC, BNP, ANP) in H9c2 cardiomyocytes. GAPDH was used as the loading control. (B) Quantitative analysis of the relative protein expression levels of the above hypertrophy‐related markers, normalized to GAPDH. The data is expressed as an mean ± standard deviation (SD) (n = 3). *p < 0.05 versus Ctrl; ### p < 0.001 versus ISO; && p < 0.01, & p < 0.05 versus Ang‐(1–7) + ISO; ns versus ISO. Figure S3: Analyzes MasR expression and relative thermal stability in cardiomyocytes at different temperatures (37°C–58°C). Figure S4: Regulatory Effect of Ang‐(1–7) on the Protein Expression of AT2R and MasR in H9c2 Cardiomyocytes. (A) Western blot analysis of the protein expression levels of angiotensin II type 2 receptor (AT2R) and Mas receptor (MasR) in H9c2 cardiomyocytes. GAPDH was used as the loading to GAPDH. The data is expressed as an mean ± standard deviation (SD) (n = 3). ***p < 0.001 versus Ctrl; ### p < 0.001 versus ISO; &&& p < 0.001, && p < 0.01, & p < 0.05 versus Ang‐(1–7) + ISO; ns versus ISO. Figure S5: Effects of Different Treatments on p62 Localization, Autophagy, and Apoptosis‐related Indicators in H9c2 Cardiomyocytes. (A) Immunofluorescence staining shows the subcellular localization of p62 protein (green fluorescence) in H9c2 cardiomyocytes, with DAPI staining marking the nuclei (blue). The Merge image is the overlay of both channels. (B) Autophagy‐related indicators: area of p62‐positive regions; LC3II/LC3I ratio; relative [file APHA-242-e70200-s001.docx]

**Angiotensin-(1-7) alleviates isoproterenol-induced cardiac hypertrophy by suppressing autophagy and apoptosis through the synergistic action of Mas receptor and angiotensin II type 2 receptor**

Xiaomei Wang^1^ Fei Guo^1^ Xiaoqian Wang^1^ Yu Guo^1^ Siyao Fan^2^ Lan Hong^2^* Honghua Jin^3^*

^1^College of Pharmacy, Yanbian University, Yanji, 133002, China.

^2^Department of physiology and pathophysiology, College of medicine, Yanbian University, Yanji 133002, China.

^3^Department of Pharmacy, Yanbian University Hospital, Yanbian University, Yanji 133000, China.

***Corresponding author:**

Lan Hong, PhD

Department of physiology and pathophysiology, College of medicine, Yanbian University, Jilin China.

No. 977, Gongyuan Road, Yanji ,City

E-mail: [honglan@ybu.edu.cn](mailto:honglan@ybu.edu.cn)

Honghua Jin, PhD

Department of Pharmacy, Yanbian University Hospital, Yanbian University,

Jilin China.

No.1327, Juzi Street, Yanji, City

E-mail: honghuajin@ybu.edu.cn

**SUPPLEMENTARY TEXT**

**Sequences Table 1**

Primer sequences for real-time PCR

| Target  gene | Forward | Reverse |
| --- | --- | --- |
| ANP | GGGAAGTCAACCCGTCTCAG | CAATCCTACCCCCGAAGCAG |
| BNP | TTCCGGATCCAGGAGAGACTT | CCTAAAACAACCTCAGCCCGT |
| β-MHC | TTTGATGTGCTGGGCTTCAC | TGACATACTCGTTGCCCACT |
| MasR | GCCCACAGAGGAGAGATTCG | AGTAACATGGGCAGCATCGG |
| AT_2_R | GGCAGATAAGCATTTGGAAGC | CCACAGCCAGATTGAAGATG |

**Supplementary Figure S1**

(A) Immunofluorescence images of H9C2 cells stained with F-actin (green) and DAPI (blue), showing changes in cell surface area across different treatment groups (scale bar = 10 μm). The data is expressed as an mean ± standard deviation (SD) (n=6). A+I, Ang-(1-7)+ISO; AntM+A+I, A-779+ Ang-(1-7)+ISO; AntA+A+I, PD123319+Ang-(1-7)+IS; AntM+AntA+A+I, A-779+PD123319+Ang-(1-7)+ISO. ***P<0.001 vs Ctrl; ###P<0.001 vs ISO; &&&P<0.001, &P<0.05 vs Ang-(1–7)+ISO; ns vs ISO.

**Supplementary Figure S2**

**Effects of Ang-(1–7) on the expression of hypertrophy-related markers in H9c2 cardiomyocytes.** (A) Western blot analysis of hypertrophy-related marker proteins (β‑MHC, BNP, ANP) in H9c2 cardiomyocytes. GAPDH was used as the loading control. (B) Quantitative analysis of the relative protein expression levels of the above hypertrophy-related markers, normalized to GAPDH. The data is expressed as an mean ± standard deviation (SD) (n=3). A+I, Ang-(1-7)+ISO; AntM+A+I, A-779+ Ang-(1-7)+ISO; AntA+A+I, PD123319+Ang-(1-7)+IS; AntM+AntA+A+I, A-779+PD123319+Ang-(1-7)+ISO. *P<0.05 vs Ctrl; ###P<0.001 vs ISO; &&P<0.01, &P<0.05 vs Ang-(1–7)+ISO; ns vs ISO.


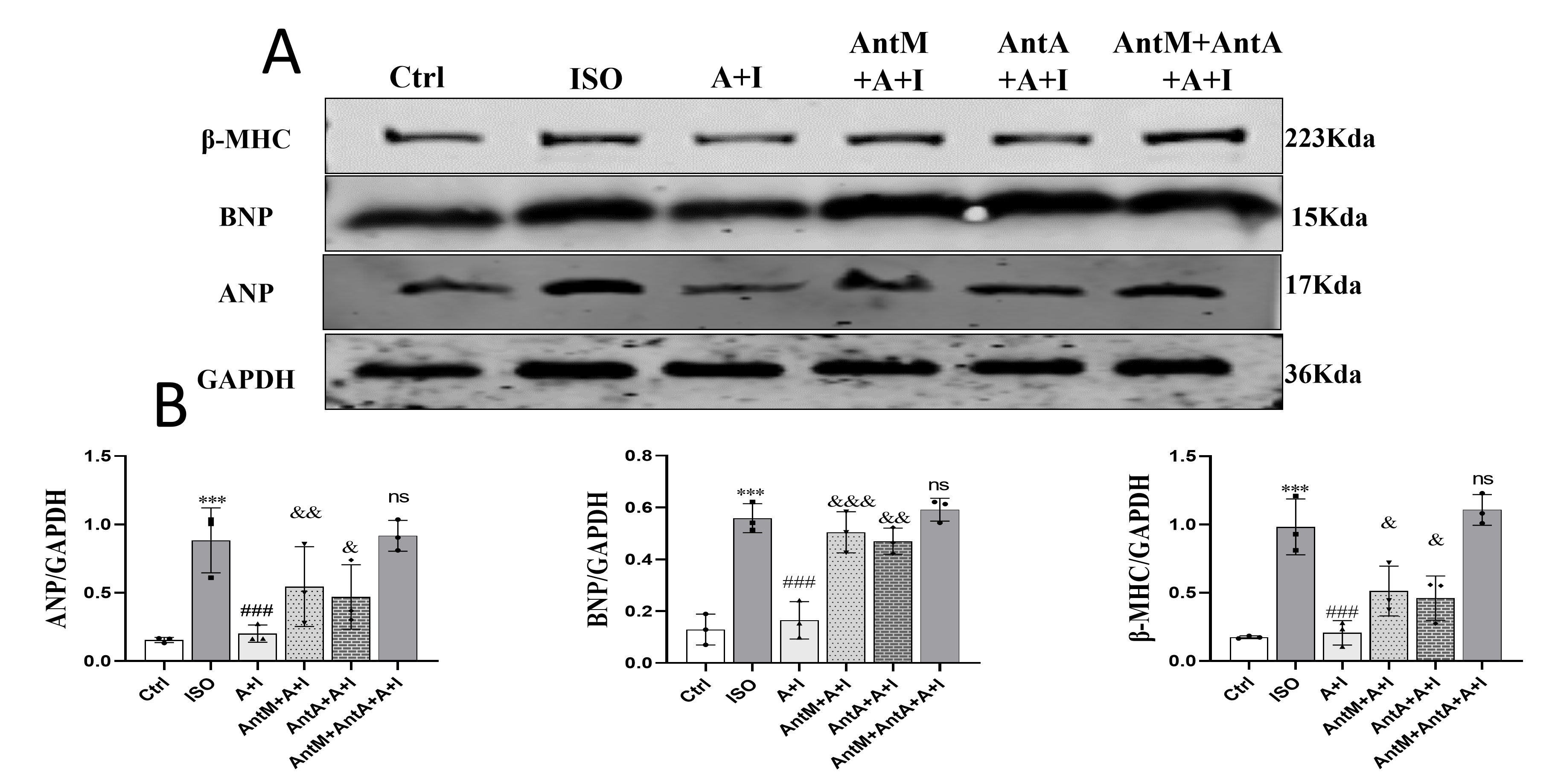


**Supplementary Figure S3**

Analyzes MasR expression and relative thermal stability in cardiomyocytes at different temperatures (37°C–58°C)


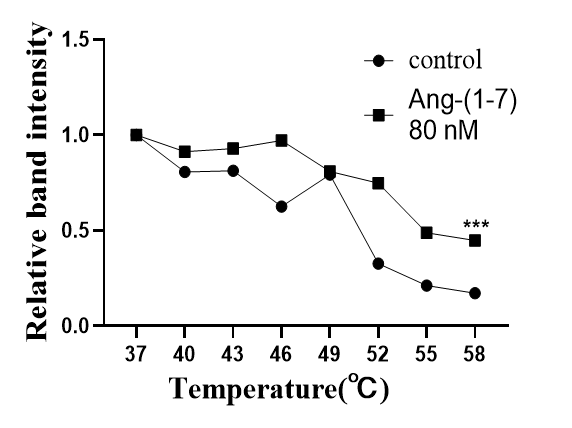


**Supplementary Figure S4**

**Regulatory Effect of Ang-(1–7) on the Protein Expression of AT₂R and MasR in H9c2 Cardiomyocytes.** (A) Western blot analysis of the protein expression levels of angiotensin II type 2 receptor (AT₂R) and Mas receptor (MasR) in H9c2 cardiomyocytes. GAPDH was used as the loading to GAPDH). The data is expressed as an mean ± standard deviation (SD) (n=3). A+I ,Ang-(1-7)+ISO; AntM+A+I, A-779+ Ang-(1-7)+ISO; AntA+A+I, PD123319+Ang-(1-7)+ISO; AntM+AntA+A+I, A-779+PD123319+Ang-(1-7)+ISO. ***P<0.001 vs Ctrl; ###P<0.001 vs ISO; &&&P<0.001, &&P<0.01, &P<0.05 vs Ang-(1-7) +ISO.ns vs ISO


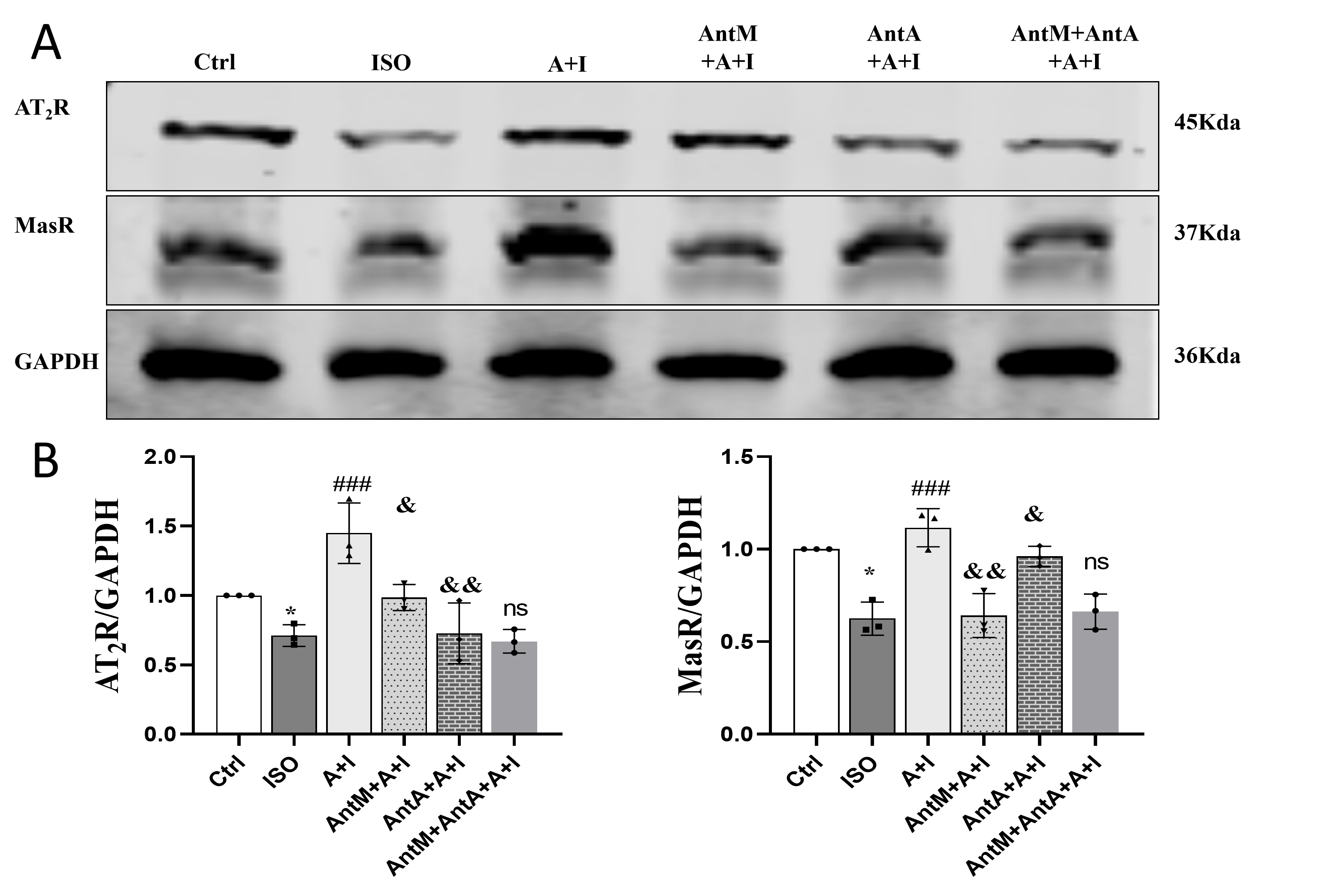


**Supplementary Figure S5**


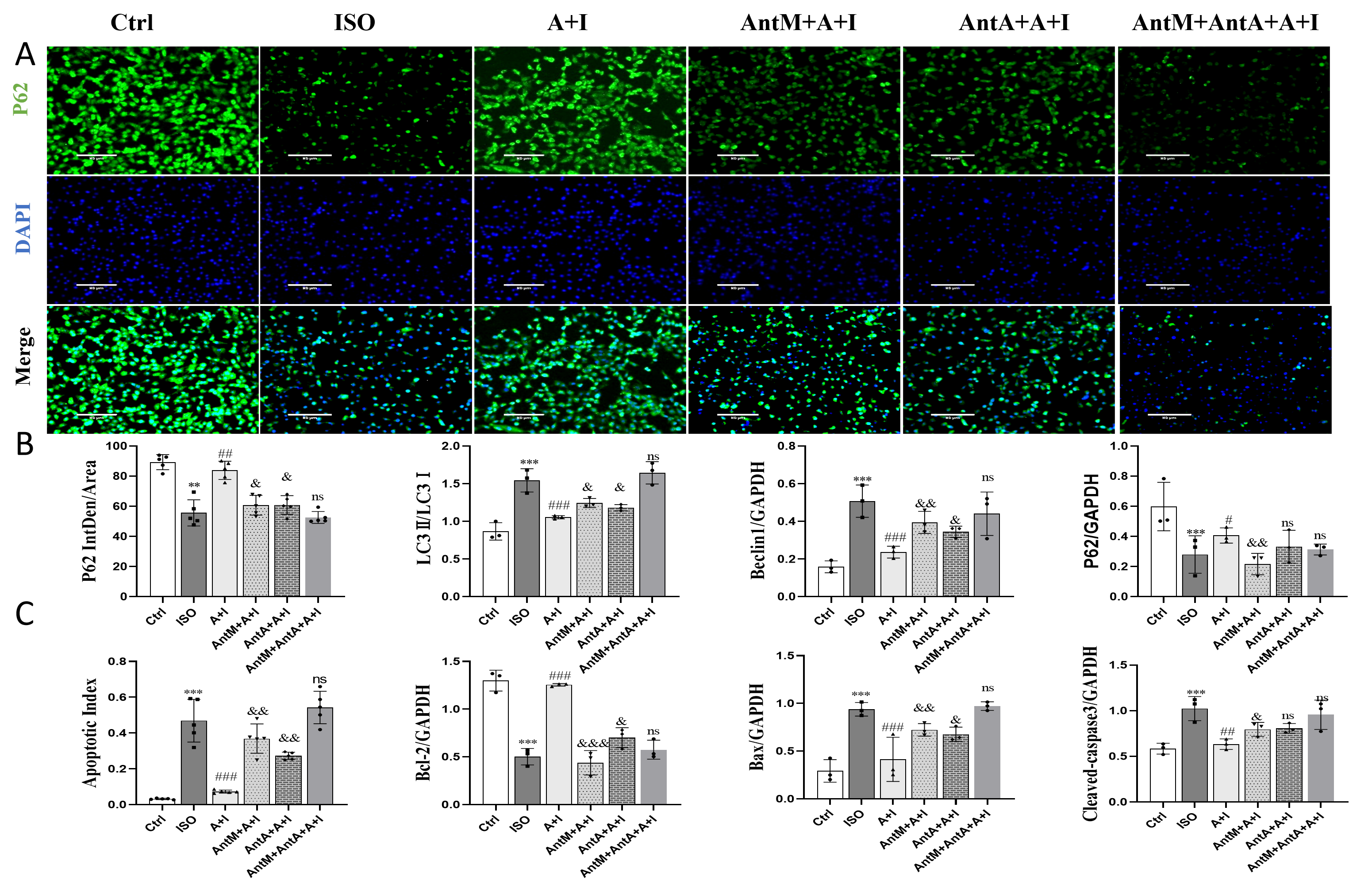
****Effects of Different Treatments on p62 Localization, Autophagy, and Apoptosis-related Indicators in H9c2 Cardiomyocytes.**** (A) Immunofluorescence staining shows the subcellular localization of p62 protein (green fluorescence) in H9c2 cardiomyocytes, with DAPI staining marking the nuclei (blue). The Merge image is the overlay of both channels. (B) Autophagy-related indicators: area of p62-positive regions; LC3II/LC3I ratio; relative expression level of Beclin1; relative expression level of Bax. (C) Apoptosis-related indicators: cell apoptosis index; relative expression level of Bcl-2; Bax/Bcl-2 ratio; relative expression level of cleaved caspase-3. The data is expressed as an mean ± standard deviation (SD) (n=3-6). A+I, Ang-(1-7)+ISO; AntM+A+I, A-779+ Ang-(1-7)+ISO; AntA+A+I, PD123319+Ang-(1-7)+IS0; AntM+AntA+A+I, A-779+PD123319+Ang-(1-7)+ISO. ***P< 0.001, **P<0.01 vs Ctrl; ###P< 0.001, ##P<0.01 ,#P<0.01vs ISO; &&&P<0.001, &&P<0.01, &P<0.05 vs Ang-(1-7) +ISO.

**Supplementary Figure S6**

**Immunofluorescence localization of MasR and AT₂R in iPSC-derived cardiomyocytes.**

Representative immunofluorescence images showing the expression and subcellular localization of the Mas receptor (MasR) and angiotensin II type 2 receptor (AT₂R) in induced pluripotent stem cell (iPSC)-derived cardiomyocytes. Left panel: Green fluorescence signal indicates MasR expression. Middle panel: Red fluorescence signal indicates AT₂R expression. Right panel: Merged image of MasR and AT₂R signals, with the inset providing a magnified view highlighting the detailed spatial distribution of both receptors. Scale bars: 90 μm(n=3).

**
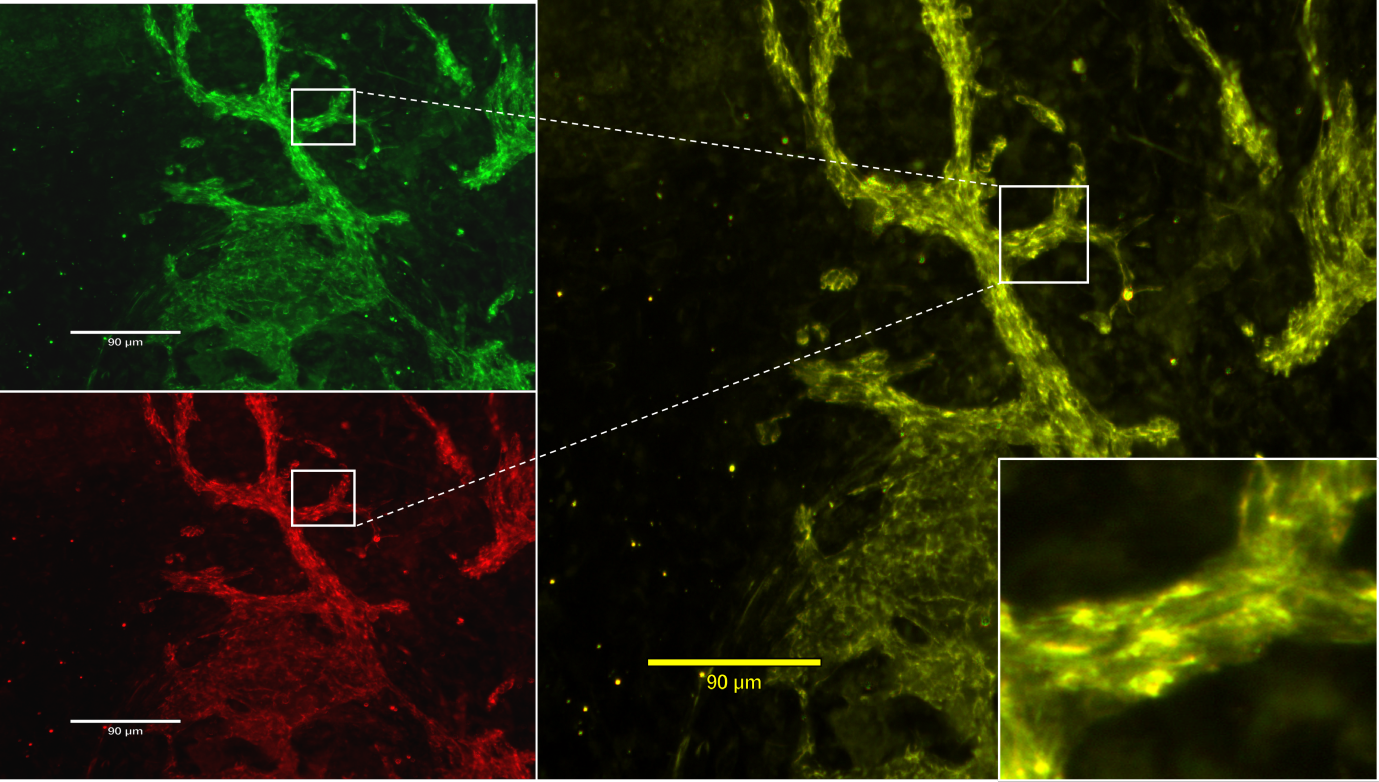
**

**Supplementary Figure S7**

**
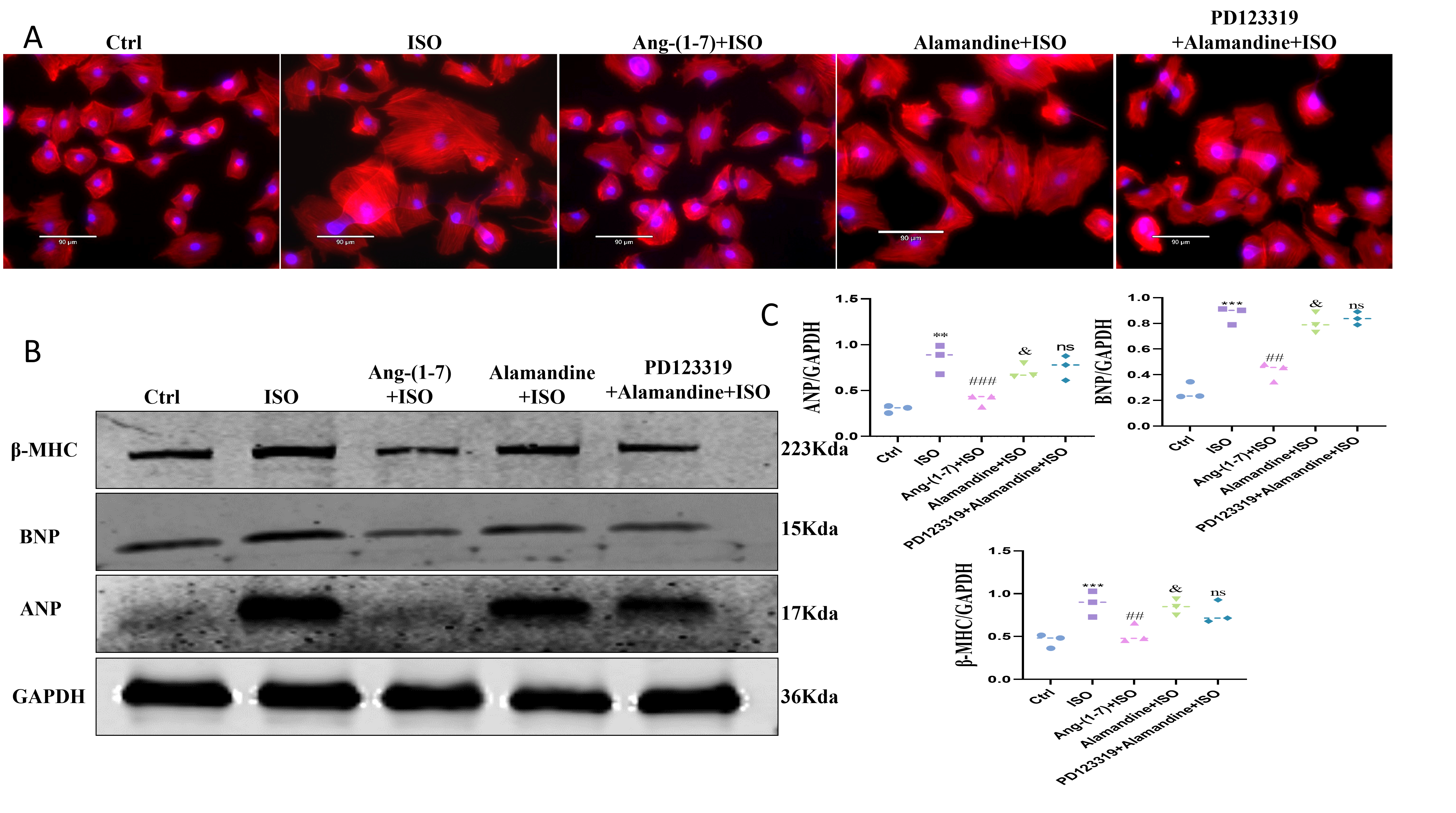
Regulatory effects of Ang-(1–7) and Alamandine on ISO‑induced hypertrophy in H9c2 cardiomyocytes.** (A) Representative immunofluorescence images of H9c2 cells (red fluorescence outlines cell borders, blue: DAPI nuclear staining). Scale bars: 90 μm. (B) Western blot analysis of hypertrophy markers (β‑MHC, BNP, ANP). GAPDH was used as the loading control. (C) Quantitative analysis of the relative expression levels of hypertrophy markers (ANP, BNP, β‑MHC) normalized to GAPDH. The data is expressed as an mean ± standard deviation (SD) (n=3). ***P< 0.001, **P<0.01 vs Ctrl; ###P< 0.001, ##P<0.01 vs ISO; &P<0.05 vs ISO; ns vs Alamandine+ISO.

**Supplementary Figure S8**

**Regulation of MasR and AT₂R expression by Ang-(1–7) and Alamandine in ISO‑induced H9c2 cardiomyocytes.** (A) Western blot analysis of Mas receptor (MasR) and angiotensin II type 2 receptor (AT₂R) protein levels in H9c2 cardiomyocytes. GAPDH was used as the loading control. (B) Quantitative results: relative protein expression of AT₂R and MasR normalized to GAPDH; relative mRNA expression of AT₂R and MasR compared to the Ctrl group. The data is expressed as an mean±standard deviation (SD) (n=3). ***P< 0.001, **P<0.01 , *P<0.05vs Ctrl; ###P< 0.001, ##P<0.01 , #P<0.05 vs ISO; &&&P< 0.001, &&P<0.01 , &P<0.05 vs ISO; ns vs Alamandine+ISO.

**
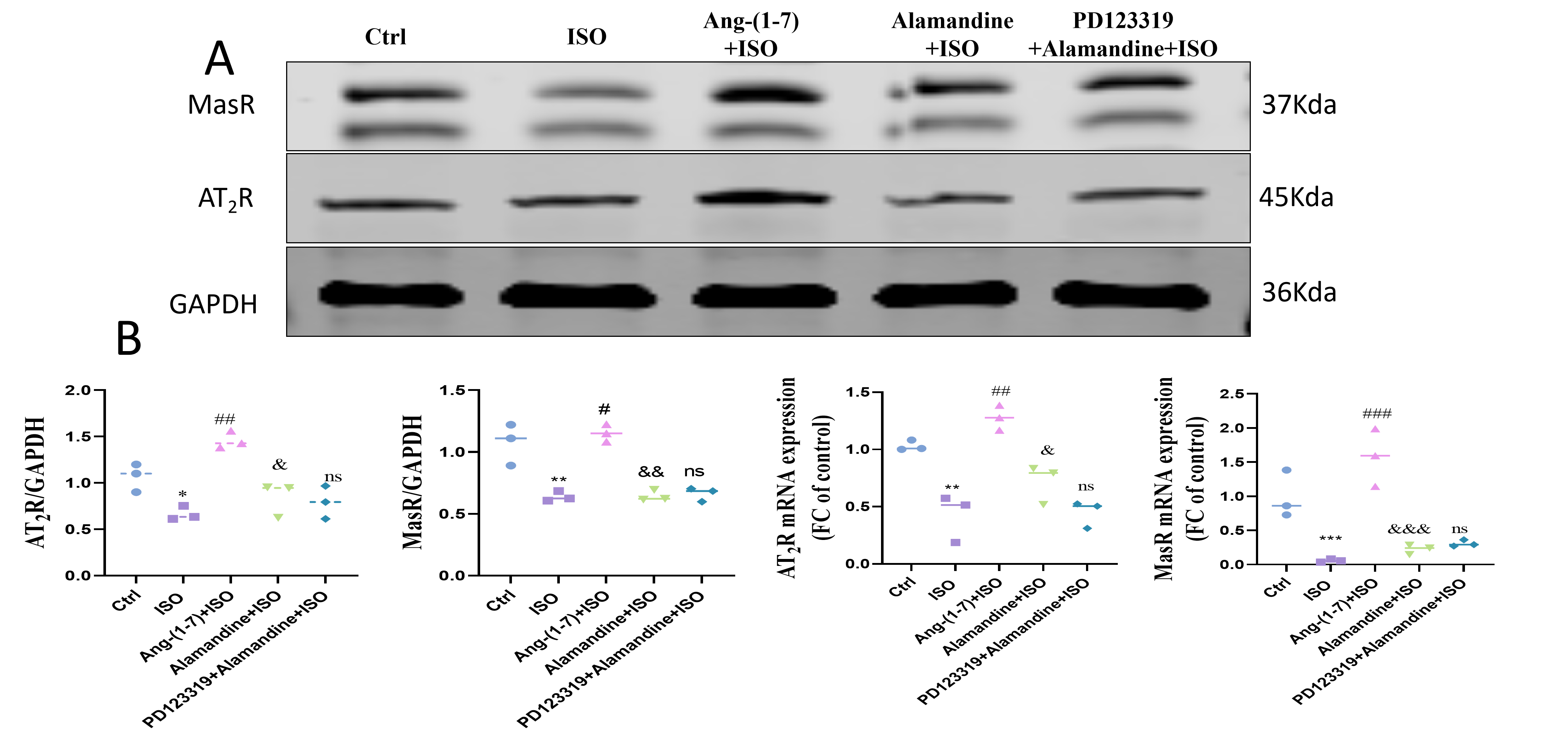
**

**Supplementary Figure S9**

**Effects of MasR and AT₂R antagonists on the expression of hypertrophy markers in ISO‑induced H9c2 cardiomyocytes.** (A) Western blot analysis of hypertrophy marker proteins (β‑MHC, BNP, ANP). GAPDH was used as the loading control. Treatment groups include: control (Ctrl), isoproterenol‑induced hypertrophy (ISO), MasR antagonist A779 + ISO, and AT₂R antagonist PD123319 + ISO. Molecular weights (kDa) of the corresponding proteins are indicated on the right. (B) Relative protein expression levels of ANP, BNP, and β‑MHC normalized to GAPDH. (C) Relative mRNA expression levels of ANP, BNP, and β‑MHC compared to the Ctrl group. The data is expressed as an mean ± standard deviation (SD) (n=3). ***P< 0.001 vs Ctrl; ns vs ISO.

**
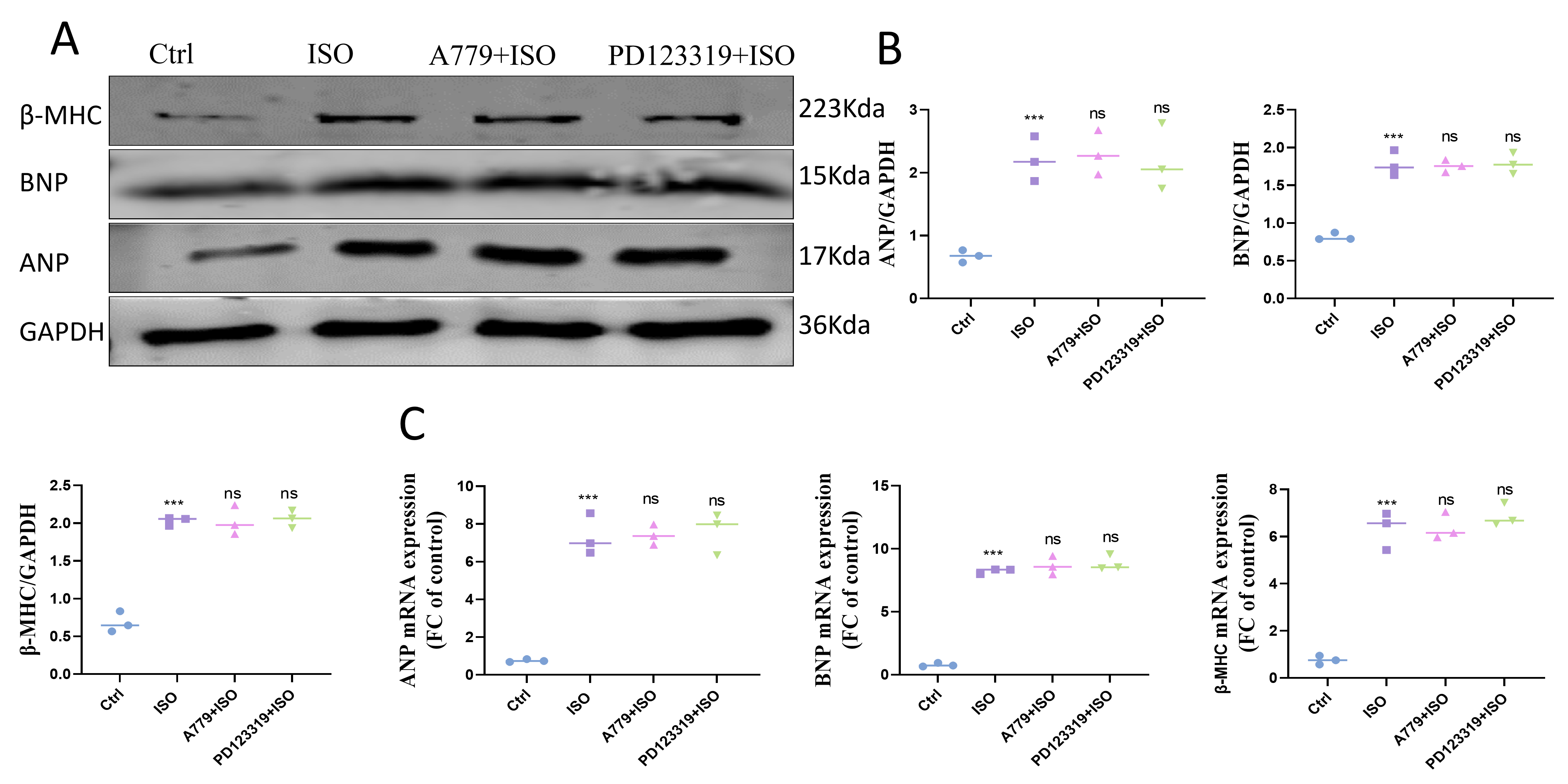
**

**Materials and Methods**

1. **Hemolysis assay**

H&E : For myocardial cell cross-sectional area analysis, At least 5 non-overlapping fields of view (×400 magnification) were randomly selected per section in the free wall region of the left ventricle. Within each field of view, approximately 20 cross-sectioned myocardial cells exhibiting clear, intact cell membranes and nuclei with a near-circular shape were measured. Ultimately, with n=5 animals per group, a cumulative total of no fewer than 450 myocardial cells were analyzed.

Masson: Six random fields of view (×200 magnification) were selected from the left ventricular myocardium of each animal heart section, excluding vascular and epicardial peripheral areas. Each group analyzed n=5 animals.

1. **Transmission electron microscopy**

Sample size and procedure for image analysis: For each rat, 3 independent regions of the left ventricular free wall were harvested to prepare 1 mm³ myocardial tissue blocks (at least 3 biological replicates per rat). After embedding each tissue block, 5 ultrathin sections were prepared as technical replicates. For each section, 6 non-overlapping fields of view were randomly selected using unified parameters (12000×magnification for mitochondrial observation; 20000× magnification for autophagic structure observation), and at least 5 intact cardiomyocytes per field of view were analyzed. Blinded quantification (by personnel unaware of sample grouping) of the average cross-sectional area of mitochondria and mitochondrial cristae disruption ratio was conducted using ImageJ. The results from 4 tissue blocks, 5 sections, and all fields of view per rat were averaged to serve as one biological replicate, and structural differences between groups were finally statistically evaluated.

1. **Human induced pluripotent stem cells (hiPSCs, CELLAPY, Beijing, China) were induced to differentiate into cardiomyocytes.**

The CardioEasy® Human Cardiomyocyte Differentiation Kit (CELLAPY, CA2004500, Beijing, China) was used to prepare the culture medium. Differentiation was initiated when hiPSC cells reached 90% confluence. Following PBS washing, sequentially add Medium I and incubate at 37°C (replace with Medium II after 48 hours, then with Medium III after a further 48 hours). Thereafter, replace with Medium III every 48 hours. Observe cardiomyocyte differentiation daily until beating cardiomyocytes are visible under microscopy, indicating successful differentiation.

Following differentiation, wash the cardiomyocytes with PBS and add CardioEasy® Human Cardiomyocyte Digestive Solution I (CELLAPY, CA2011100, Beijing, China). Incubate for 5–10 minutes, then discard the solution. Add CardioEasy® Human Cardiomyocyte Digestive Solution II (CELLAPY, CA2012100, Beijing, China) for 15–20 minutes. Following digestion termination, centrifuge at 200g for 5 minutes. Resuspend cells and plate into six-well plates pre-coated with PSCeasy Human Pluripotent Stem Cell Seeding Working Solution (CELLAPY, CA3003100, Beijing, China). After stabilisation, proceed with staining procedures as described in section 4.9.

1. **Cytoskeletal staining**

Sample size for image analysis: At least 3 independent cell culture wells (biological replicates) were set up for each treatment group, with 1 stained coverslip placed in each well. At least 6 non-overlapping fields of view were randomly selected from each coverslip, and at least 20 intact stained cells were analyzed per field of view. Blinded analysis was performed by personnel unaware of sample grouping; the cell area results from all fields of view in each well were averaged as the technical replicate of that well, and inter-group statistics were finally conducted using the biological replicate data of each treatment group.

**Original image Figure-2F**


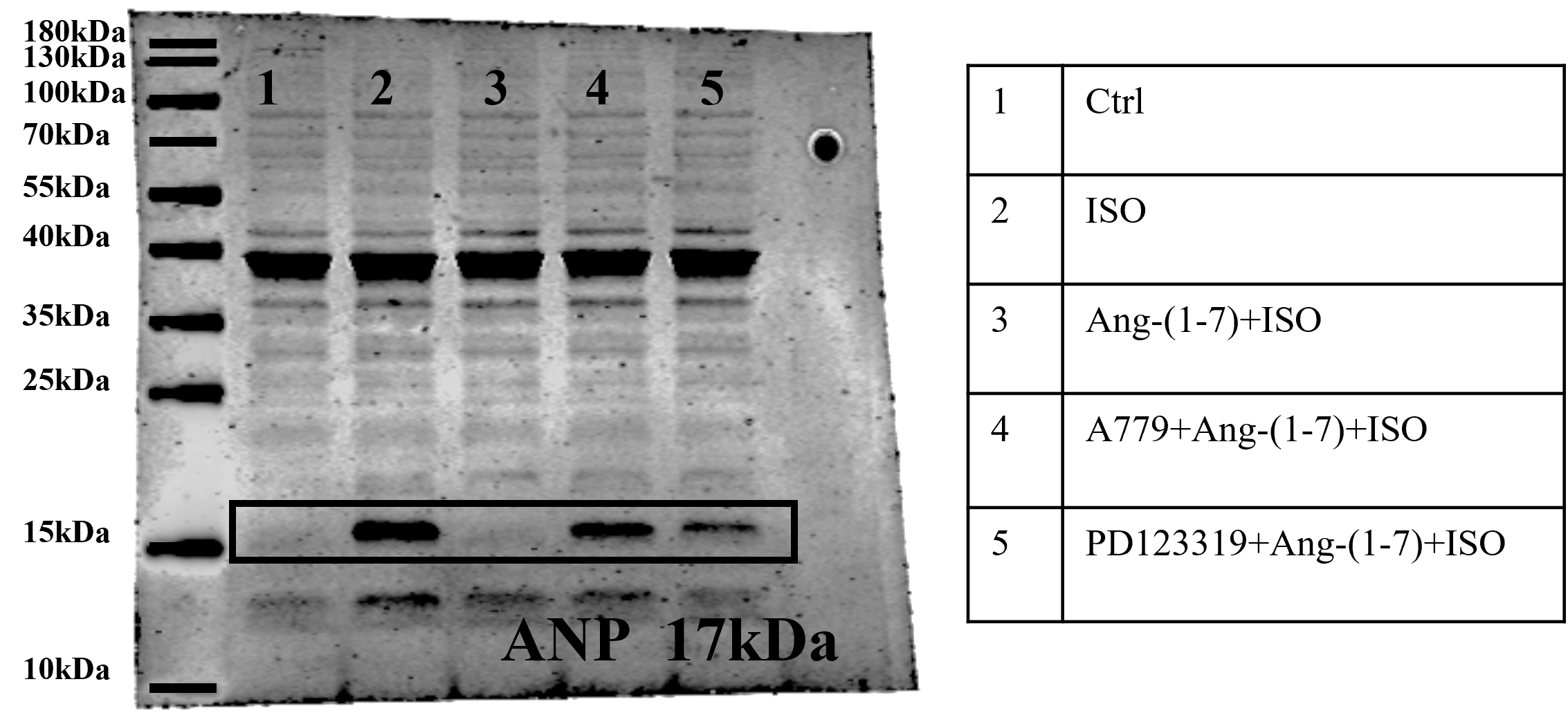

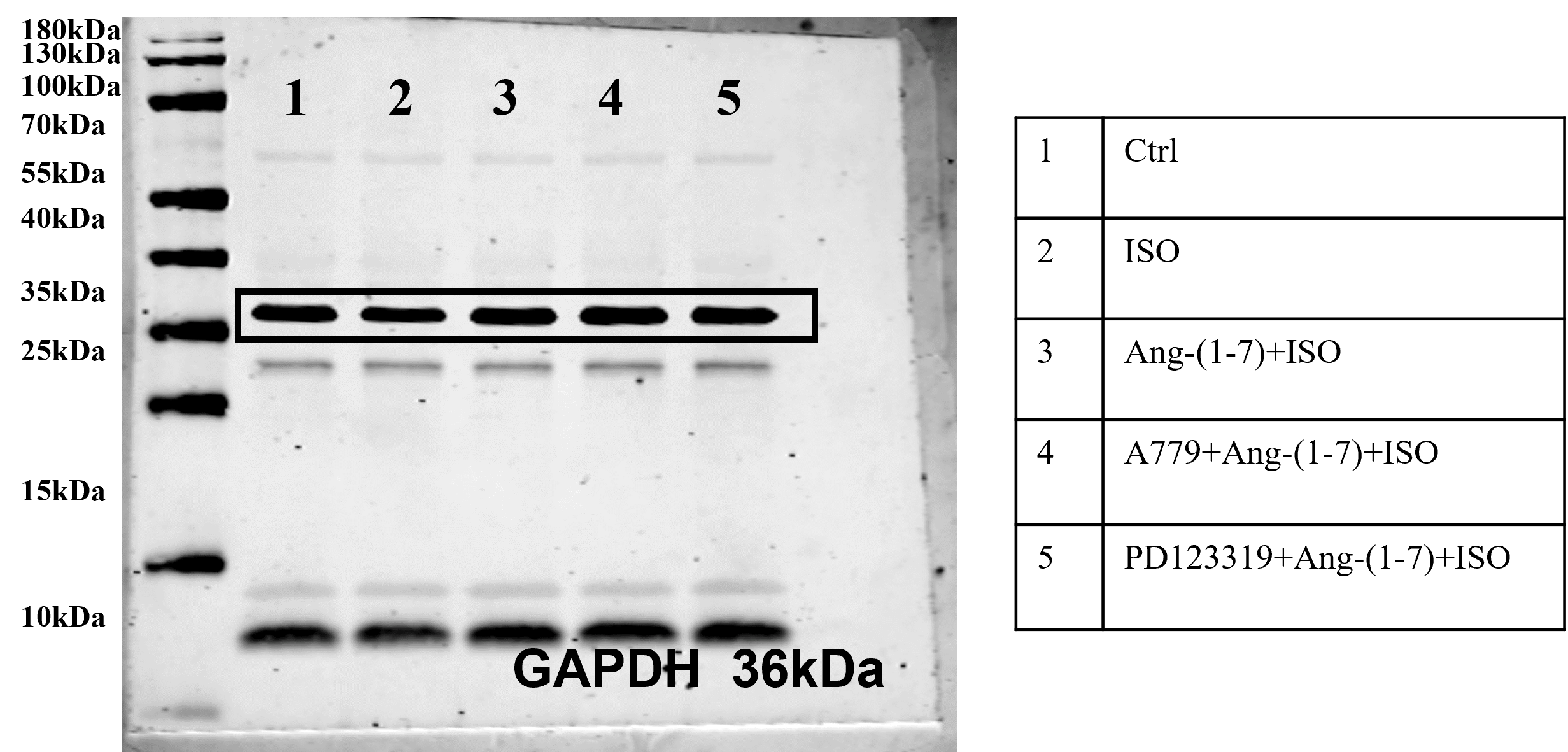


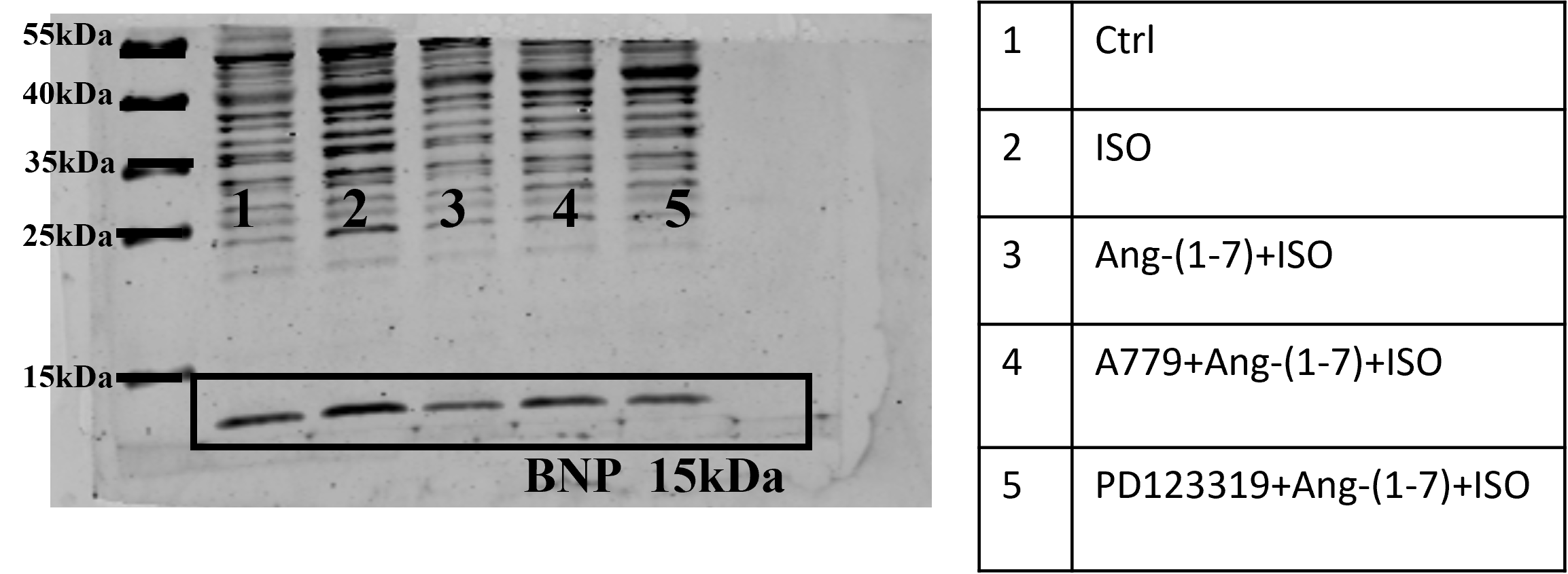

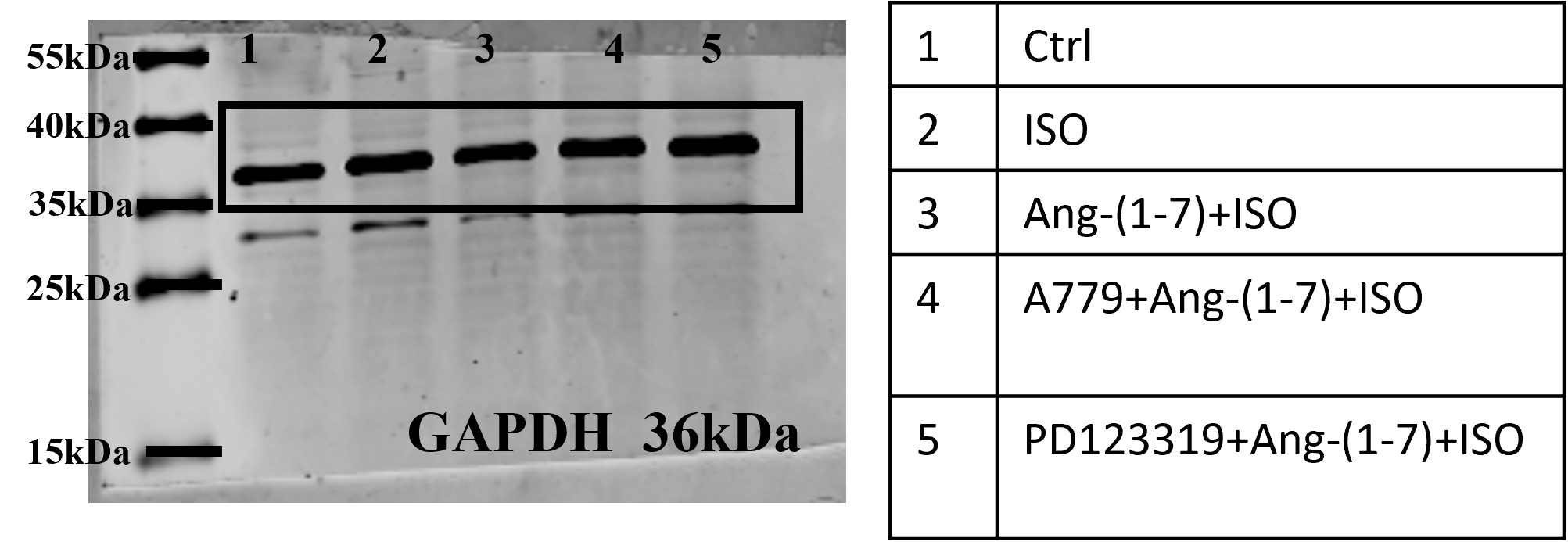


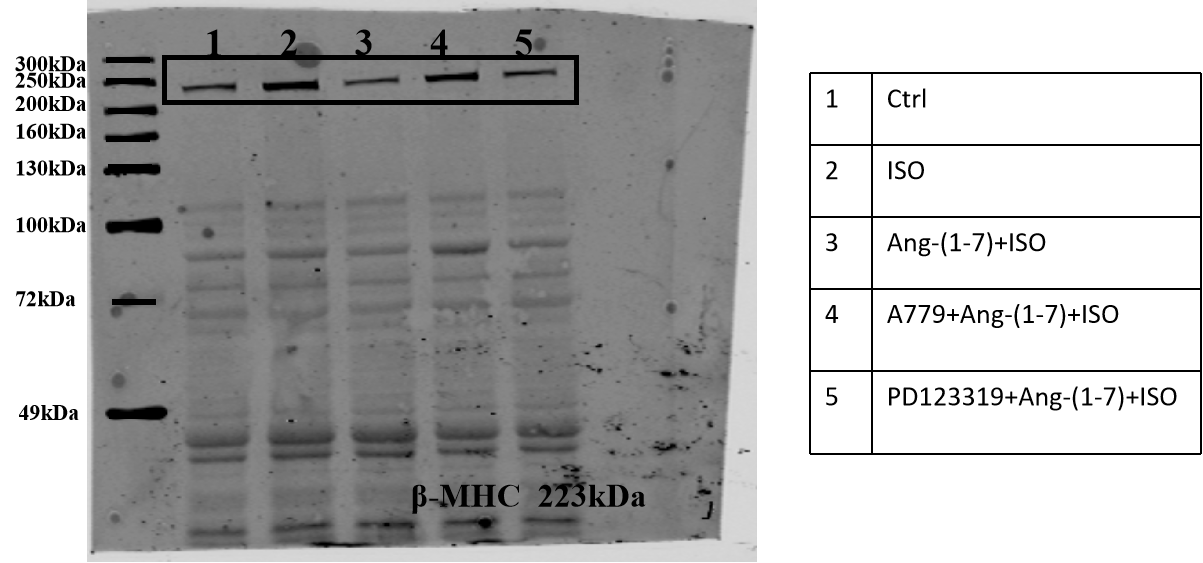

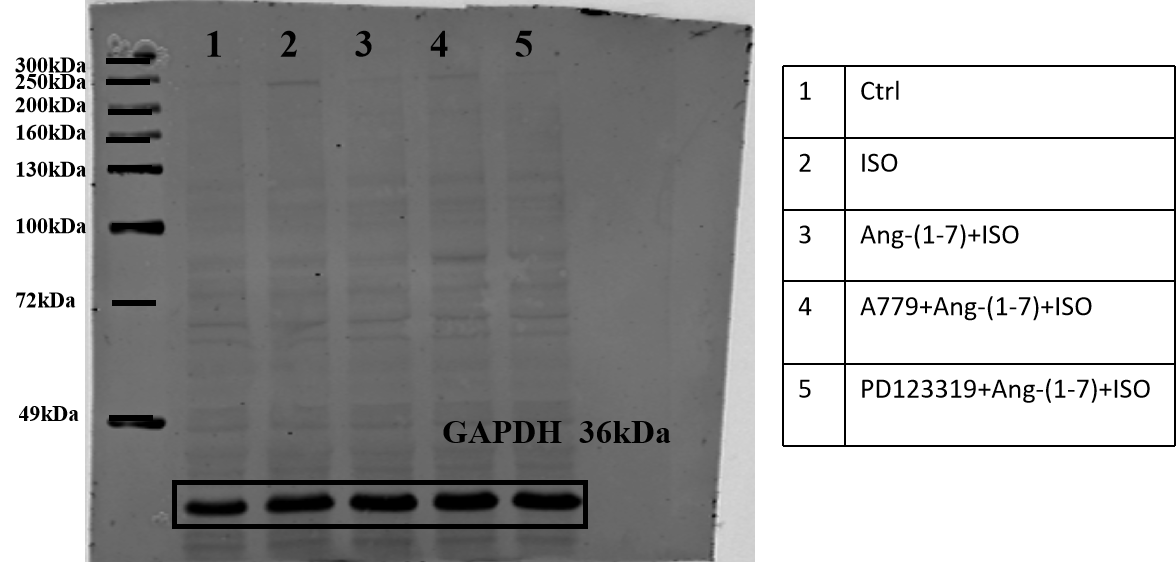


**Original image Figure-4C**

**
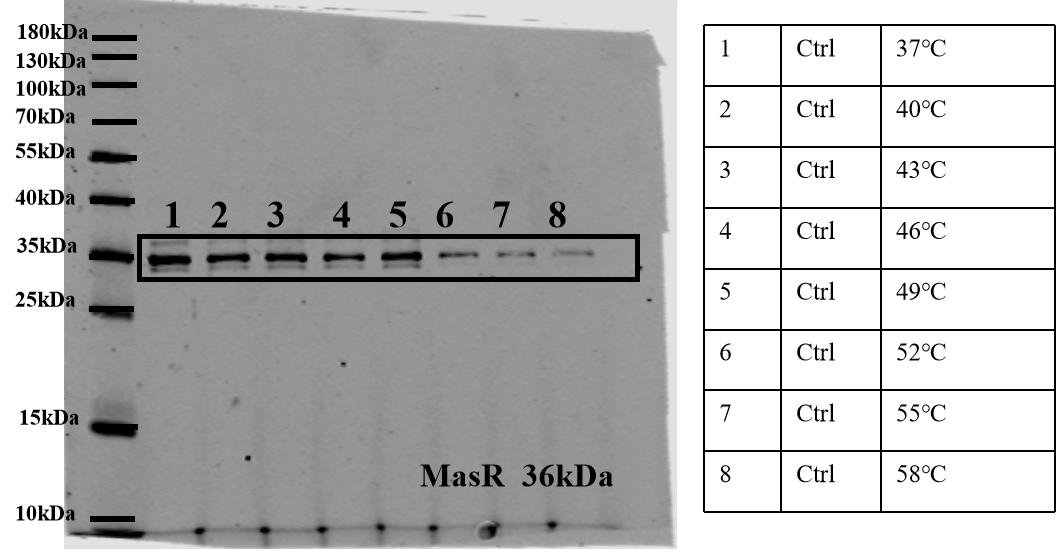

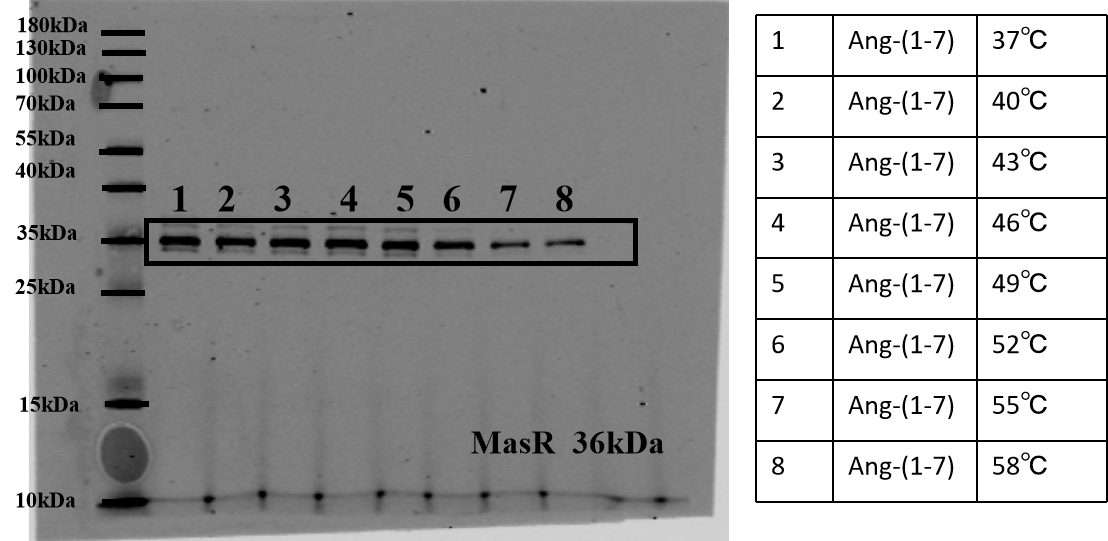
**

**Original image Figure-5B**

**
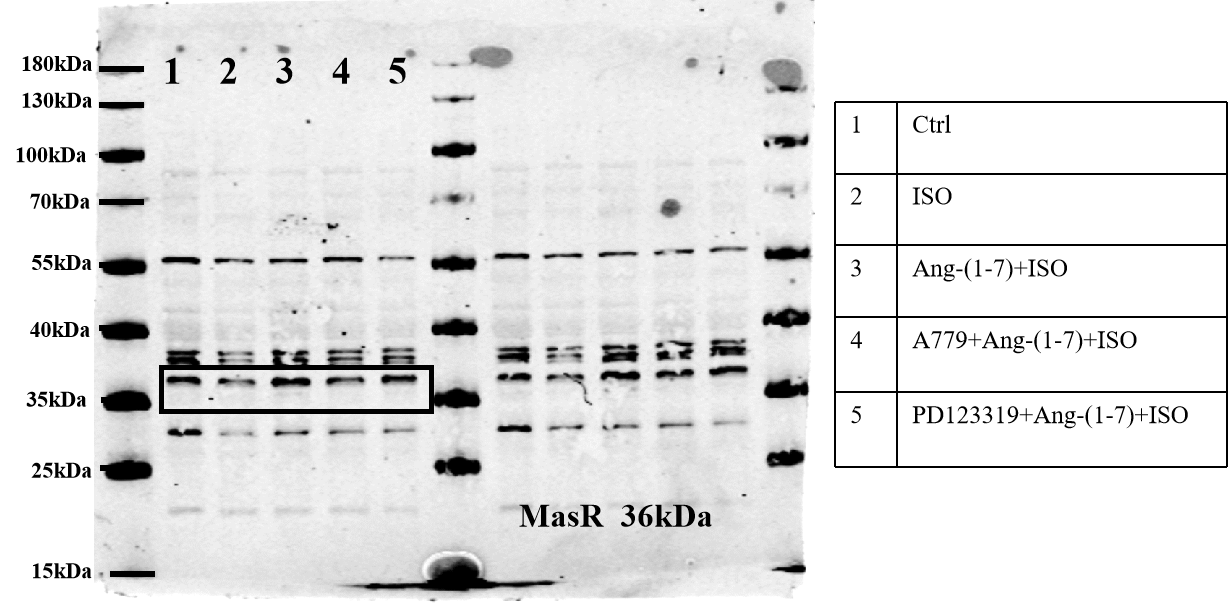

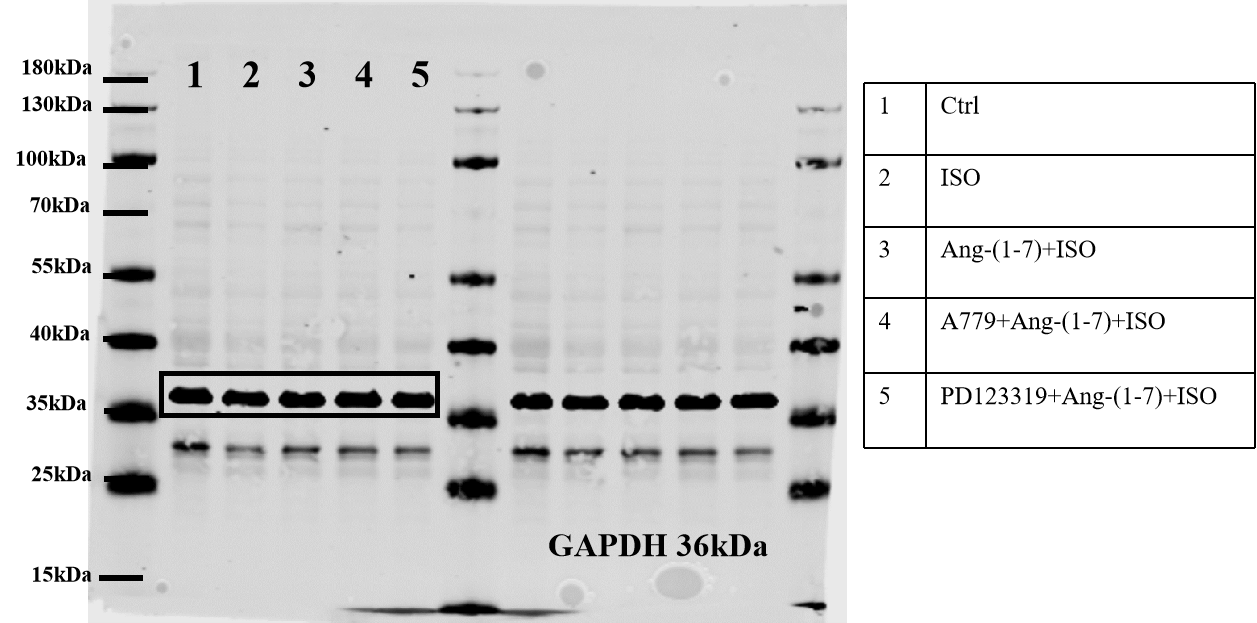
**

**
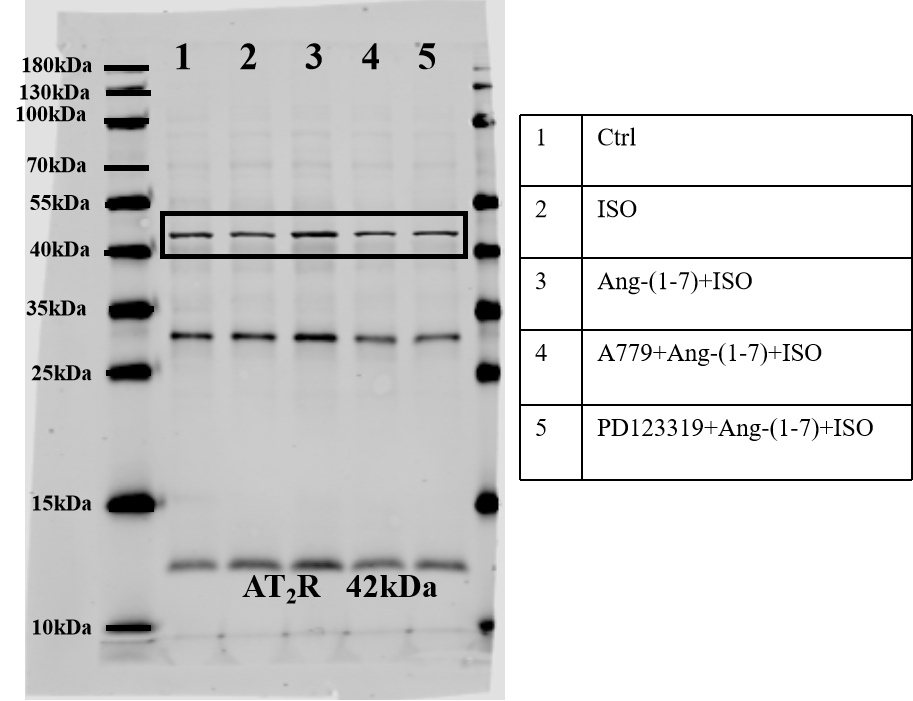
**

**
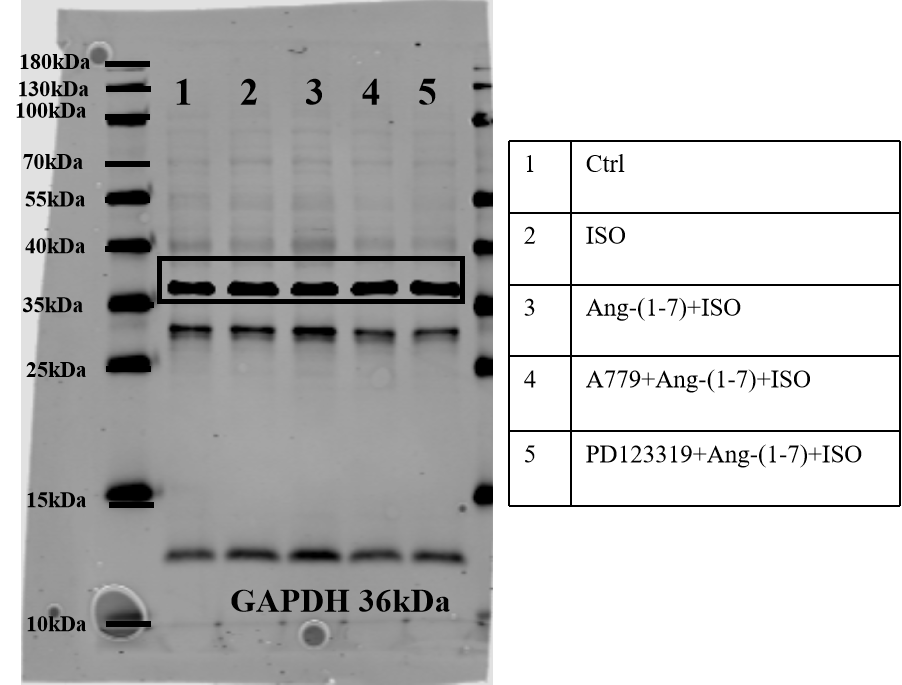
**

**Original image Figure-5D**

**
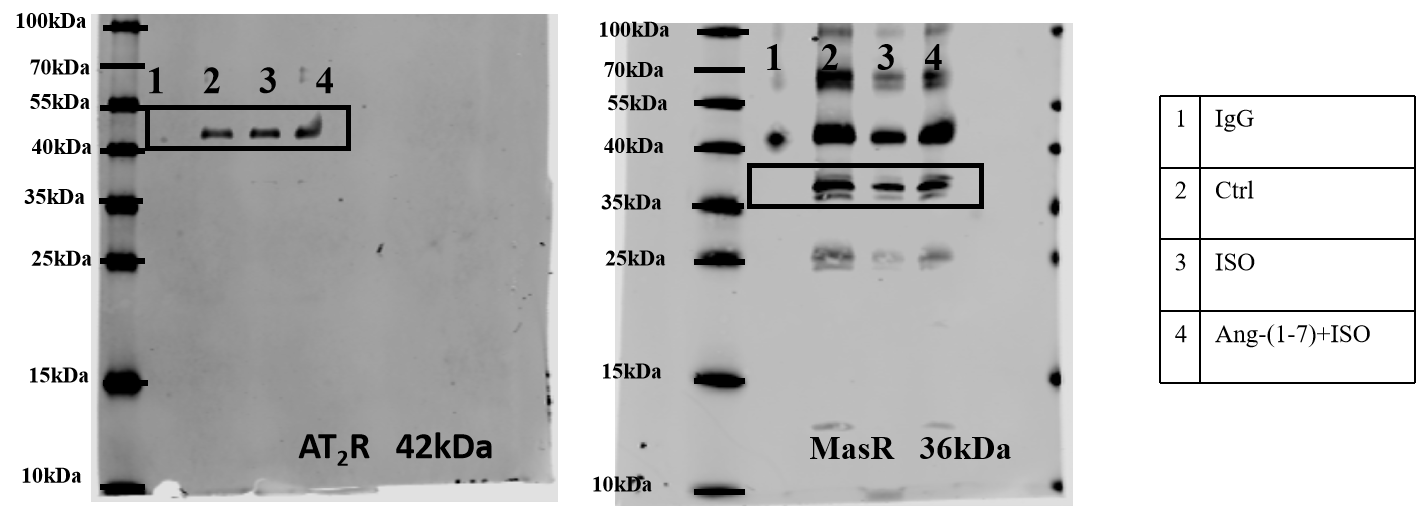
**

**
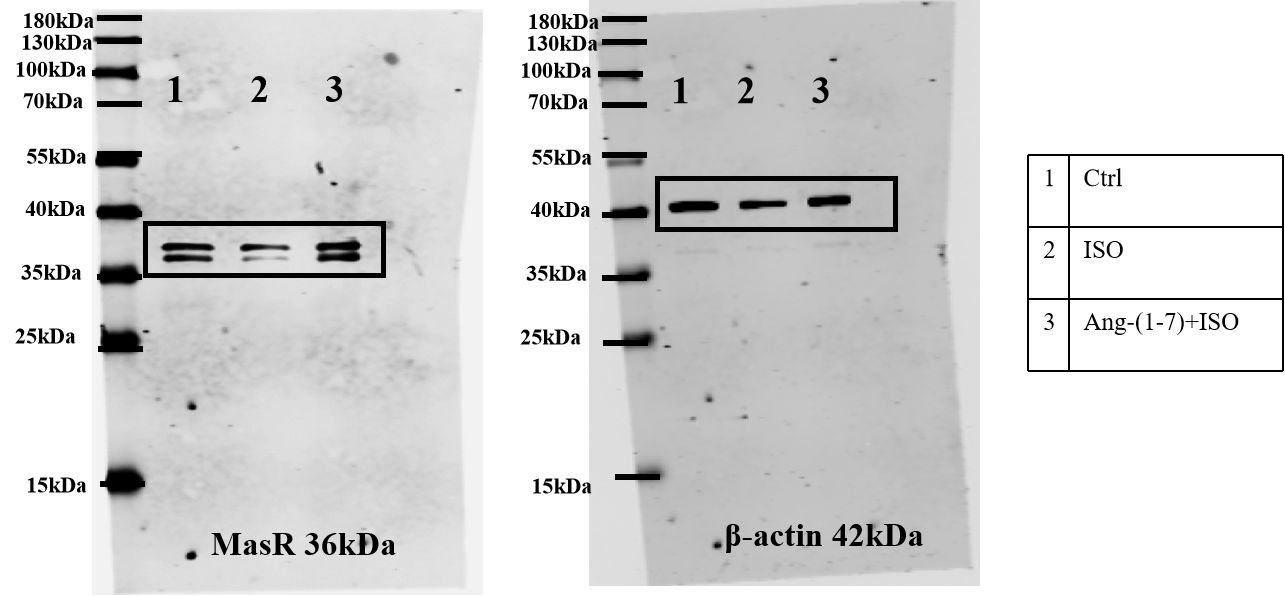
**

**
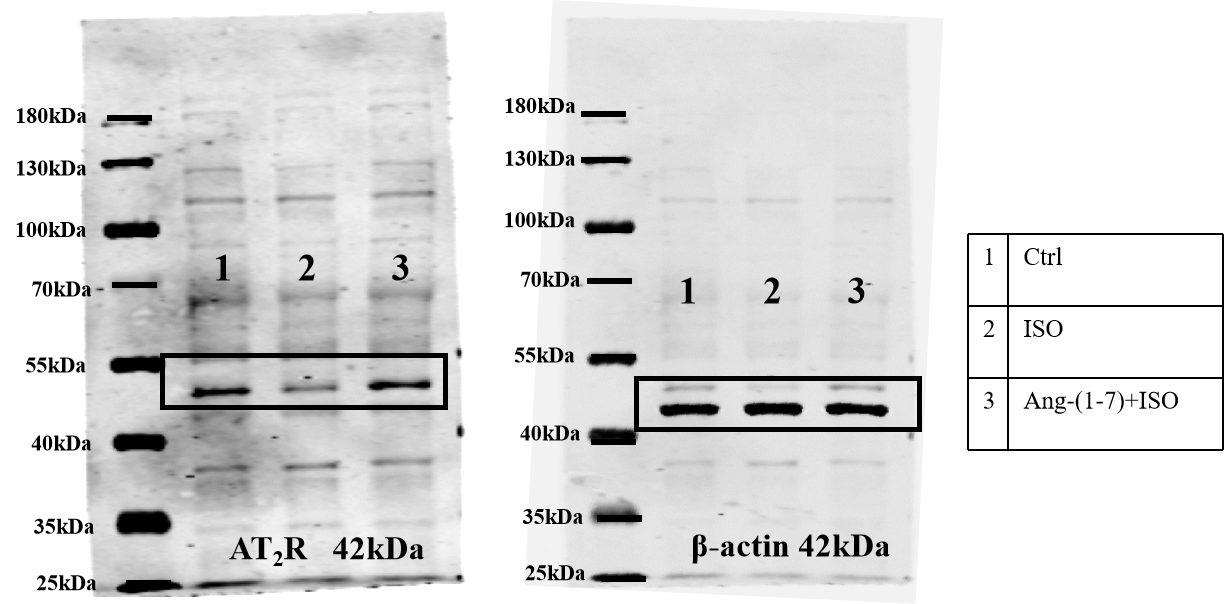
**

**Original image Figure-6B**

**
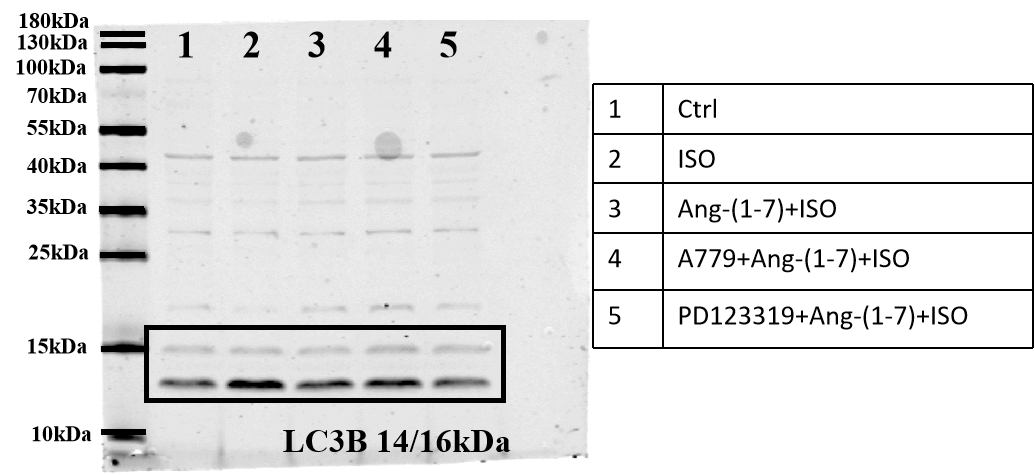

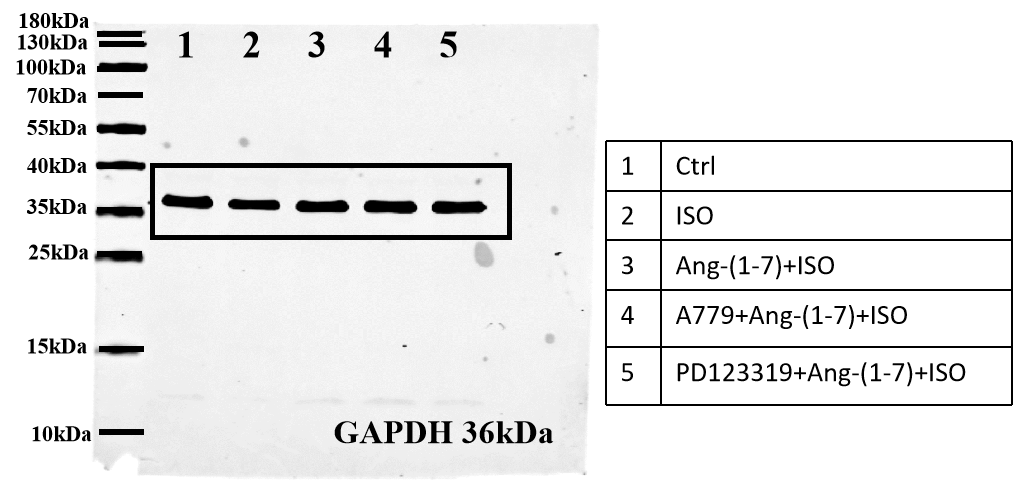
**

**
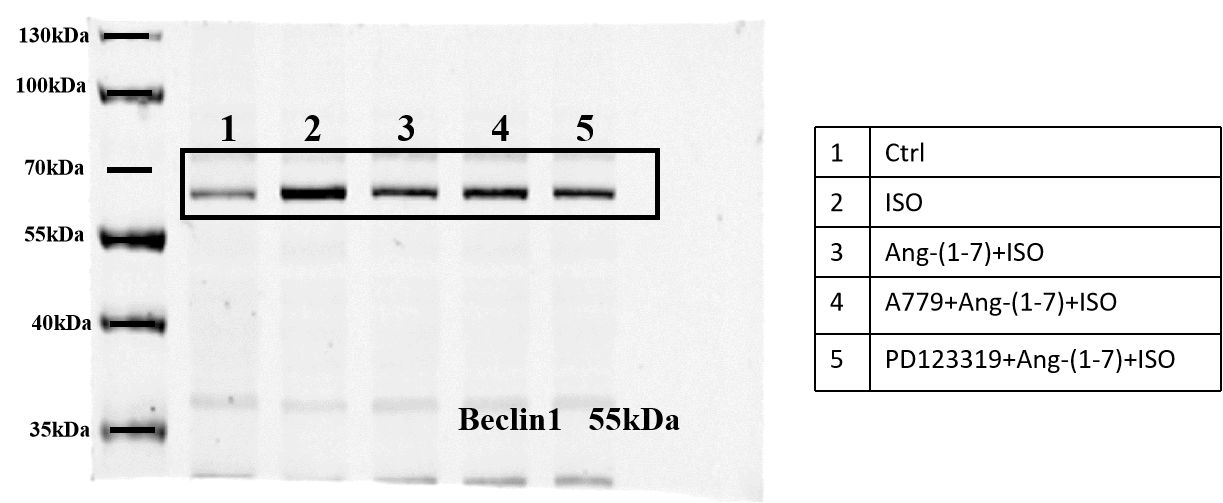

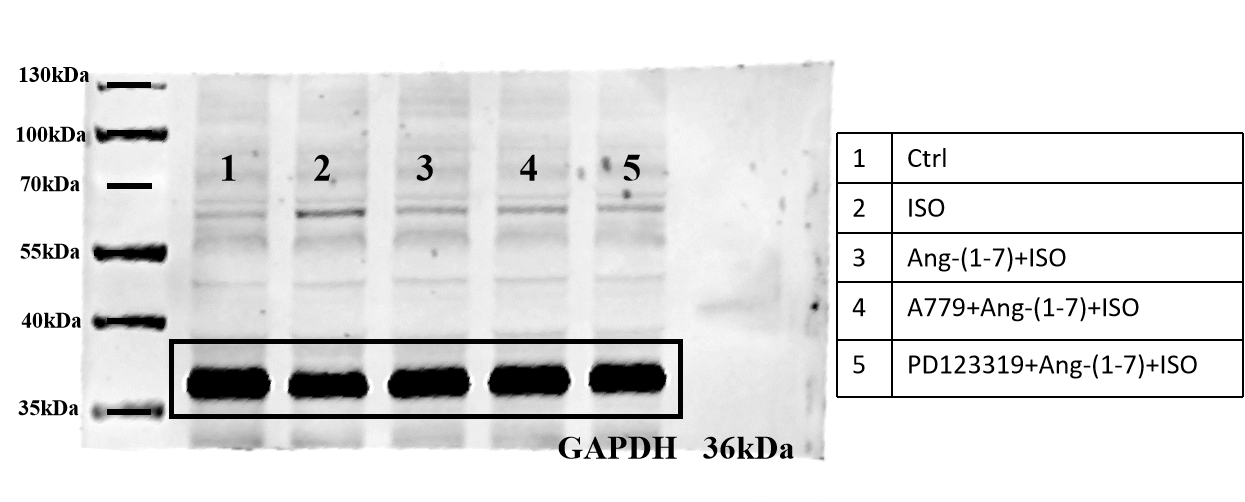
**

**
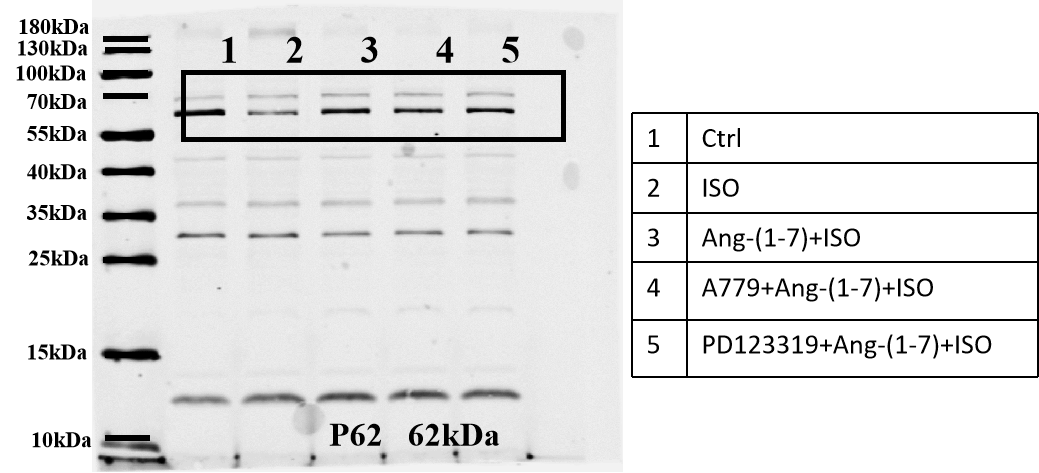
**

**
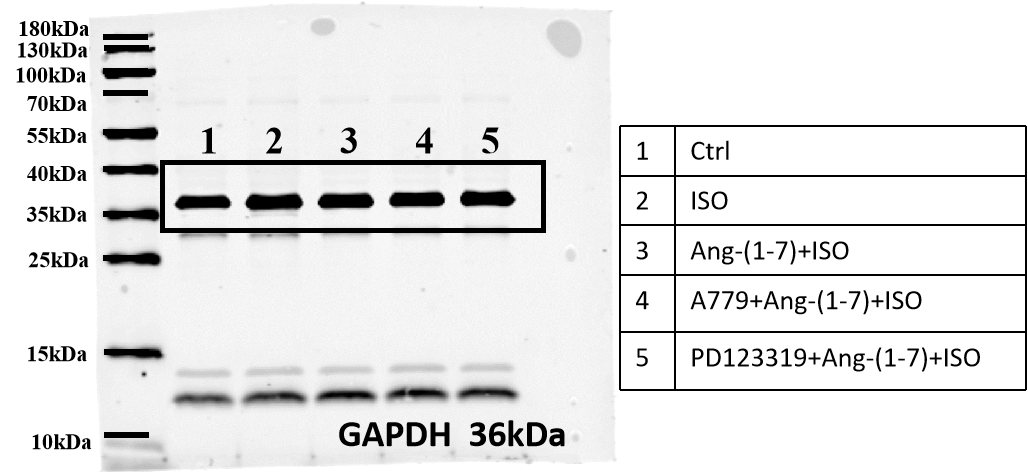
**

**Original image Figure-6C**

**
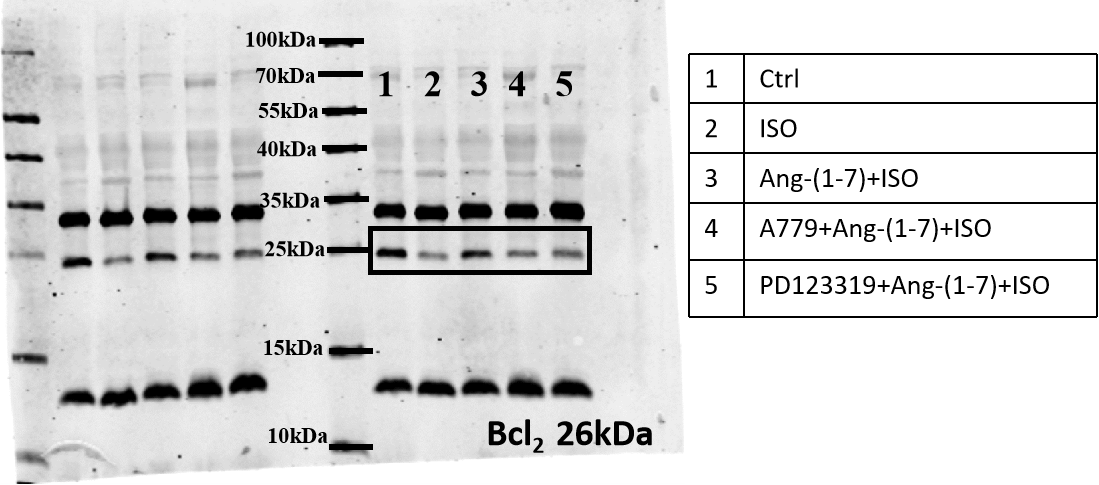

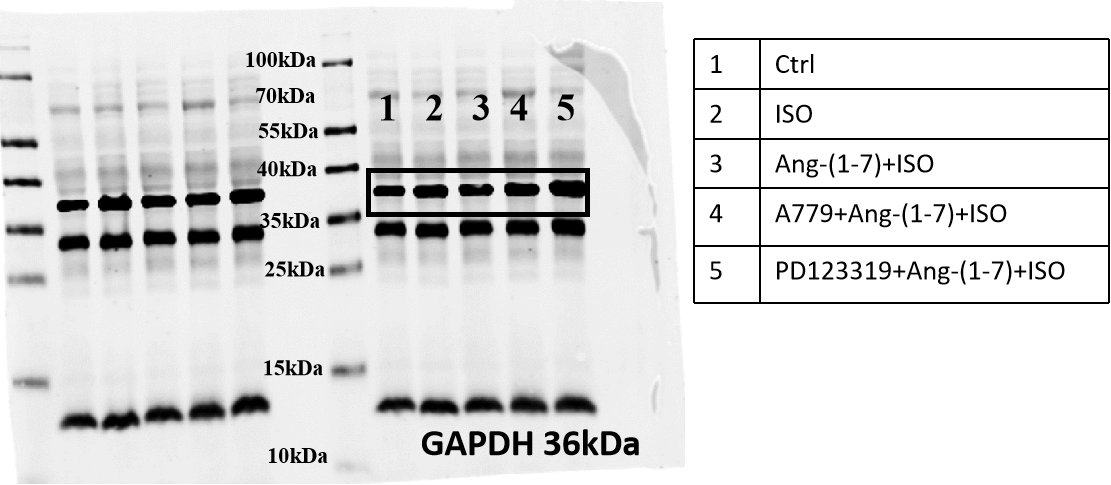
**

**
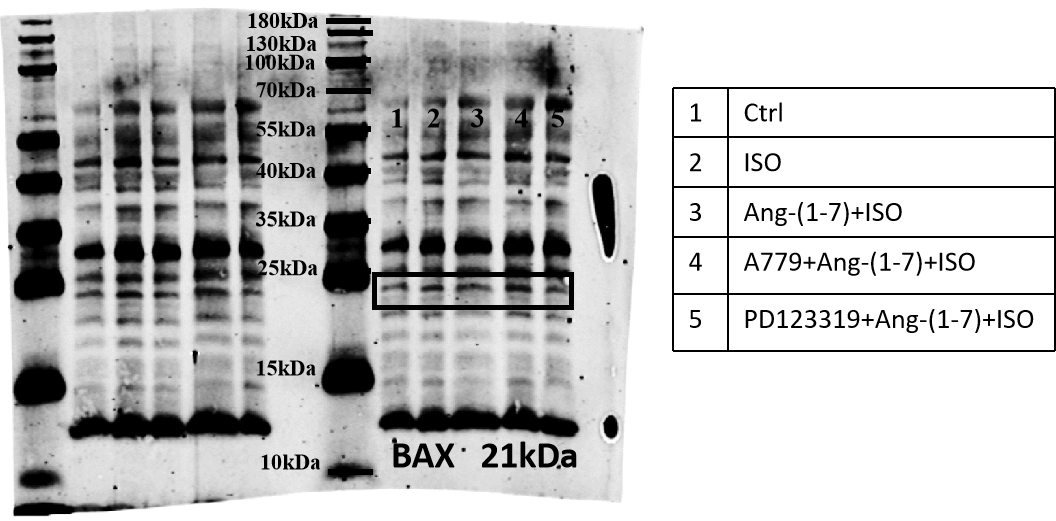

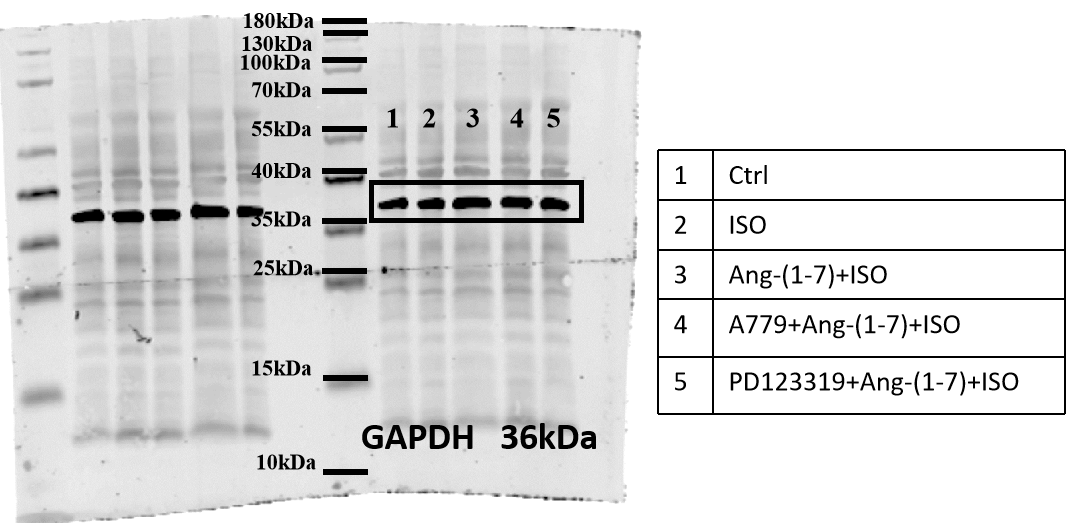
**

**
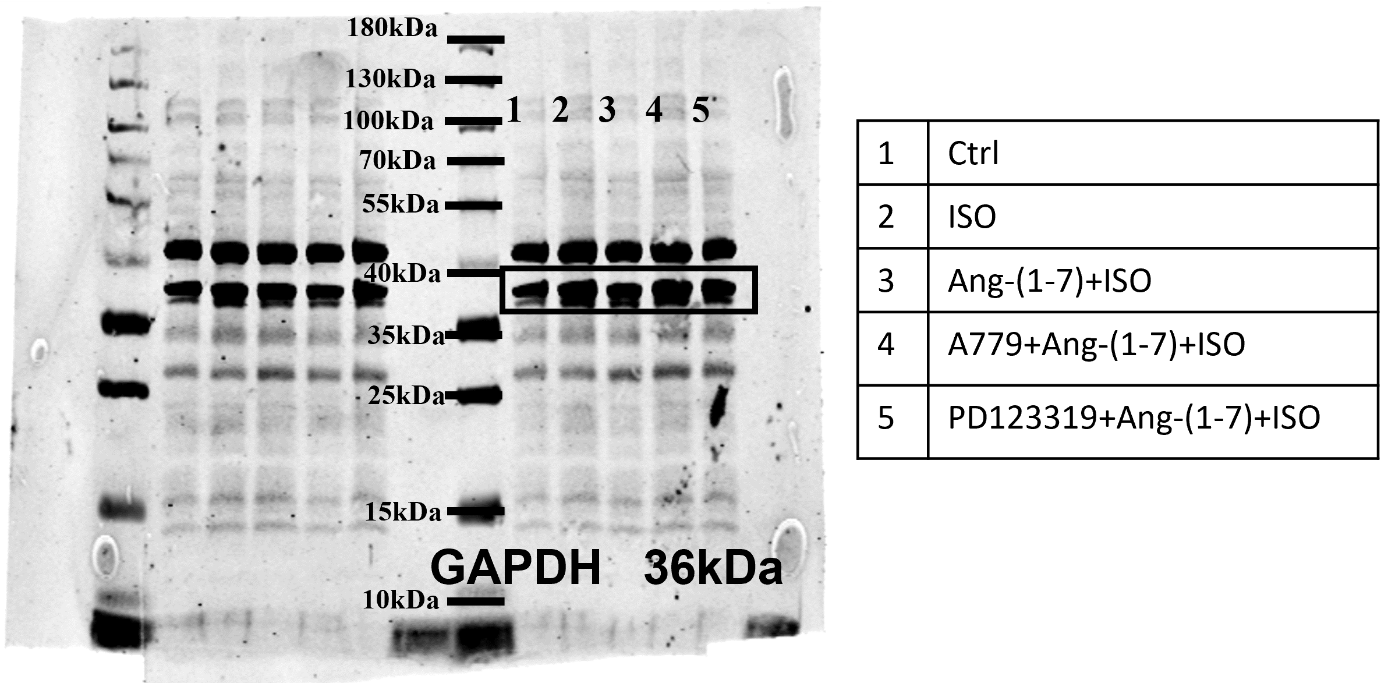
**


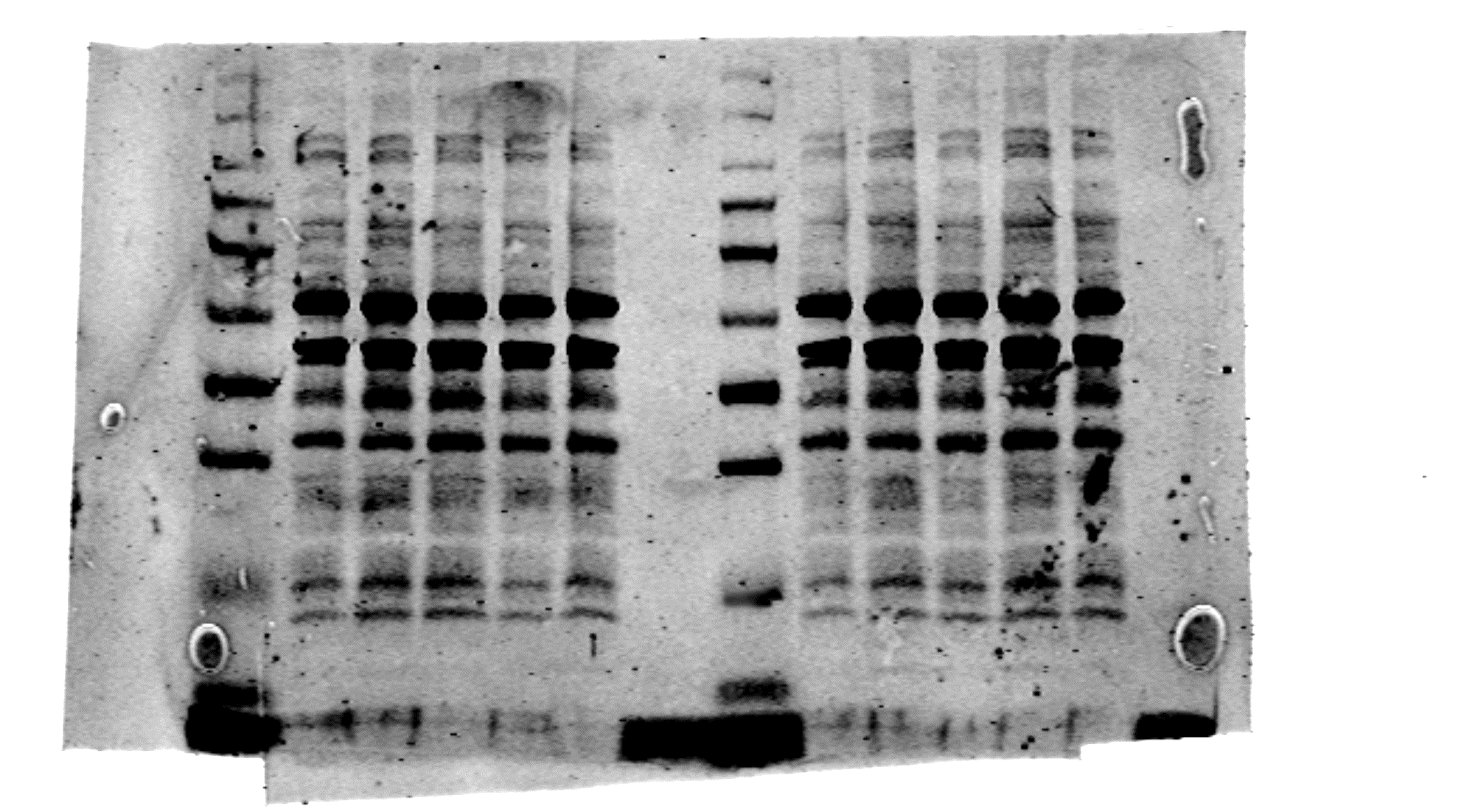


**180kDa**

**130kDa**

**100kDa**

**70kDa**

**55kDa**

**40kDa**

**35kDa**

**25kDa**

**10kDa**

**15kDa**

**1 2 3 4 5**

**Cleaved-caspase3 17kDa**


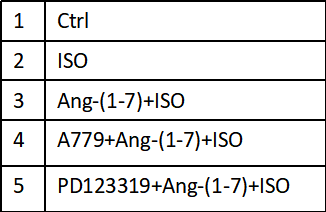


**Original image Figure-7C**


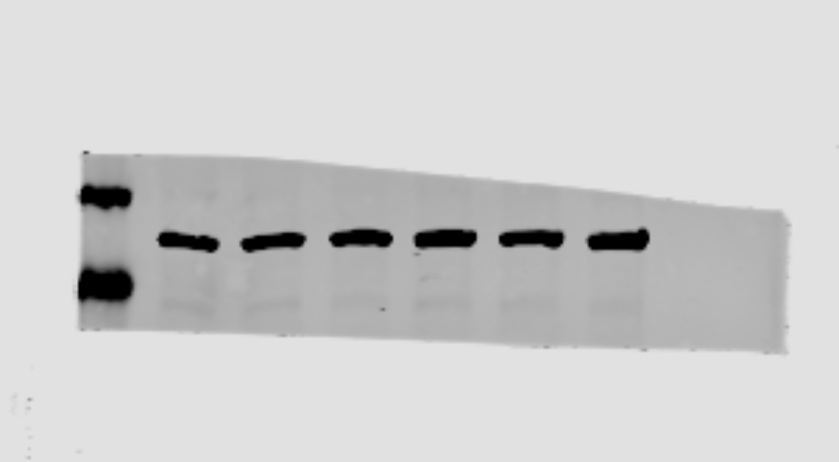

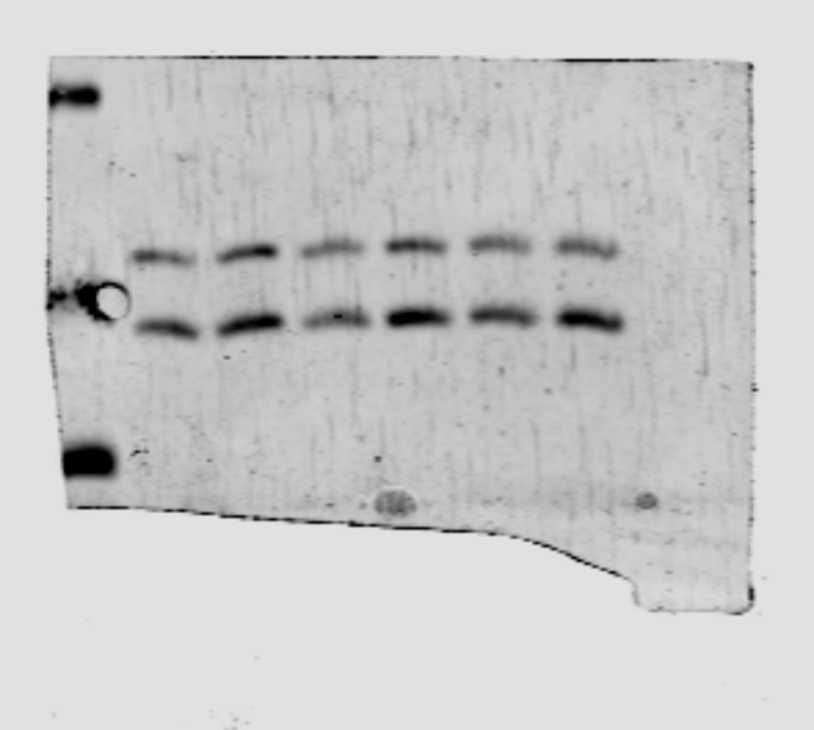


**1 2 3 4 5 6**

**40kDa**

**35kDa**

**25kDa**

**10kDa**

**15kDa**

**1 2 3 4 5 6**

LC3Ⅱ/Ⅰ 14/16kDa

GAPDH 36kDa


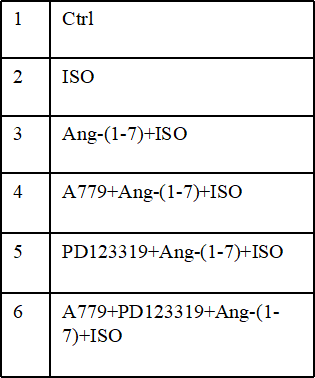


| 1 | Ctrl |
| --- | --- |
| 2 | ISO |
| 3 | Ang-(1-7)+ISO |
| 4 | A779+Ang-(1-7)+ISO |
| 5 | PD123319+Ang-(1-7)+ISO |
| 6 | A779+PD123319+Ang-(1-7)+ISO |


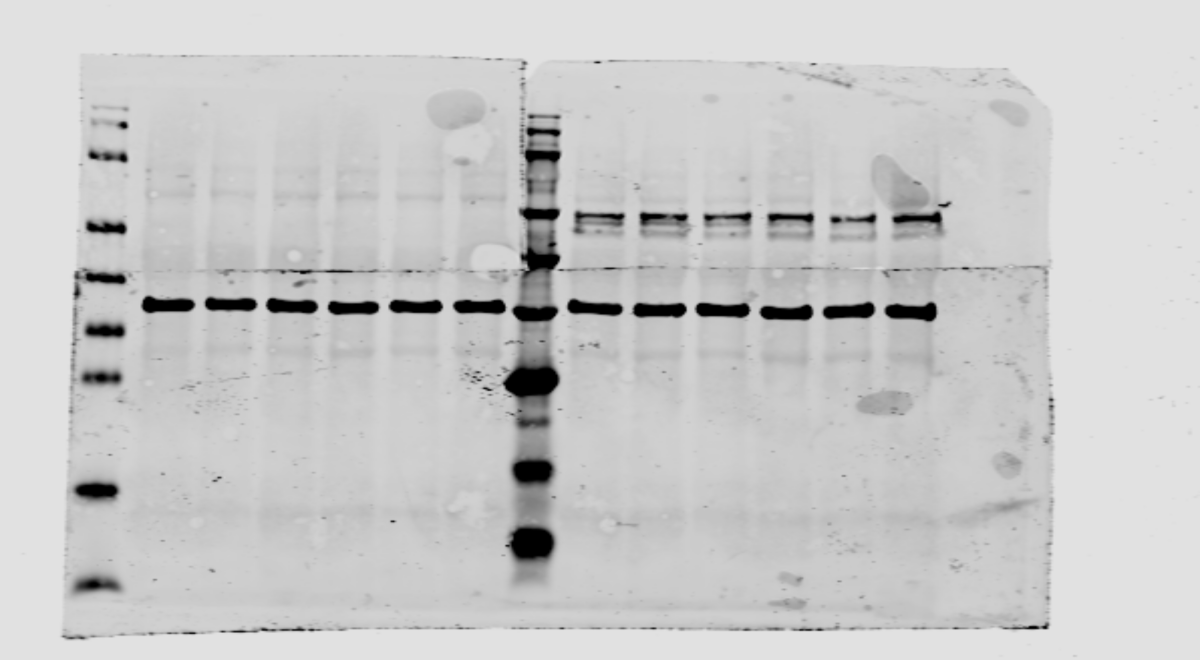


**1 2 3 4 5 6**

**130kDa**

**100kDa**

**70kDa**

**55kDa**

**40kDa**

**35kDa**

**25kDa**

**10kDa**

**15kDa**

Beclin1 55kDa

GAPDH 36kDa


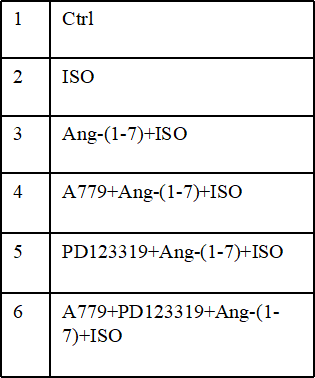


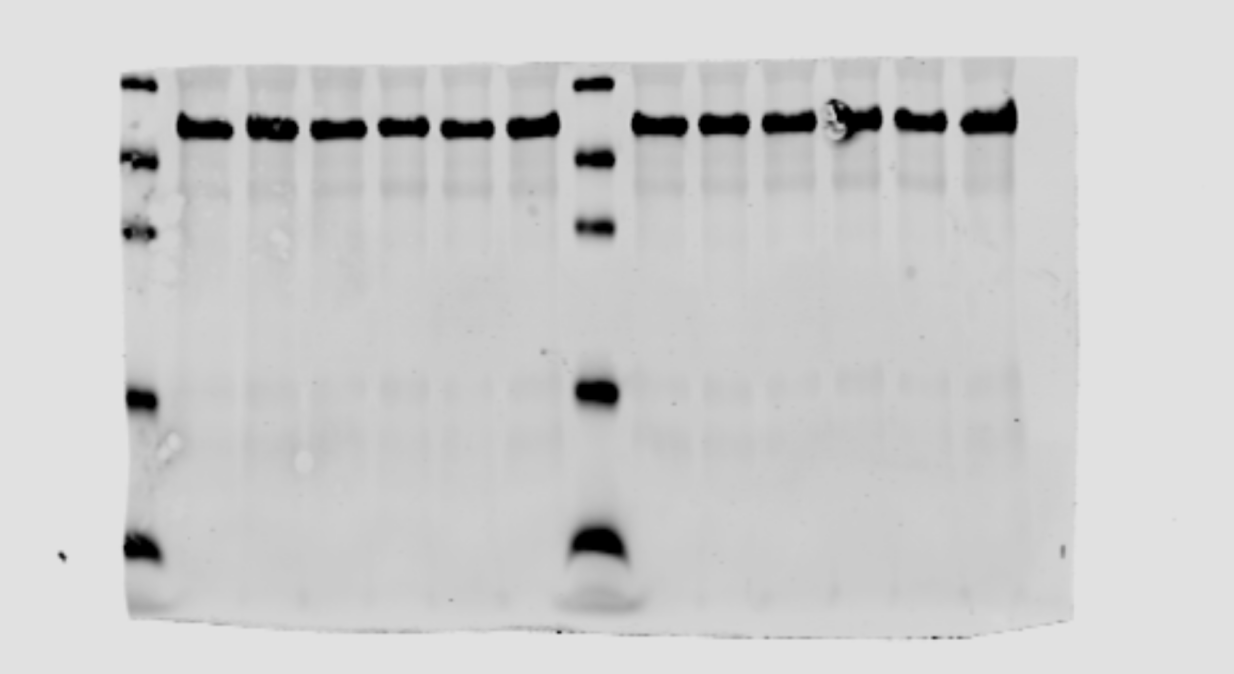

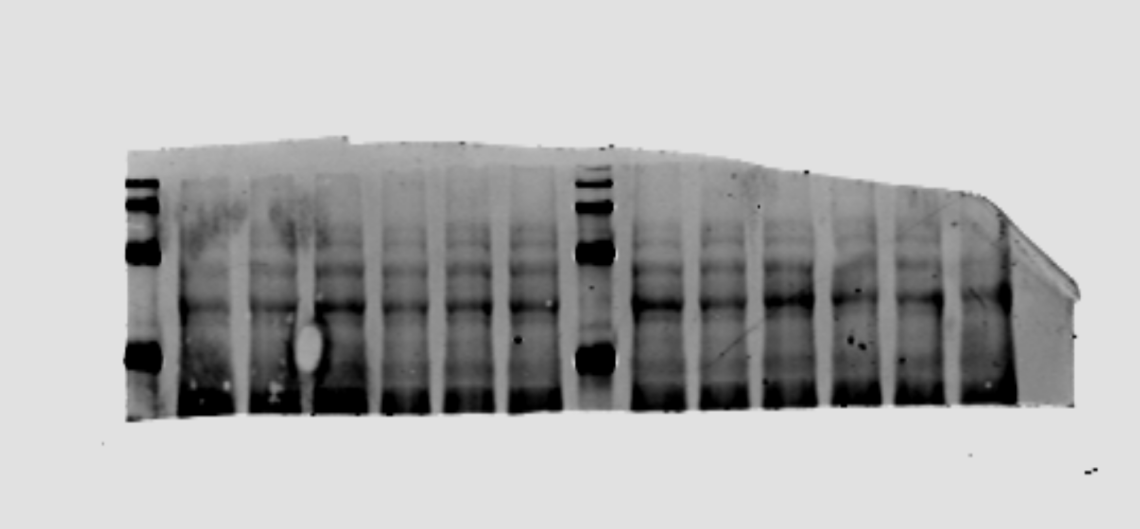


**1 2 3 4 5 6**

**180kDa**

**130kDa**

**70kDa**

**55kDa**

**35kDa**

**25kDa**

**10kDa**

**15kDa**

**100kDa**

**40kDa**

P62 62kDa

GAPDH 36kDa

**Original image Figure-7B**


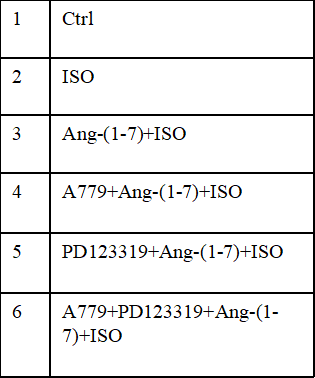


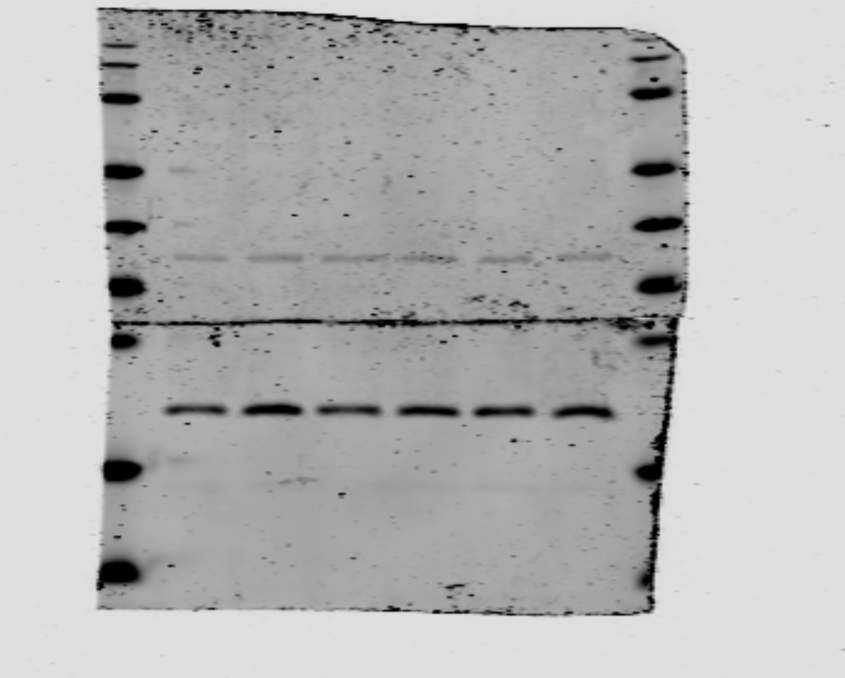


**1 2 3 4 5 6**

**180kDa**

**130kDa**

**100kDa**

**55kDa**

**40kDa**

**35kDa**

**25kDa**

**10kDa**

**15kDa**

**70kDa**

cleaved-caspase3 17kDa


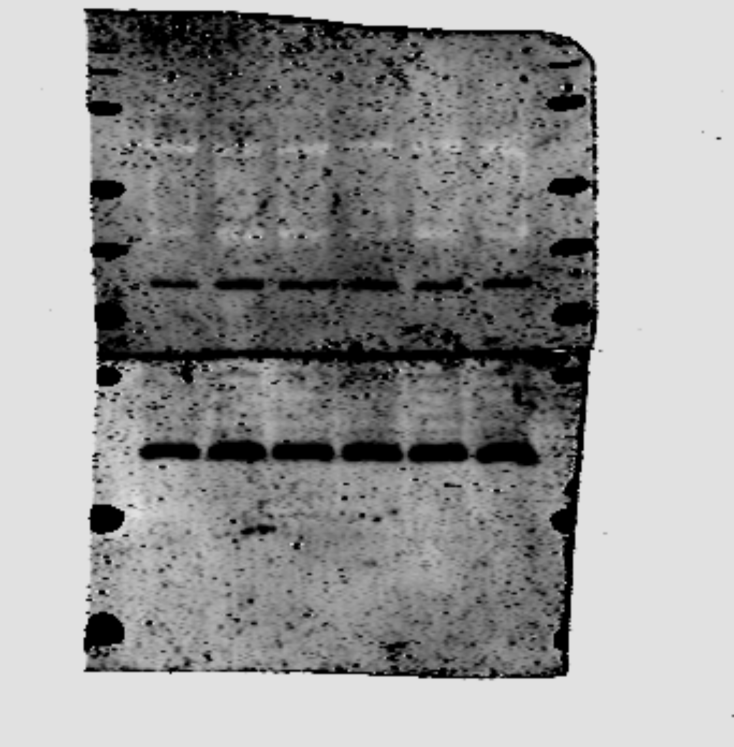


**1 2 3 4 5 6**

**180kDa**

**130kDa**

**100kDa**

**55kDa**

**40kDa**

**35kDa**

**25kDa**

**10kDa**

**15kDa**

**70kDa**

GAPDH 36kDa


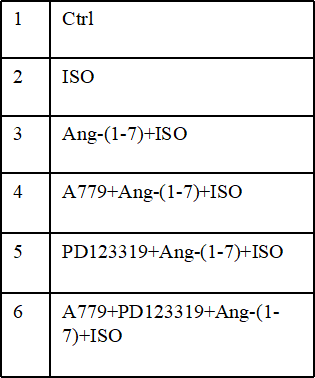


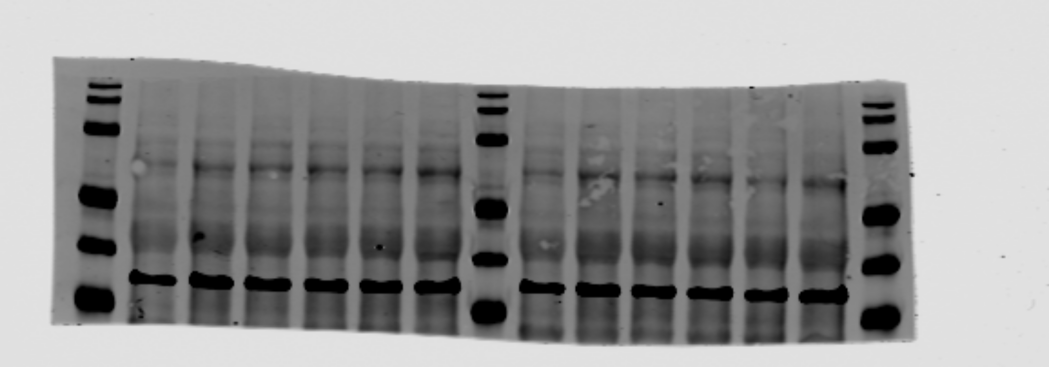

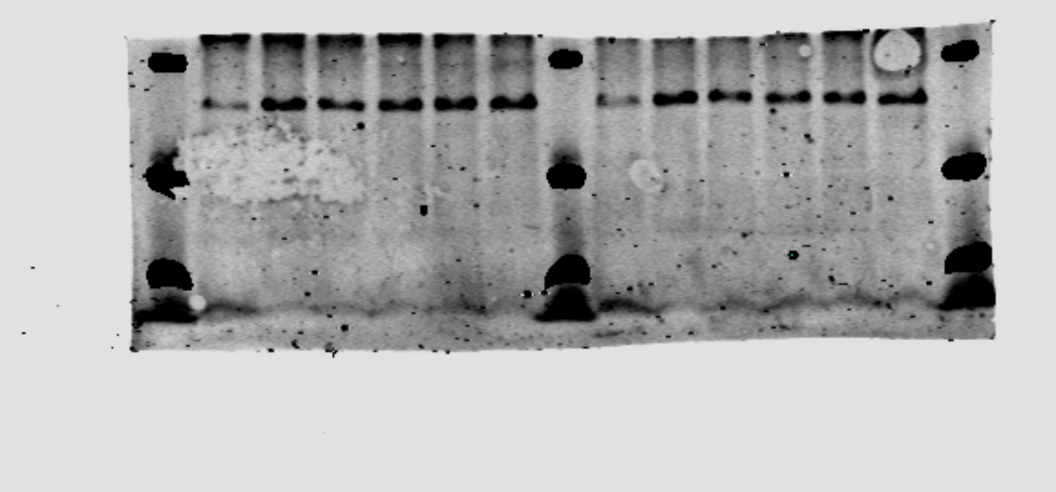


**1 2 3 4 5 6**

**130kDa**

**100kDa**

**70kDa**

**55kDa**

**40kDa**

**35kDa**

**25kDa**

**10kDa**

**15kDa**

Bax 21 kDa

GAPDH 36kDa


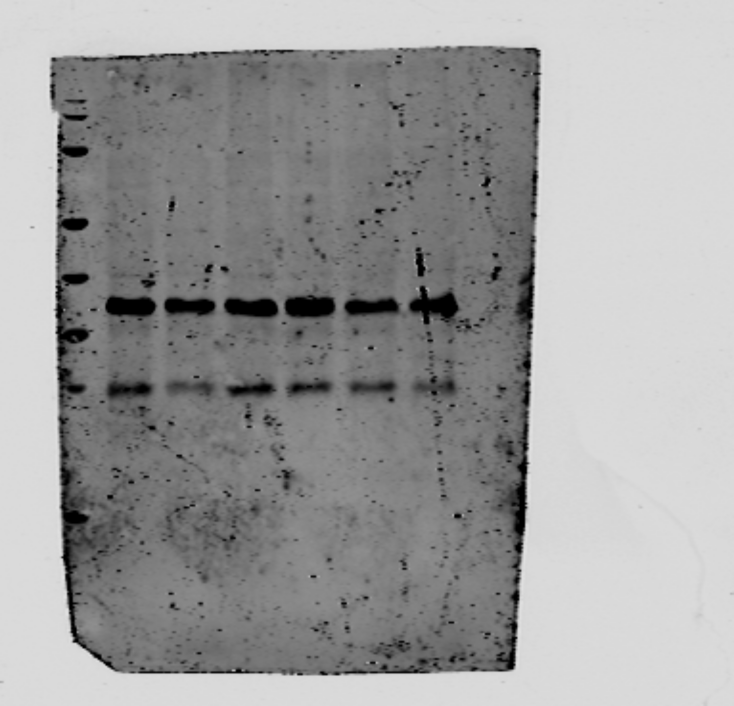


**1 2 3 4 5 6**

**130kDa**

**100kDa**

**55kDa**

**40kDa**

**35kDa**

**25kDa**

**15kDa**

**70kDa**

**Bcl_2_ 17kDa**


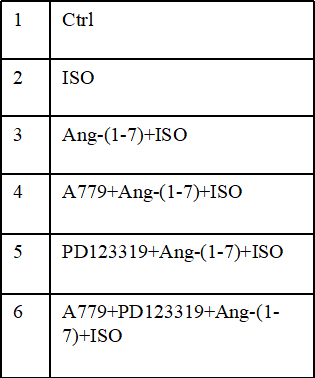


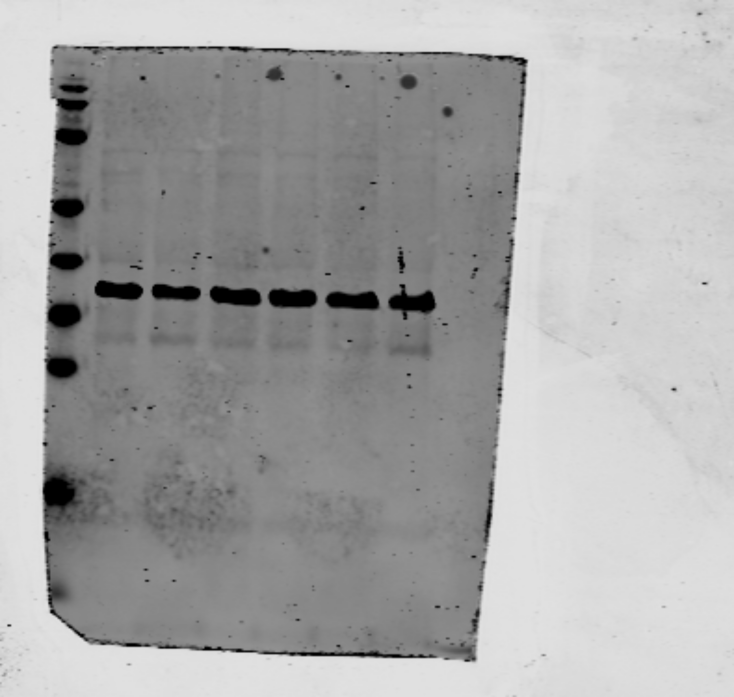


**1 2 3 4 5 6**

**130kDa**

**100kDa**

**55kDa**

**40kDa**

**35kDa**

**25kDa**

**15kDa**

**70kDa**

**GAPDH 36kDa**


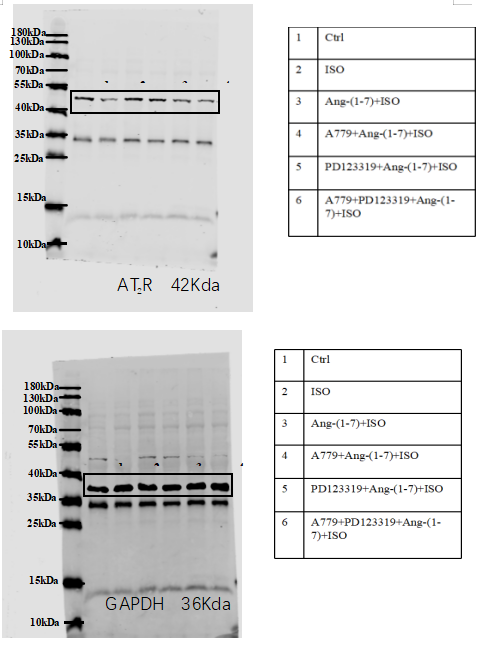


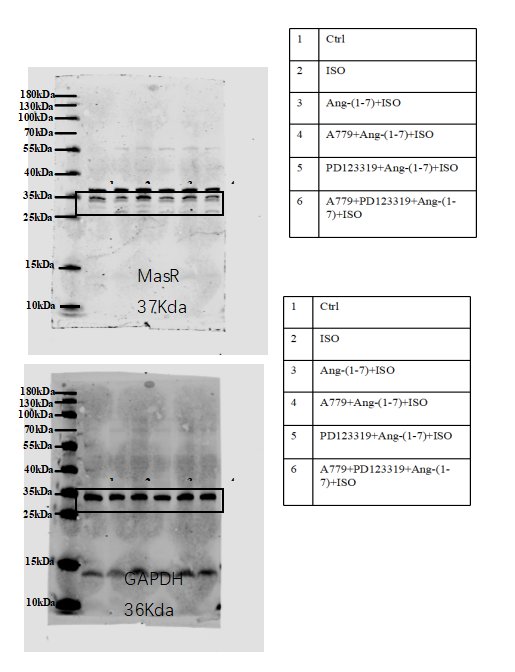


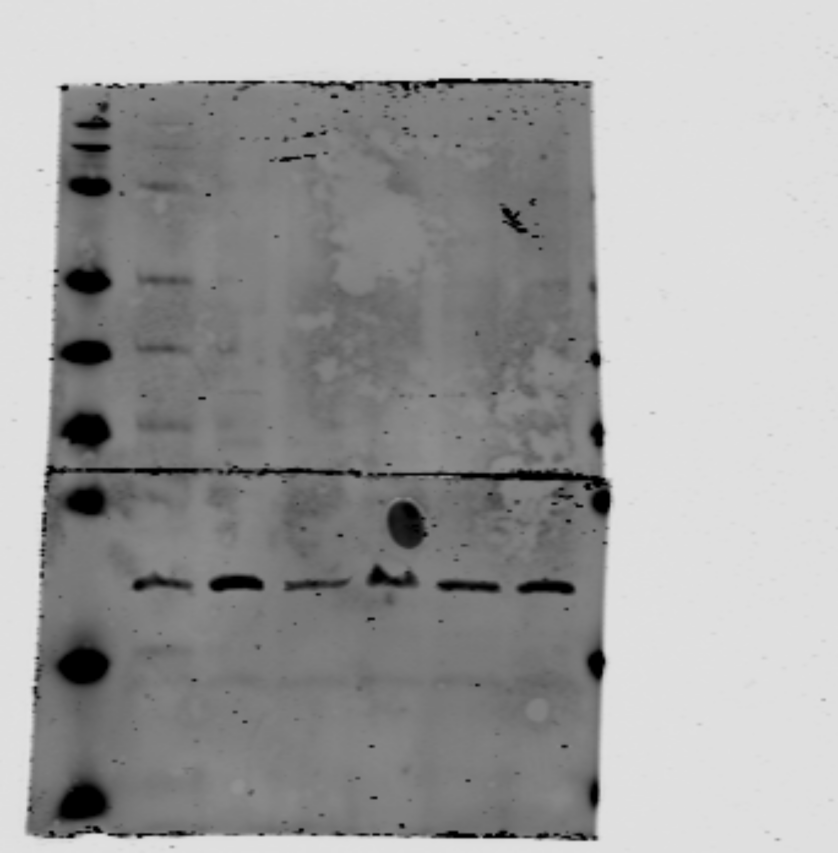


**130kDa**

**100kDa**

**55kDa**

**40kDa**

**35kDa**

**25kDa**

**10kDa**

**70kDa**

**180kDa**

**15kDa**

**1 2 3 4 5 6**


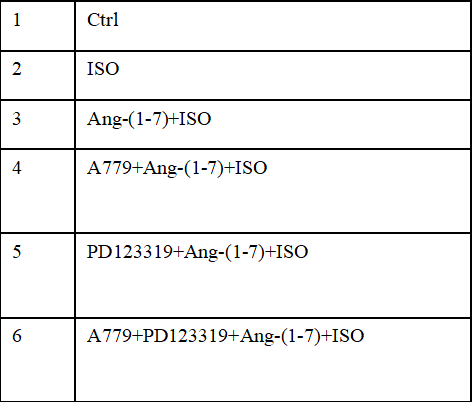


**ANP 17KDa**


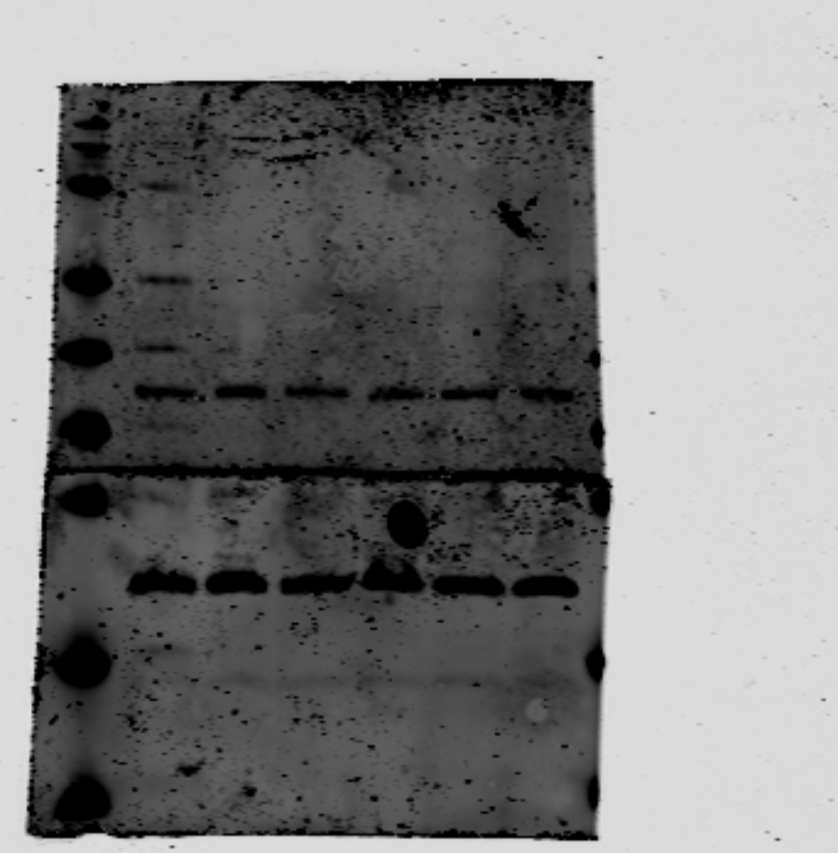


**1 2 3 4 5 6**

**130kDa**

**100kDa**

**55kDa**

**40kDa**

**35kDa**

**25kDa**

**10kDa**

**70kDa**

**180kDa**

**15kDa**

**GAPDH 36Kda**


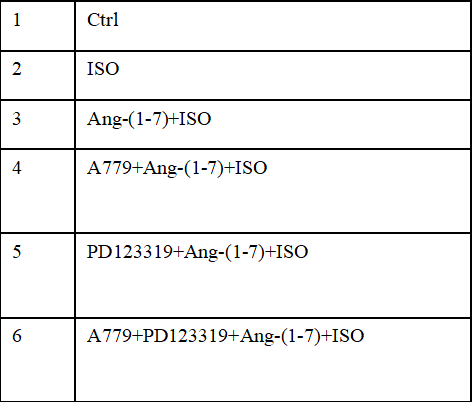


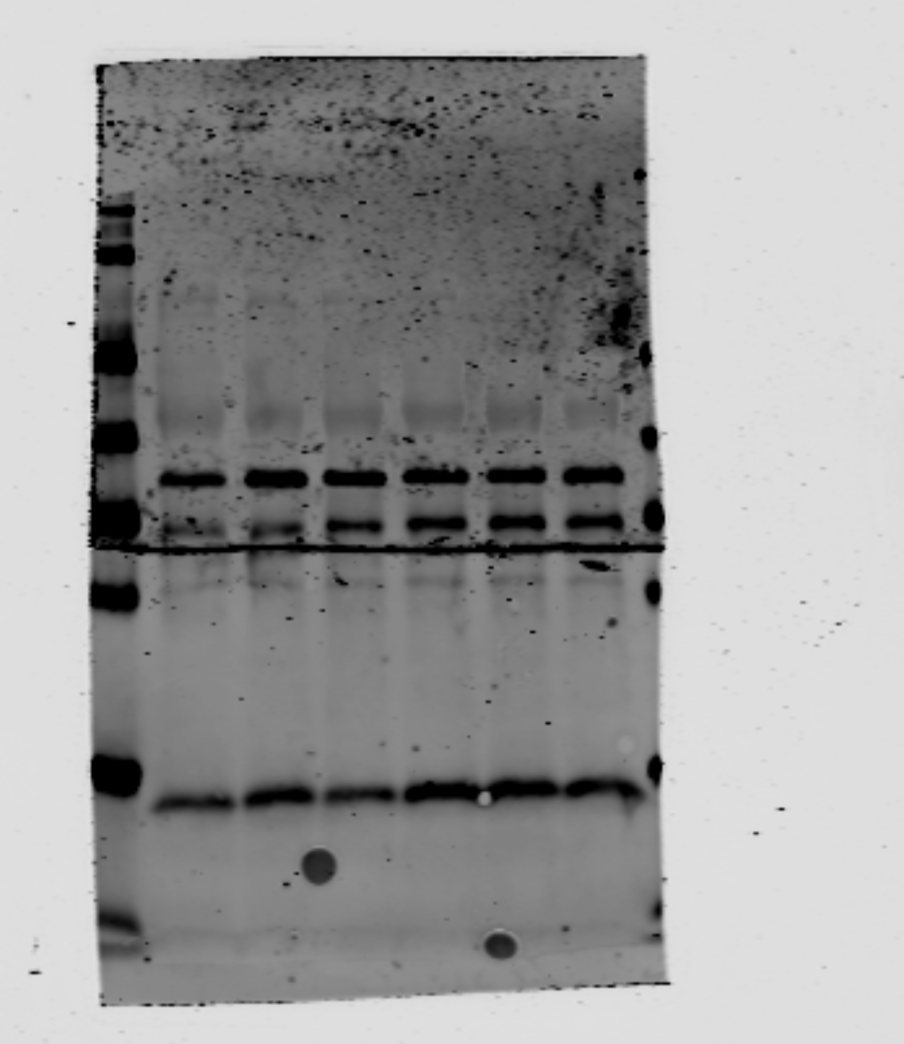


**1 2 3 4 5 6**

**100kDa**

**55kDa**

**40kDa**

**35kDa**

**25kDa**

**10kDa**

**70kDa**

**15kDa**

**BNP 14Kda**

**GAPDH 36kDa**


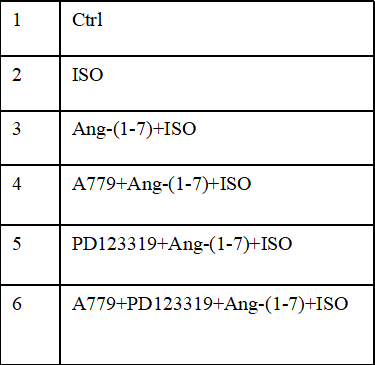


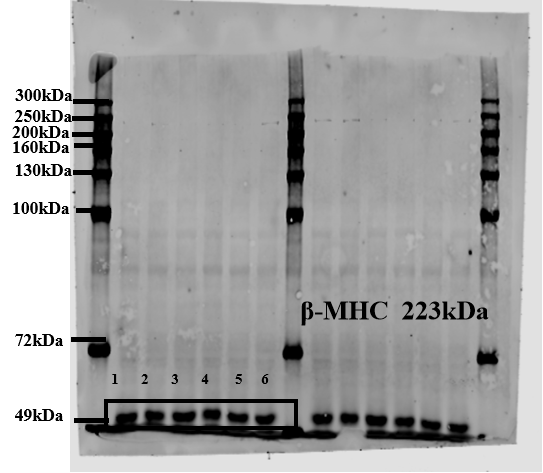

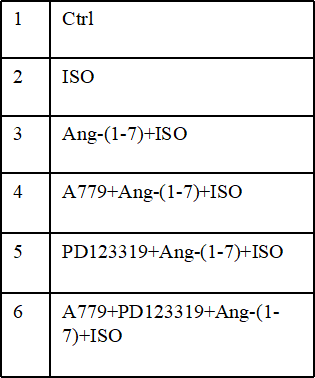


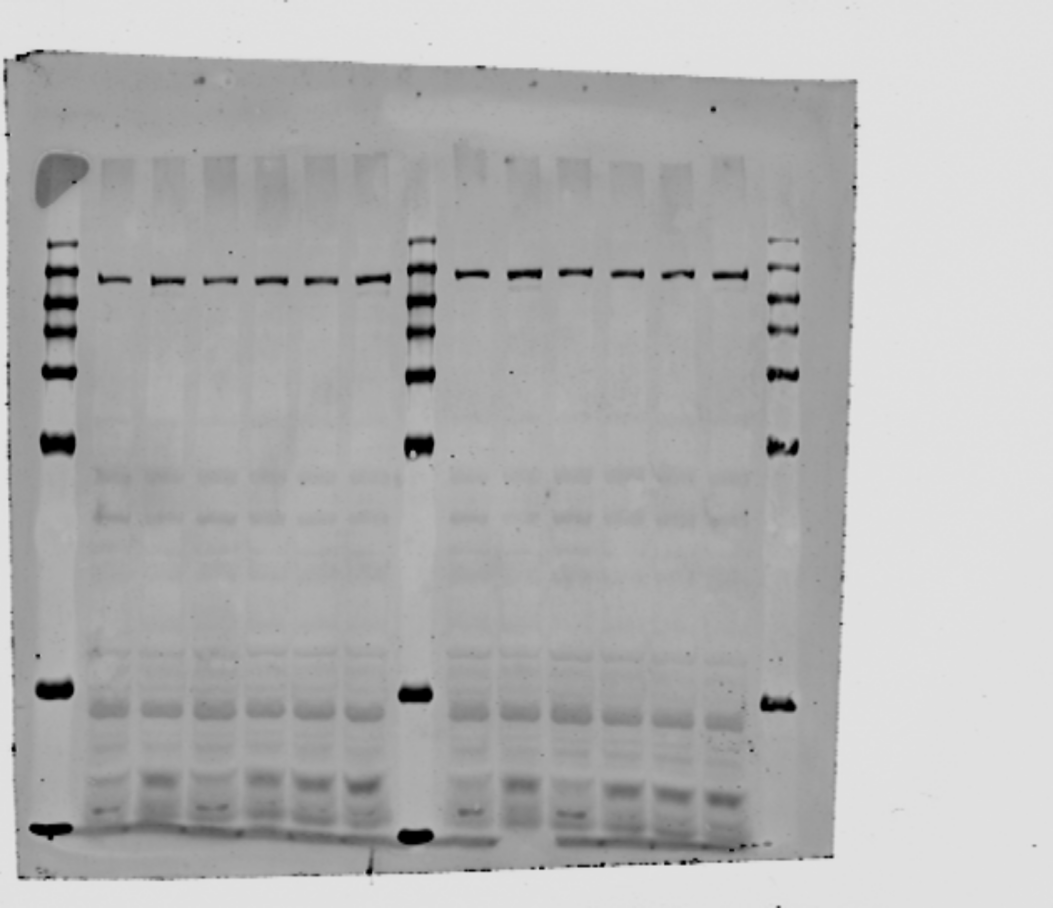


**1 2 3 4 5 6**

**β-MHC 223kDa**

**300kDa**

**250kDa**

**200kDa**

**160kDa**

**130kDa**

**100kDa**

**72kDa**

**49kDa**


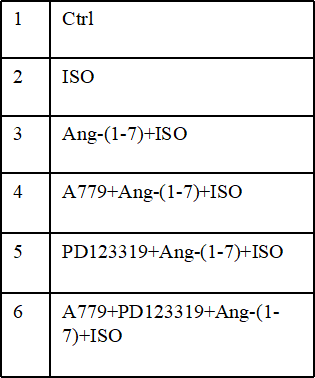


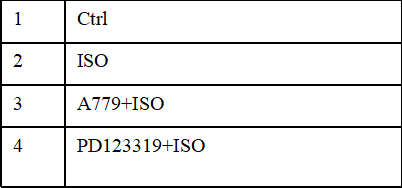


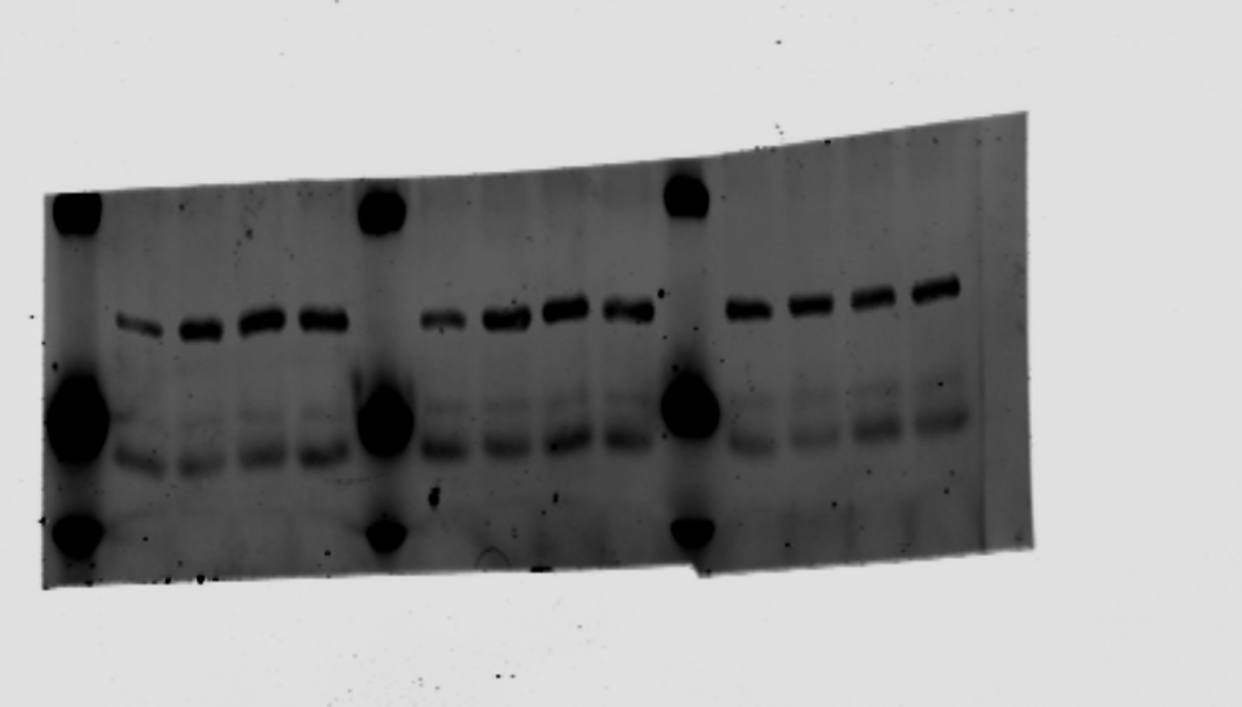

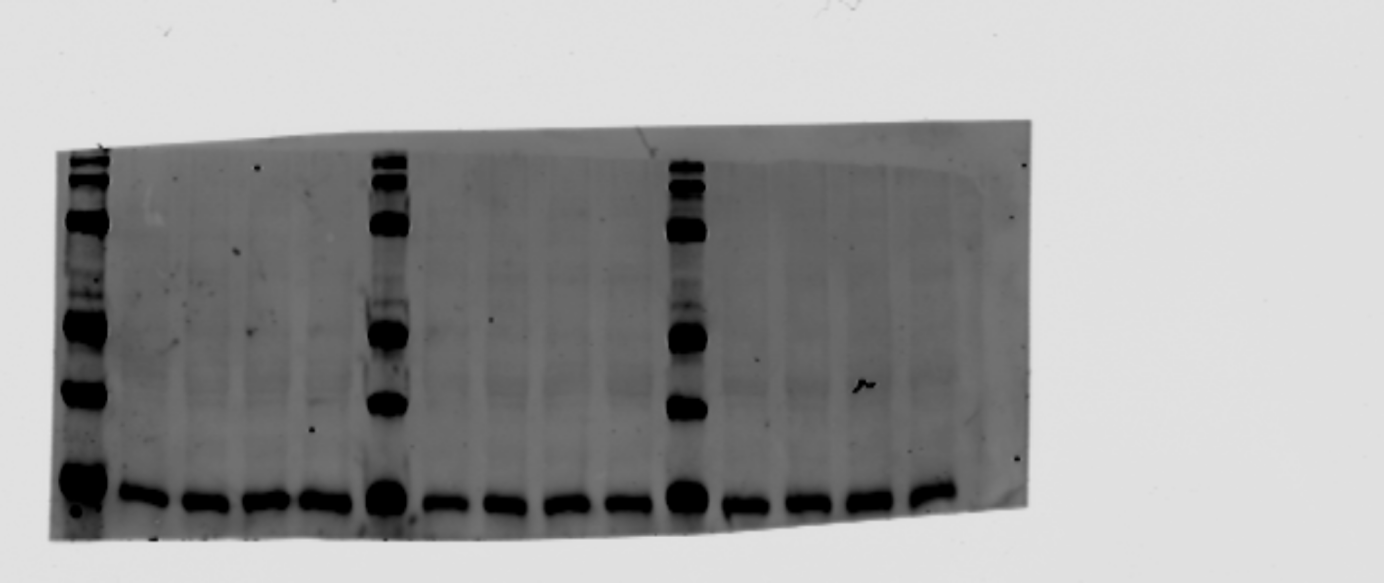


**180kDa**

**130kDa**

**70kDa**

**40kDa**

**35kDa**

**25kDa**

**15kDa**

**10kDa**

**55kDa**

**100kDa**

**1 2 3 4**

**ANP 17 kDa**

**GAPDH 36kDa**

**1 2 3 4**


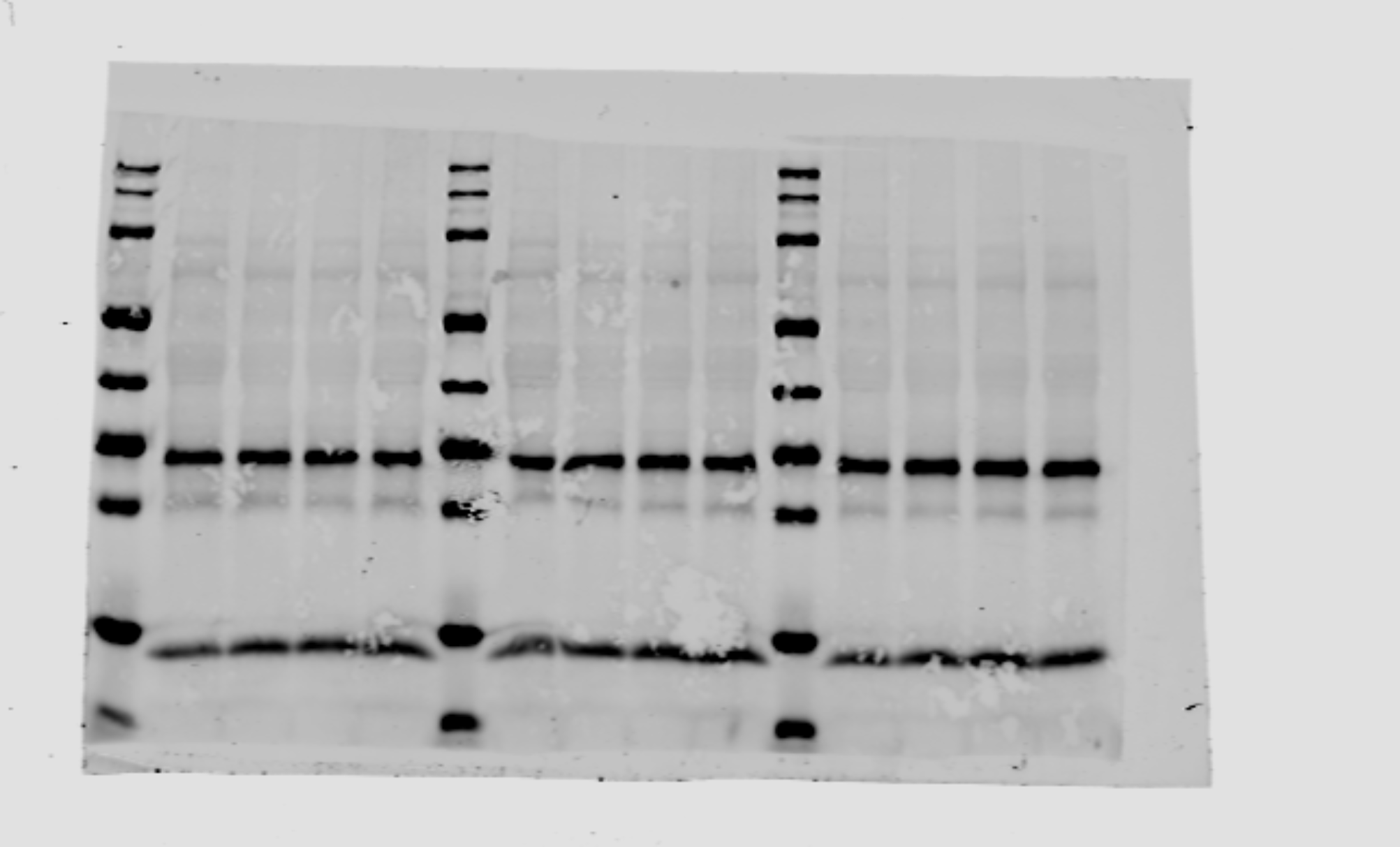

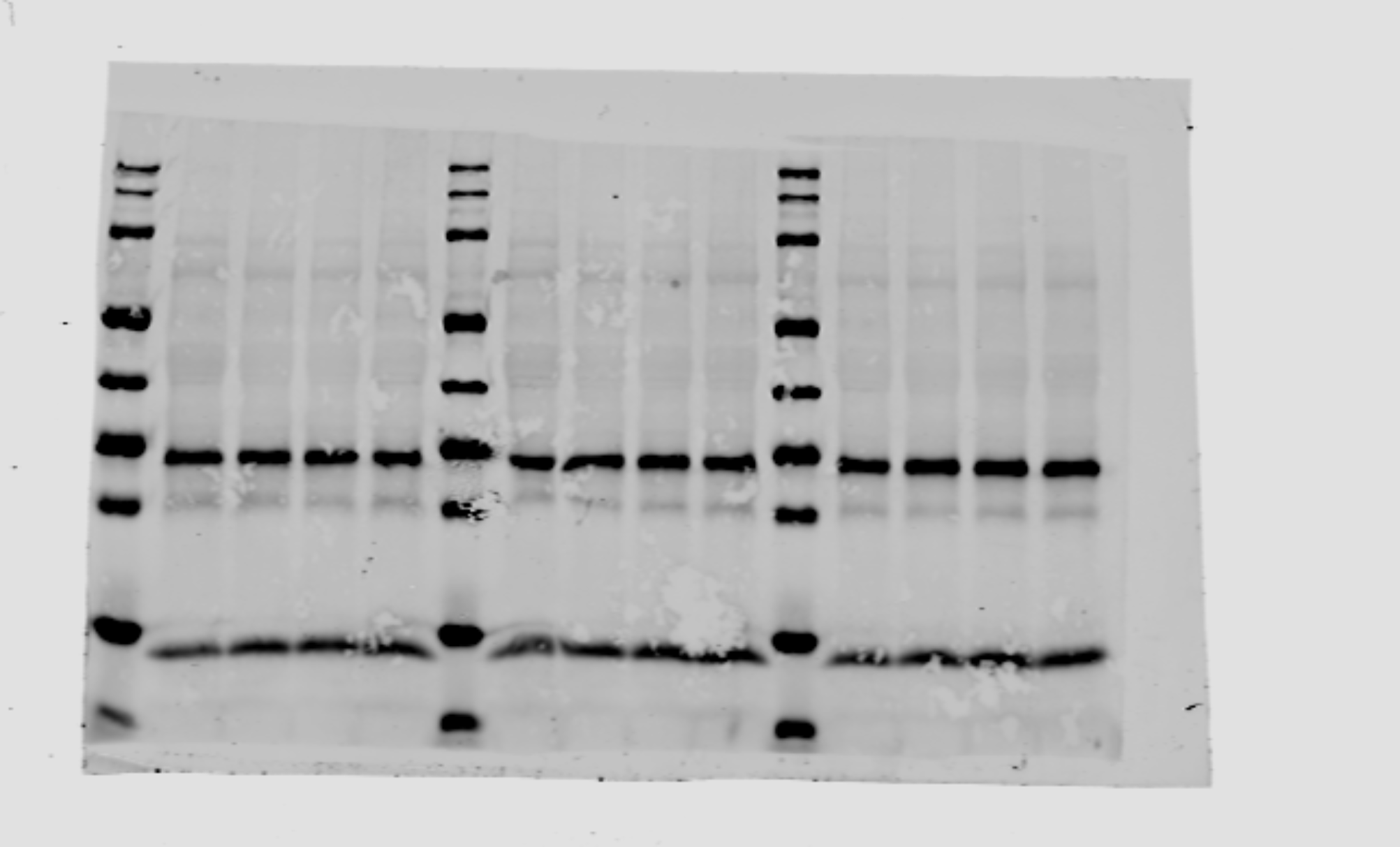

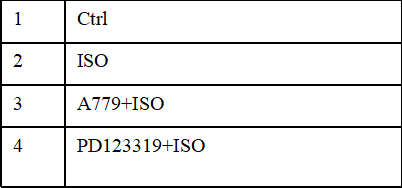


**15kDa**

**100kDa**

**10kDa**

**130kDa**

**180kDa**

**55kDa**

**35kDa**

**25kDa**

**70kDa**

**40kDa**

**1 2 3 4**

**BNP 15 kDa**

**GAPDH 36kDa**


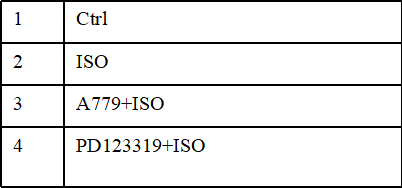


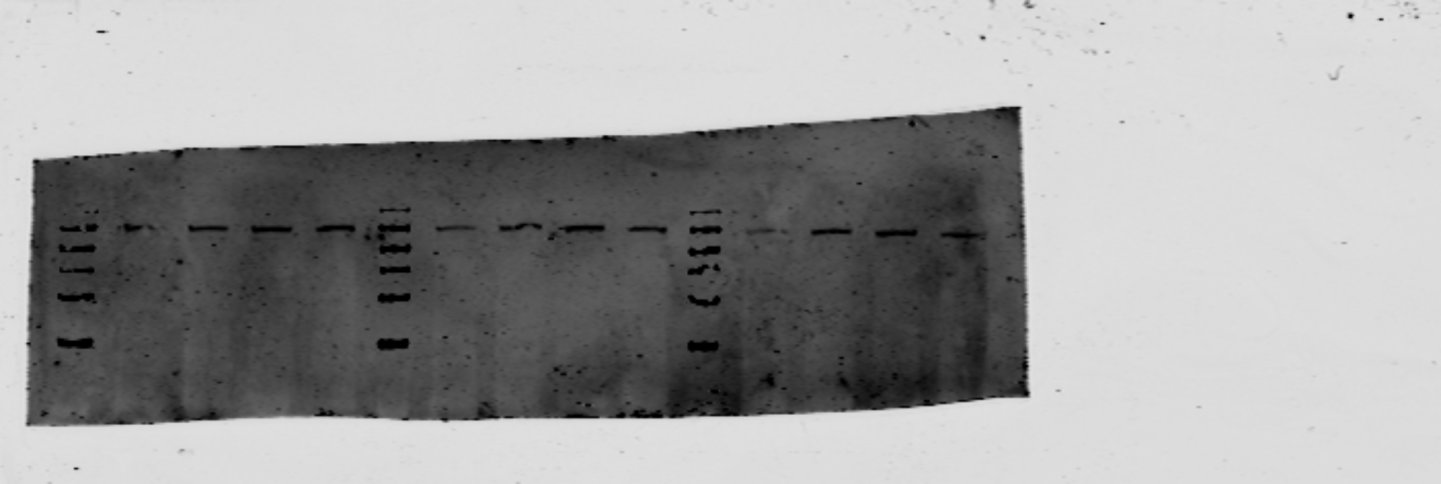

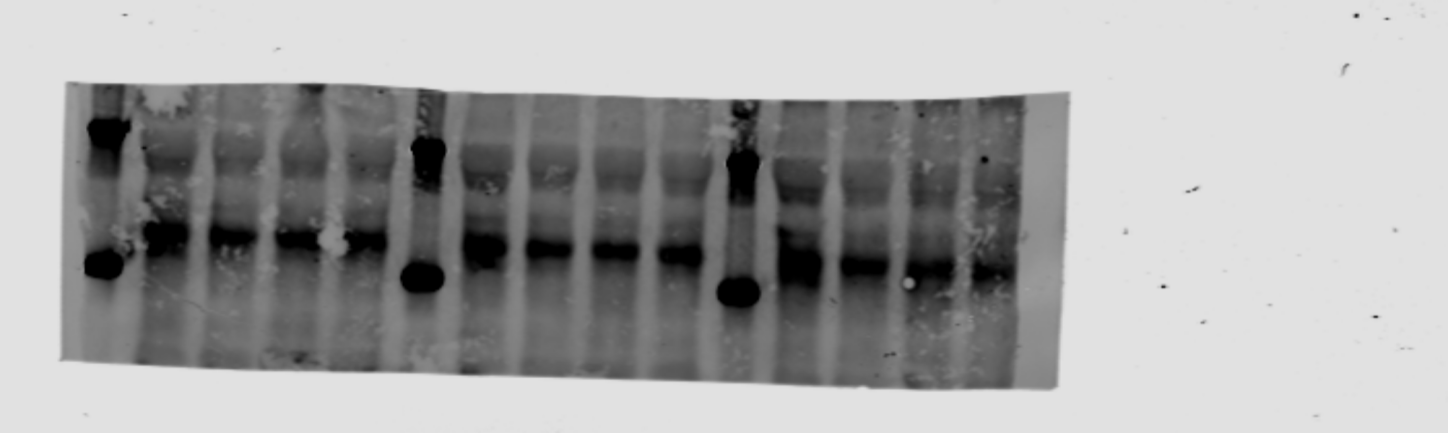


**1 2 3 4**

**β-MHC 223kDa**

**GAPDH 36kDa**

**250kDa**

**200kDa**

**160kDa**

**130kDa**

**100kDa**

**72kDa**

**49kDa**
